# Robot-assisted minimally invasive esophagectomy (RAMIE) vs. hybrid minimally invasive esophagectomy: propensity score matched short-term outcome analysis of a European high-volume center

**DOI:** 10.1007/s00464-022-09254-2

**Published:** 2022-05-03

**Authors:** Benjamin Babic, Dolores T. Müller, Jin-On Jung, Lars M. Schiffmann, Paula Grisar, Thomas Schmidt, Seung-Hun Chon, Wolfgang Schröder, Christiane J. Bruns, Hans F. Fuchs

**Affiliations:** 1 Department of General, Visceral, Cancer and Transplant Surgery, University of Cologne, Kerpener Strasse 62, 50937 Cologne, Germany; 2 Department of Trauma and Orthopedic Surgery, BG Unfallklinik Frankfurt am Main, Frankfurt am Main, Germany

**Keywords:** Esophageal cancer, RAMIE, Esophagectomy, MIE, Outcome

## Abstract

**Introduction:**

Transthoracic esophagectomy is a highly complex and sophisticated procedure with high morbidity rates and a significant mortality. Surgical access has consistently become less invasive, transitioning from open esophagectomy to hybrid esophagectomy (HE) then to totally minimally invasive esophagectomy (MIE), and most recently to robot-assisted minimally invasive esophagectomy (RAMIE), with each step demonstrating improved patient outcomes. Aim of this study with more than 600 patients is to complete a propensity-score matched comparison of postoperative short-term outcomes after highly standardized RAMIE vs. HE in a European high volume center.

**Patients and Methods:**

Six hundred and eleven patients that underwent transthoracic Ivor–Lewis esophagectomy for esophageal cancer between May 2016 and May 2021 were included in the study. In January 2019, we implemented an updated robotic standardized anastomotic technique using a circular stapler and ICG (indocyanine green) for RAMIE cases. Data were retrospectively analyzed from a prospectively maintained IRB-approved database. Outcomes of patients undergoing standardized RAMIE from January 2019 to May 2021 were compared to our overall cohort from May 2016–April 2021 (HE) after a propensity-score matching analysis was performed.

**Results:**

Six hundred and eleven patients were analyzed. 107 patients underwent RAMIE. Of these, a total of 76 patients underwent a robotic thoracic reconstruction using the updated standardized circular stapled anastomosis (RAMIE group). A total of 535 patients underwent HE (Hybrid group). Seventy patients were propensity-score matched in each group and analysis revealed no statistically significant differences in baseline characteristics. RAMIE patients had a significantly shorter ICU stay (*p* = 0.0218). Significantly more patients had no postoperative complications (Clavien Dindo 0) in the RAMIE group [47.1% vs. 27.1% in the HE group (*p* = 0.0225)]. No difference was seen in lymph node yield and R0 resection rates. Anastomotic leakage rates when matched were 14.3% in the hybrid group vs. 4.3% in the RAMIE group (*p* = 0.07).

**Conclusion:**

Our analysis confirms the safety and feasibility of RAMIE and HE in a large cohort after propensity score matching. A regular postoperative course (Clavien–Dindo 0) and a shorter ICU stay were seen significantly more often after RAMIE compared to HE. Furthermore it shows that both procedures provide excellent short-term oncologic outcomes, regarding lymph node harvest and R0 resection rates. A randomized controlled trial comparing RAMIE and HE is still pending and will hopefully contribute to ongoing discussions.

**Supplementary Information:**

The online version contains supplementary material available at 10.1007/s00464-022-09254-2.

Esophageal cancer (EC) is a common malignant disease with an increasing incidence and high mortality [1]. Transthoracic en-bloc esophagectomy with gastric pull-up was proven to be superior to a transhiatal approach and therefore depicts the gold standard in surgical treatment of EC. Patients with a locally advanced cancer usually receive a neoadjuvant treatment followed by surgery [2, 3]. Transthoracic Ivor–Lewis esophagectomy (IL-OE) with gastric pull-up and a high intrathoracic esophagogastric anastomosis is the standard surgical approach, according to guidelines and clinical practice [4, 5]. The high intrathoracic anastomosis is associated with decreased morbidity, especially regarding anastomotic leakage and recurrent nerve palsy compared to a cervical anastomosis in the McKeown procedure [6, 7]. Still, transthoracic esophagectomy is a highly complex and sophisticated procedure with morbidity rates greater than 50% and a significant mortality up to 5%.

Fortunately, several recent developments and technical improvements have led to improved postoperative results. One development has been a centralisation of this complex procedure to high volume centers, as international data has shown a clear correlation between annual case load to reduced morbidity rates in complex surgical procedures [8, 9]. Furthermore, the surgical technique has improved and surgical access has consistently become less invasive, transitioning from open esophagectomy to hybrid esophagectomy (HE) then to totally minimally invasive esophagectomy (MIE), and most recently to robot assisted minimally invasive esophagectomy (RAMIE), with each step demonstrating improved patient outcomes [10–15]. After the first robotic esophagectomy in 2003, initial case studies have shown acceptable results [16–18]. Since then, several trials have proven the feasibility and safety of RAMIE, which led to the implementation of this procedure in specialized and dedicated centers [12, 19, 20]. Recently, an international consensus on a training curriculum for further improvement of RAMIE outcomes, led by our research group, was published [21].

In particular anastomotic leakage has been shown to be one of the most crucial benchmarks for morbidity after esophageal surgery and can occur in up to 35% of the patients [22–27]. In a previous large cohort study, we reported that postoperative anastomotic leakage was independent of the size of the circular stapler. Furthermore, there was a trend towards better results after RAMIE [28].

The aim of this study is to complete a propensity score matched comparison of postoperative short-term outcomes after highly standardized RAMIE vs. HE in a European high volume center.

## Methods

A total of 611 patients that underwent transthoracic esophagectomy for EC at the Department of General, Visceral and Cancer Surgery, University of Cologne, between May 2016 and May 2021 were included in the study. Starting in January 2019, we implemented an updated robotic standardized anastomotic technique using a circular stapler and ICG for the thoracic part of all our RAMIE cases at our academic center. Outcomes of patients undergoing this procedure for EC from January 2019 to May 2021 were compared to our overall cohort from May 2016 to April 2021 (Hybrid group). Data was retrospectively analysed from a prospectively maintained database. The study was approved by the local Ethics Committee of the University of Cologne.

### Operative procedure

A transthoracic esophagectomy with two-field lymphadenectomy was performed in all cases. Reconstruction of the intestinal passage was done in all cases with a gastric tube and a high intrathoracic esophagogastrostomy [Ivor–Lewis procedure (IL-OE)]. The HE group was operated with laparoscopic gastrolysis and gastric pull up via thoracotomy. RAMIE was performed totally minimally invasive, a laparoscopic gastrolysis and a robot assisted thoracic approach using the Da Vinci X or the Da Vinci Xi System (Da Vinci X/Xi system, Intuitive Surgical Inc. Sunnyvale, CA, USA). In both groups the abdominal part was performed in a standardized way laparoscopically and technically identical. The gastric tube of 4 cm width was created intraabdominal with one 45 mm and three 60 mm Endo GIA™ reload units (Medtronic (Covidien) EEA 28 mm DST Circular Stapler, Medtronic GmbH Meerbusch/Germany). The sufficient blood supply via the gastroepiploic arcade has been safely documented in both groups with the usage of ICG during the abdominal part in a majority of cases. In the RAMIE group ICG was used in the thoracic part, as well. Anastomosis was performed in both groups as an end-to-side esophagogastrostomy with a circular stapler (Medtronic (Covidien) EEA 28 mm DST Circular Stapler, Medtronic GmbH Meerbusch/Germany). A 28 mm circular stapling device has been used routinely, and in cases with a narrow esophagus a 25 mm circular stapling device was used. After performing the circular anastomosis the excess portion of the gastric conduit was dissected with a further 60 mm Endo GIA™ reload unit. The technique has been previously described in detail [29]. The same, non robotic stapling devices have been used on both groups of patients.

### Selection of patients for RAMIE

Patients with a history of surgery of the right hemithorax were not eligible for the RAMIE procedure. Since only procedures after the completion of the learning curve were included in this study, there were no further criteria which led to exclusion for RAMIE. Selection for RAMIE was much more depending on availability of the surgical robot, being available twice per week in the beginning of the study period and daily since Q4 2020 for general surgery.

### Information about surgeons experience

All RAMIE procedures have been performed by two board certified surgeons after achieving proficiency with a rather short learning curve [30, 31]. Both surgeons had performed more than 100 HE before starting with RAMIE.

All HE procedures have been performed by five board certified surgeons with a history of more than a hundred Ivor–Lewis esophagectomies, each.

### Study endpoints

The two groups (RAMIE vs. HE) were compared, focusing on the rate of postoperative complications and anastomotic leakage in particular. Morbidity was classified using the Clavien–Dindo classification [32].

### Statistical analysis

Data management and imputation, as well as matching were all realized by Python 3.9 and with Visual Studio Code (Version 1.59) as the integrated development environment (IDE) of choice [33]. Patients who underwent RAMIE were compared to patients that underwent standard hybrid Ivor–Lewis esophagectomy. To account for missing data, case-specific and variable-specific missingness of more than 25% was excluded. Eventually, the overall missingness of the entire dataset was 2.0%. We performed multiple imputations with *n* = 1000 iterations via IterativeImputer from the Sci-kit learn package [34]. After multiple imputation, a propensity-score matching analysis was performed via Python package pymatch (adapted from the R package Matching) to reduce the effect of known confounders to a minimum [35]. As potential confounders, all variables were considered which were available before surgery and which were independent from study group determination to either robotic or HE. Through logistic regression, a propensity-score was calculated for each patient based on the baseline characteristics displayed in Table 2. Matched study groups were created using nearest-neighbor one-to-one matching without replacement. A threshold of 0.003 was chosen to prevent poor matches after optimizing the threshold and simultaneous maximization of retained proportion according to overlap. After matching, a comparison between RAMIE and HE was performed using Chi-square tests for binary data, Mann–Whitney *U* test for ordinal data and Student’s *t* test for continuous data. A *p* value of less than 0.05 was considered statistically significant. StataSE Version 15.0 (by StataCrop LLC, College Station, TX) was used for further statistical analysis after matching.

## Results

A total of 611 patients were analyzed. 76 patients underwent a robot assisted thoracic reconstruction using the updated standardized circular stapled anastomosis (RAMIE group). A total of 535 patients underwent a highly standardized hybrid procedure with a circular stapled anastomosis (hybrid group). Seventy patients were included in propensity-score matching for each group. Demographic and oncological data is shown in Table 1. Mean age was 63 years (range 46–79) in the robotic group and 63 years (range 33–91) in the hybrid group (*p* = 0.6377) before matching and a mean of 62.5 years (range 46 – 79) compared to a mean of 60.9 (range 33 – 80) after matching without any statistically significant difference (*p* = 0.3061). Mean BMI was 25.3 kg/m^2^ (range 15.6 kg/m^2^–35.4 kg/m^2^) in the RAMIE group vs. 26.9 kg/m^2^ (range 16.1 kg/m^2^–48.4 kg/m^2^), *p* = 0.0074 before matching. Propensity score matching resolved the statistically significant difference in BMI (*p* = 0.4418).

**Table 1:** Patients’ characteristics and oncological data

	RAMIE		Hybrid		*p* value Matched
	*N* (%)	*N* matched (%)	*N* (%)	*N* matched (%)	
Patients	76 (100)	70 (100)	535 (100)	70 (100)	–
Female	15 (19.7)	14 (20)	82 (15.3)	18 (25.7)	0.546
Pathology					
Adenocarcinoma	58 (76.3)	55 (78.6)	432 (80.7)	52 (74.3)	0.691
Squamous cell carcinoma	18 (23.7)	15 (21.4)	103 (19.3)	18 (25.7)	
Neoadjuvant treatment					
None	12 (15.8)	11 (15.7)	78 (14.6)	12 (17.1)	1
CROSS	41 (53.9)	39 (55.7)	312 (58.3)	39 (55.7)	1
FLOT	23 (30.3)	20 (28.6)	132 (24.7)	18 (25.7)	0.850
Other	0 (0)	0 (0)	13 (2.4)	1 (1.4)	1

### Perioperative characteristics RAMIE

Mean duration of surgery was 381 min (SD 64 min, 95% CI 365–396 min), mean blood loss was 230 ml (SD 99 ml, 95% CI 206–253 ml) and conversion to open thoracotomy occurred once in 71 cases (1.4%). Comparison of duration of single lung ventilation HE vs. RAME was performed showing a mean single lung ventilation time of 135 min compared to 190 min in the robot-assisted group (*p* < 0.0001).

### Preoperative risk factors

Table 2 shows preoperative comorbidities and test results before and after matching of the RAMIE and the hybrid group. After matching, no statistically significant difference in preoperative risk factors was seen in both groups. ASA class was a median of 1 (range 1–3) in both groups after matching and ECOG status was a median of 0 (range 0–1).

**Table 2:** Preoperative comorbidities, laboratory test results, and pulmonary function testing before and after propensity-score matching of patients undergoing a standardized Hybrid vs. standardized RAMIE procedure

	RAMIE		Hybrid		Matching parameters		
	*N–* (%)	*N* (%) matched	*N* (%)	*N* (%) matched	SD unmatched	SD matched	*p* value Matched
Patients	76 (100)	70 (100)	535 (100)	70 (100)	–	–	–
Preoperative comorbidities							
Arterial hypertension	30 (39.5)	27 (38.6)	306 (57.2)	21 (30)	0.33146	− 0.18002	0.373
Coronary artery disease	7 (9.2)	6 (8.6)	74 (13.8)	10 (14.3)	0.14465	0.17904	0.426
History of myocardial infarction	4 (5.3)	3 (4.3)	45 (8.4)	5 (7.1)	0.12458	0.12244	0.718
Coronary artery disease with past revascularization	3 (3.9)	2 (2.9)	39 (7.3)	6 (8.6)	0.14509	0.24629	0.275
Atrial fibrillation	6 (7.9)	5 (7.1)	42 (7.9)	8 (11.4)	− 0.00164	0.14701	0.562
PAD	1 (1.3)	1 (1.4)	21 (3.9)	1 (1.4)	0.16351	0	1
COPD	3 (3.9)	3 (4.3)	51 (9.5)	2 (2.9)	0.24018	− 0.07648	1
Diabetes	7 (9.2)	7 (10)	67 (12.5)	9 (12.9)	0.10623	0.08925	0.791
Liver disease	4 (5.3)	4 (5.7)	30 (5.6)	1 (1.4)	0.01513	− 0.23083	0.366
Weight loss > 10%	17 (22.4)	16 (22.9)	125 (23.4)	11 (15.7)	0.02363	− 0.18048	0.392
Preoperative test results [mean (SD)]							
Albumin (g/dl)	40.3 (3.6)	40.3 (3.4)	39.9 (4.4)	41.2 (4.8)	− 0.09092	0.23720	0.1628
Bilirubin (mg/dl)	0.43 (0.2)	0.44 (0.2)	0.47 (0.42)	0.43 (0.22)	0.06124	− 0.03724	0.8259
Creatinine (mg/dl)	0.83 (0.18)	0.82 (0.18)	0.88 (0.22)	0.82 (0.21)	0.26225	− 0.01680	0.9210
GFR (ml/min)	90.1 (18.9)	91 (18.5)	85.6 (18.2)	92.5 (21)	− 0.24062	0.07397	0.6624
Quick (%)	107.3 (12.4)	106.7 (12.7)	106.9 (15.5)	110.3 (15.2)	− 0.02653	0.25031	0.1409
Preoperative test results [*N* (%)]							
Leukocytes < 4.4	16 (21.1)	14 (20)	95 (17.8)	18 (25.7)	− 0.32273	0.13542	0.546
Platelets < 150,000	8 (10.5)	7 (10)	48 (9)	12 (17.1)	− 0.18053	0.20820	0.324
FEV 1 < 80%	12 (15.8)	10 (14.3)	123 (23)	14 (20)	0.18672	0.15097	0.502
VC < 80%	8 (10.5)	6 (8.6)	107 (20)	13 (18.6)	0.26493	0.29303	0.137

*SD* standardized difference, *PAD* peripheral artery disease, *COPD* chronic obstructive pulmonary disease, *GFR* glomerular filtration rate, *FEV* forced expiratory volume (in 1 s), *VC* vital capacity of the lung

### Postoperative complications/outcome

**Fig. 1:**
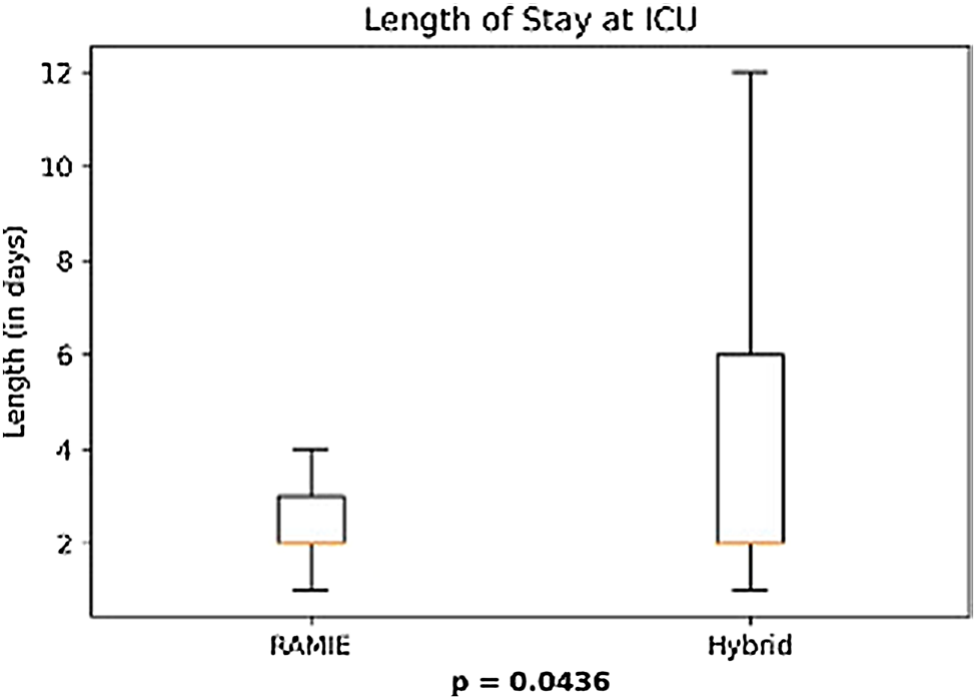
Patients that underwent a RAMIE procedure showed a significantly shorter ICU stay than patients that underwent a hybrid Ivor–Lewis esophagectomy

**Fig. 2:**
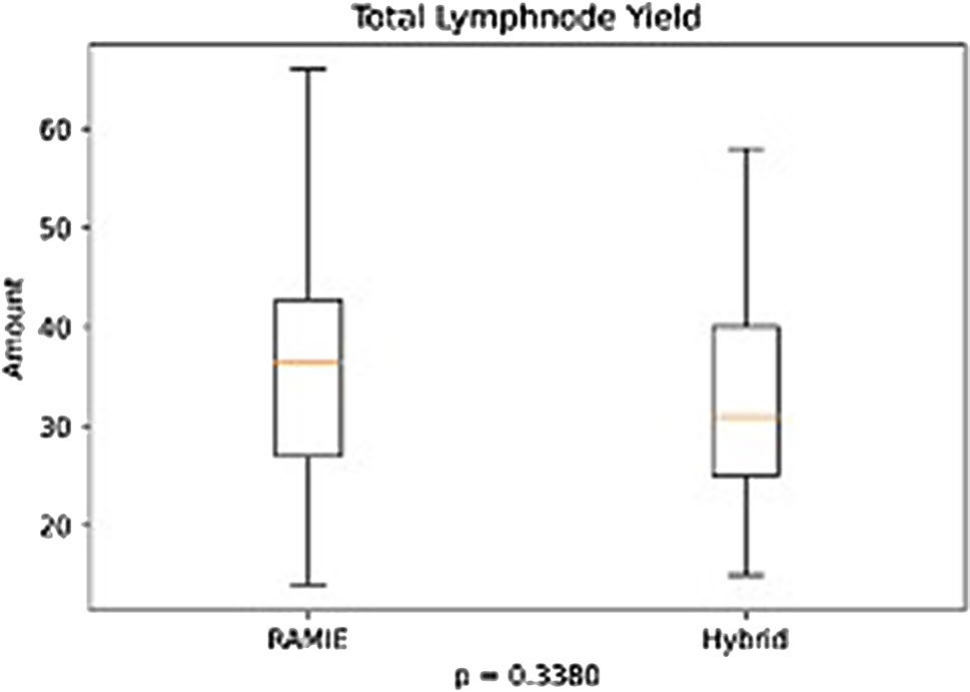
No significant difference was seen between groups in total lymph node yield

**Fig. 3:**
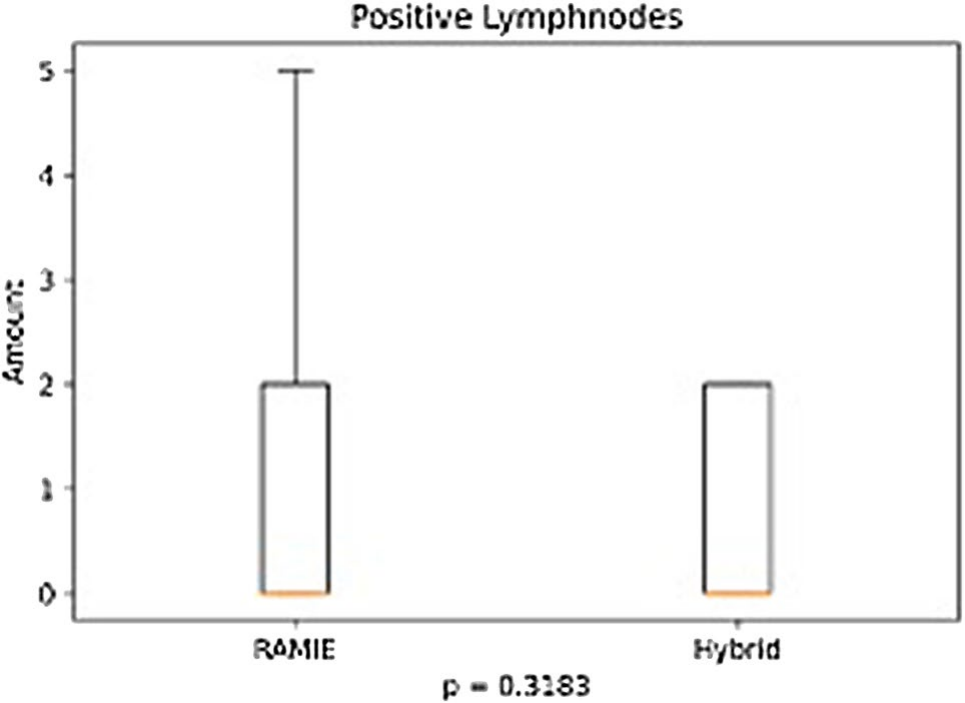
No significant difference was seen between groups in number of positive lymph nodes

**Fig. 4:**
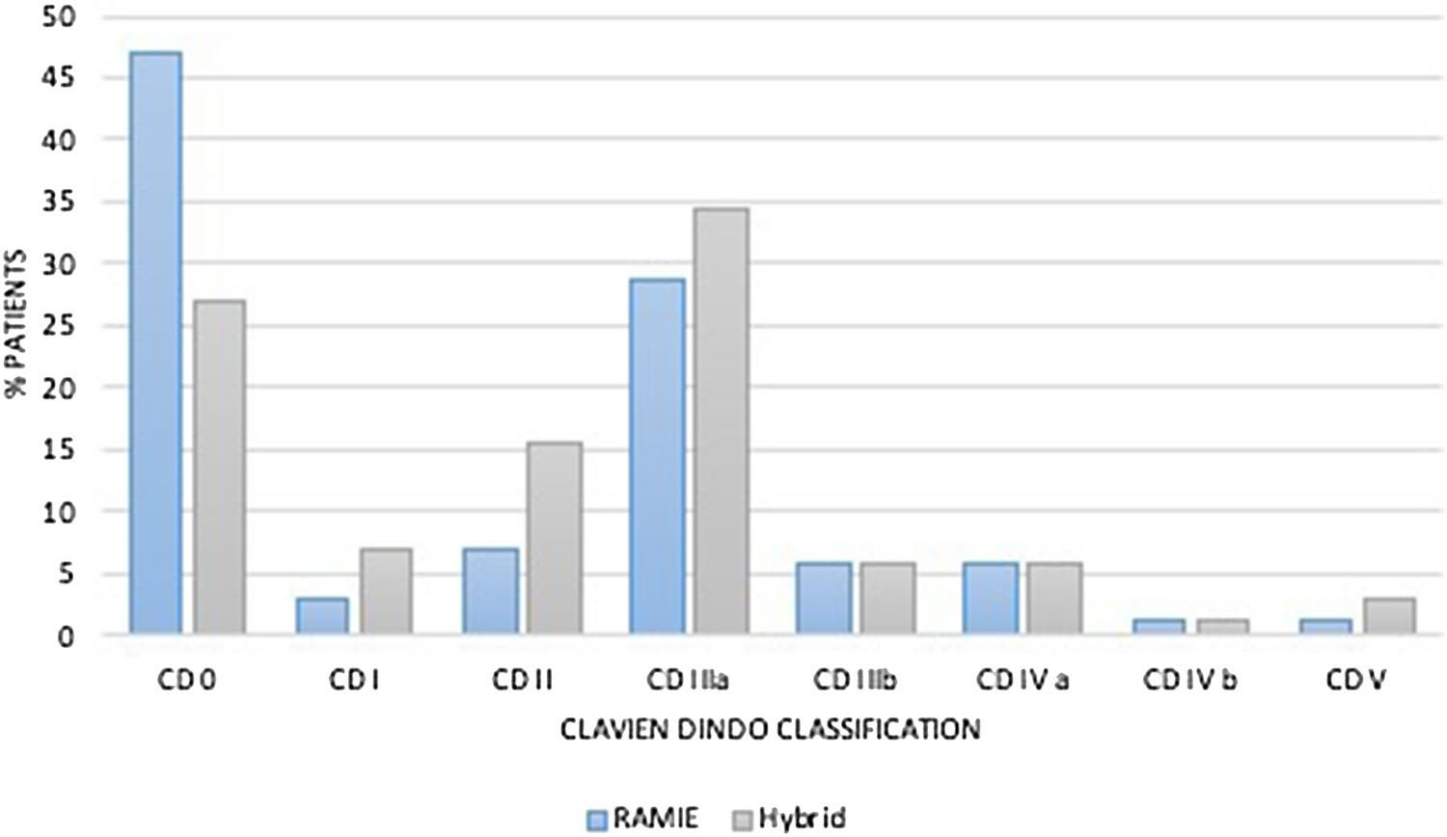
Postoperative morbidity and outcomes using the Clavien Dindo classification in patients that underwent an esophagectomy with a robotic approach vs. a hybrid approach

**Fig. 5:**
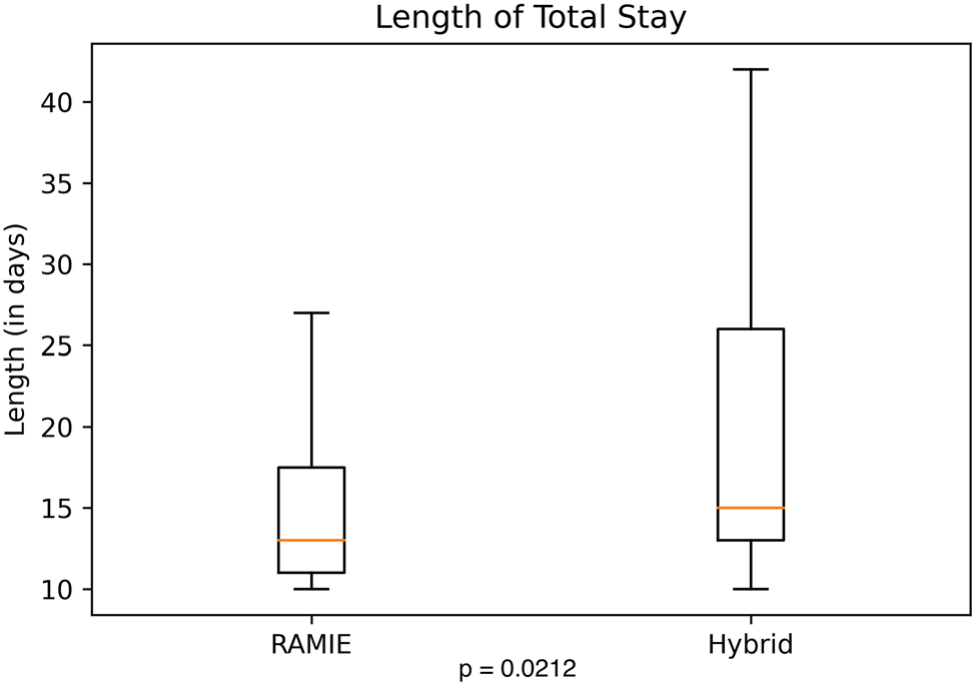
Patients that underwent a RAMIE procedure showed a significantly shorter hospital stay than patients that underwent a hybrid Ivor–Lewis esophagectomy

Further details on postoperative complications are depicted in Table 3 and Figs. 1, 2, 3, 4. RAMIE patients had a significantly shorter ICU stay (*p* = 0.0436) as well as a significantly shorter hospital stay (16 days in the RAMIE group, compared to 20 days in the hybrid group *p* = 0.0212) (Fig. 5). No difference was seen in lymph node yield and R0 resection rates, indicating equal quality of oncological surgery in both groups. Anastomotic leakage rates when matched were 14.3% in the hybrid group vs. 4.3% in the RAMIE group, approaching statistical significance (*p* = 0.07). Furthermore, significantly more patients had no postoperative complications (Clavien Dindo 0) in the RAMIE group (*p* = 0.0225), emphasizing again the non-inferiority of our robotic approach. Further analysis for occurrence of additional postoperative complications was performed. Postoperative atrial fibrillation occurred in 15.5% of the matched hybrid group compared to 9.9% of cases in the matched robot-assisted group (*p* = 0.313). Postoperative pneumonia occurred equally in both groups (8.5%, *p* = 1.000). In addition, an univariate and multivariate logistic regression analysis for all three complications (artrial fibrillation, pneumonia and anastomotic leakage) for the overall cohort and an ordinal logistic regression to investigate the final Clavien–Dindo score as another outcome parameter was performed. RAMIE was not an independent risk factor for atrial fibrillation (*p* = 0.763), pneumonia (*p* = 0.265) and anastomotic leakage (*p* = 0.341). However, robot-assisted surgery did show a significant influence on Clavien–Dindo scoring with an overall decrease of complications in the univariate analysis (coefficient = -0.469, *p* = 0.041). Nevertheless, this difference could not be maintained in the multivariate ordinal regression model (*p* = 0.124).

**Table 3:** Anastomotic leak types and severity of postoperative complications

	Robotic		Hybrid		*p* value Matched
	*N* (%)	*N* matched (%)	*N* (%)	*N* matched (%)	
Total	76 (100)	70 (100)	535 (100)	70 (100)	–
Anastomotic leak	6 (7.9)	3 (4.3)	59 (11)	10 (14.3)	0.077
ICU days mean (median)	3.2 (2)	3.2 (2)	5 (2)	4.9 (2)	0.0436
Clavien–Dindo classification					
CD 0	36 (47.4)	33 (47.1)	180 (33.6)	19 (27.1)	0.0225
CD I	2 (2.6)	2 (2.9)	28 (5.2)	5 (7.1)	0.4411
CD II	6 (7.9)	5 (7.1)	47 (8.8)	11 (15.7)	0.1829
CD IIIa	22 (28.9)	20 (28.6)	192 (35.9)	24 (34.3)	0.5852
CD IIIb	4 (5.3)	4 (5.7)	31 (5.8)	4 (5.7)	1
CD IVa	4 (5.3)	4 (5.7)	22 (4.1)	4 (5.7)	1
CD IVb	1 (1.3)	1 (1.4)	22 (4.1)	1 (1.4)	1
CD V	1 (1.3)	1 (1.4)	13 (2.4)	2 (2.9)	1
Resection status					
R0	70 (92.1)	65 (92.9)	516 (96.4)	67 (95.7)	0.7184
*N* [mean (SD)]	36.3	36.2 (14.3)	32.4	33.9 (13.5)	0.3380
*N* positive [mean (SD)]	2.3	2.4	2.4	3.1	0.4838

The severity of complications was significantly less in patients that underwent a standardized robotic Ivor Lewis esophagectomy

## Discussion

Our propensity-score matched analysis compared RAMIE to HE regarding short term outcomes in a European high volume center. Our study clearly demonstrates, that short term outcomes after RAMIE were non inferior compared to HE regarding morbidity and oncologic results. Particularly, our data even showed that postoperative ICU is significantly shorter after RAMIE (mean 3.2 days vs. 4.9 days after HE). This is reflected in the shorter hospital stay in general (16 days in the RAMIE group, compared to 20 days in the hybrid group *p* = 0.0212). Furthermore, the rate of Clavien–Dindo 0 after RAMIE was only 47% compared to 27% after HE (*p* = 0.0225), which even speaks for a patients’ benefit after RAMIE.

It is possible to consider that the retrospective and single center designed analysis are weaknesses of this study. However, there are several strengths. Over 600 cases were included within 5 years in our prospectively maintained database. The surgical technique in our center is highly standardized and therefore highly reproducible in both, HE and RAMIE. To account for the learning curve in robotic surgery, we only included patients whose surgery was performed by a surgeon who had completed an adequate number of RAMIE procedures in order to prevent an effect of the learning curve on the results and to counteract a possible procedure depending bias [30, 31, 36]. In our center, two surgeons fulfilled this criteria and they performed all RAMIE procedures included in this study. The size of our cohort allowed us to use rigid propensity matching criteria with an overall missingness of the whole dataset of only 2.0% after application of broad selection criteria. We chose a threshold of 0.003 to prevent poor matches and were still able to achieve a comparatively high retention of cases. Therefore, there were no significant differences in the baseline characteristics of the compared groups. All of this contributed to a high quality of data and a high number of matched patients. Other single center studies comparing minimally invasive surgical procedures in a propensity-score matched analysis included 100 and 211 patients, respectively [37, 38]. In one study, a larger cohort of about 700 cases was included. It should be noted that 4 large national centers contributed to the study and therefore the comparability of surgical techniques and postoperative management was limited [39].

A recent meta-analysis showed no difference in the resected lymph node yield after RAMIE compared to conventional MIE [40]. In our study the yield of resected lymph nodes with a mean of more than 30 in both groups proves the oncologic quality of the surgical procedure in both groups and surpasses international guideline requirements [41]. The total number of harvested lymph nodes did not differ significantly between both groups, RAMIE and HE.

The type of reconstruction, either cervical or intrathoracic anastomosis, has been the subject of discussion in the recent years [5, 42]. Van der Sluis et al. showed in a randomized controlled trial that RAMIE is a safe and feasible procedure. The fact that in their study cervical McKeown anastomosis was performed somehow counteracted a good comparability of the data to other cohorts [12]. Since our experience showed a benefit for the IL-OE previously, this technique is the gold standard in our center. Most actual data underline the choice for intrathoracic anastomosis and prove a significantly lower morbidity compared to cervical anastomosis after minimally invasive or HE [7]. We described earlier, that the stapler size (25 mm vs. 28 mm) does not significantly contribute to postoperative morbidity [28]. Even though a recent study described no benefit for the usage of ICG for the prevention of anastomotic leak after MIE our standard procedure for reconstruction in RAMIE is the usage of ICG before performing anastomosis [43]. With a leakage rate of only 4.3% after RAMIE in the matched group, we were able to show a trend towards a safer anastomosis compared to 14.3% after HE, however, without the difference to be statistically significant. This result was achieved, even though the surgical technique and the devices used for the creation of the gastric tube and performing the anastomosis were identical in both groups. Even more, the abdominal part was performed identically in both groups with a laparoscopic approach and an abdominal ICG usage for the documentation of a sufficient blood supply of the tube in a majority of cases. We think that this is a strength of our study, since the comparability of these crucial parts of the procedure is very high. It can be discussed, if the intrathoracic usage of ICG in the RAMIE group has the potential to lower the rates of anastomotic leakage. We have to mention, that we did not have a single case where we had to change the thoracic anastomotic site based on ICG signaling. This supports the findings of the previously mentioned metaanalysis showing no benefit of ICG usage in the prevention of anastomotic leakage [43]. Interestingly a meta-analysis from van Workum et al. described higher leakage rates and a lower lymph node harvest after totally MIE compared to HE [44].

In summary, the short term outcomes improved in favour of the RAMIE procedure. This might be due to a minimized thoracic access trauma, since very high surgical oncologic standards were fulfilled in both groups without any difference. A reduction of the thoracic access could consequence in a better pain control and furthermore might have the potential to impede a cooling down the patient compared to an open thorax. We have learned that even the reduction of the abdominal access trauma reduces the postoperative morbidity significantly after the MIRO-Trial [10]. It is unclear which immunologic effects might be affected with the surgical access trauma, since it has been described previously, that inflammatory parameters differ in the postoperative course depending on the procedure [45].

In conclusion, our analysis confirms the safety and feasibility of RAMIE and HE in a large cohort after propensity-score matching. A regular postoperative course (Clavien–Dindo 0) was seen significantly more often after RAMIE compared to HE. Furthermore it shows that both procedures provide excellent short-term oncologic outcomes, regarding lymph node harvest and R0 resection rates. A randomized controlled trial comparing RAMIE and HE is still pending and will hopefully contribute to ongoing discussions.

## Supplementary Information

Below is the link to the electronic supplementary material.

Supplementary file1 (PDF 48 kb)

Supplementary file2 (PDF 94 kb)

Supplementary file3 (PDF 29 kb)
