# Supplementary material for: Robot-assisted minimally invasive esophagectomy (RAMIE) vs. hybrid minimally invasive esophagectomy: propensity score matched short-term outcome analysis of a European high-volume center
Source: Surg Endosc. 2022 May 3;36(10):7747–55. doi: 10.1007/s00464-022-09254-2 (PMC9485091; doi:10.1007/s00464-022-09254-2)
Supplement: Supplementary file 1 — Supplementary file1 (PDF 48 kb) [file 464_2022_9254_MOESM1_ESM.pdf]

```
  ___  ____  ____  ____  ____ (R)
 /__    /   ____/   /   ____/
___/   /   /___/   /   /___/   15.0   Copyright 1985-2017 StataCorp LLC
  Statistics/Data Analysis            StataCorp
                                      4905 Lakeway Drive
     Special Edition                  College Station, Texas 77845 USA
                                      800-STATA-PC        http://www.stata.com
                                      979-696-4600        stata@stata.com
                                      979-696-4601 (fax)

-----------------------------BEFORE MATCHING----------------------------------

. ttest preop_age , by(psm_robot)

Two-sample t test with equal variances
------------------------------------------------------------------------------
   Group |     Obs        Mean    Std. Err.   Std. Dev.   [95% Conf. Interval]
---------+--------------------------------------------------------------------
       0 |     535    63.36064    .4279121    9.897635    62.52004    64.20123
       1 |      76    62.92105    .9352292     8.15314    61.05798    64.78412
---------+--------------------------------------------------------------------
combined |     611    63.30596    .3921321    9.692885    62.53586    64.07605
---------+--------------------------------------------------------------------
    diff |            .4395829    1.189043               -1.895539    2.774705
------------------------------------------------------------------------------
    diff = mean(0) - mean(1)                                      t =   0.3697
Ho: diff = 0                                     degrees of freedom =      609

    Ha: diff < 0                 Ha: diff != 0                 Ha: diff > 0
 Pr(T < t) = 0.6441         Pr(|T| > |t|) = 0.7117          Pr(T > t) = 0.3559

. ttest preop_height , by(psm_robot)

Two-sample t test with equal variances
------------------------------------------------------------------------------
   Group |     Obs        Mean    Std. Err.   Std. Dev.   [95% Conf. Interval]
---------+--------------------------------------------------------------------
       0 |     535    1.769428    .0037804    .0874405    1.762002    1.776854
       1 |      76    1.768289    .0085929    .0749113    1.751172    1.785407
---------+--------------------------------------------------------------------
combined |     611    1.769286    .0034762    .0859264     1.76246    1.776113
---------+--------------------------------------------------------------------
    diff |            .0011386    .0105418               -.0195642    .0218413
------------------------------------------------------------------------------
    diff = mean(0) - mean(1)                                      t =   0.1080
Ho: diff = 0                                     degrees of freedom =      609

    Ha: diff < 0                 Ha: diff != 0                 Ha: diff > 0
 Pr(T < t) = 0.5430         Pr(|T| > |t|) = 0.9140          Pr(T > t) = 0.4570

. ttest preop_weight , by(psm_robot)

Two-sample t test with equal variances
------------------------------------------------------------------------------
   Group |     Obs        Mean    Std. Err.   Std. Dev.   [95% Conf. Interval]
---------+--------------------------------------------------------------------
       0 |     535    84.62131    .7906777    18.28843    83.06809    86.17453
       1 |      76    79.30263    1.645011    14.34087     76.0256    82.57966
---------+--------------------------------------------------------------------
combined |     611    83.95974    .7250121    17.92115    82.53592    85.38356
---------+--------------------------------------------------------------------
    diff |            5.318677    2.188076                1.021587    9.615767
------------------------------------------------------------------------------
    diff = mean(0) - mean(1)                                      t =   2.4308
Ho: diff = 0                                     degrees of freedom =      609

    Ha: diff < 0                 Ha: diff != 0                 Ha: diff > 0
 Pr(T < t) = 0.9923         Pr(|T| > |t|) = 0.0154          Pr(T > t) = 0.0077

. ttest preop_bmi , by(psm_robot)

Two-sample t test with equal variances
------------------------------------------------------------------------------
   Group |     Obs        Mean    Std. Err.   Std. Dev.   [95% Conf. Interval]
---------+--------------------------------------------------------------------
       0 |     535    26.90336    .2133744    4.935363    26.48421    27.32252
       1 |      76    25.28916     .455627    3.972064     24.3815    26.19681
---------+--------------------------------------------------------------------
combined |     611    26.70258    .1963128    4.852541    26.31705    27.08811
---------+--------------------------------------------------------------------
    diff |            1.614207     .591732                .4521236     2.77629
------------------------------------------------------------------------------
    diff = mean(0) - mean(1)                                      t =   2.7279
Ho: diff = 0                                     degrees of freedom =      609

    Ha: diff < 0                 Ha: diff != 0                 Ha: diff > 0
 Pr(T < t) = 0.9967         Pr(|T| > |t|) = 0.0066          Pr(T > t) = 0.0033

. ttest preop_crea_tot , by(psm_robot)

Two-sample t test with equal variances
------------------------------------------------------------------------------
   Group |     Obs        Mean    Std. Err.   Std. Dev.   [95% Conf. Interval]
---------+--------------------------------------------------------------------
       0 |     535    .8835383    .0094569    .2187392     .864961    .9021157
       1 |      76      .83075    .0207628    .1810063    .7893883    .8721117
---------+--------------------------------------------------------------------
combined |     611    .8769722    .0086973    .2149834    .8598919    .8940525
---------+--------------------------------------------------------------------
    diff |            .0527883    .0262885                .0011613    .1044154
```

```
------------------------------------------------------------------------------
    diff = mean(0) - mean(1)                                      t =   2.0080
Ho: diff = 0                                     degrees of freedom =      609

    Ha: diff < 0                 Ha: diff != 0                 Ha: diff > 0
 Pr(T < t) = 0.9775         Pr(|T| > |t|) = 0.0451          Pr(T > t) = 0.0225

. ttest preop_gfr_tot , by(psm_robot)

Two-sample t test with equal variances
------------------------------------------------------------------------------
   Group |     Obs        Mean    Std. Err.   Std. Dev.   [95% Conf. Interval]
---------+--------------------------------------------------------------------
       0 |     535    85.59911    .7828956    18.10843    84.06118    87.13705
       1 |      76    90.04455    2.153981    18.77797     85.7536     94.3355
---------+--------------------------------------------------------------------
combined |     611    86.15206    .7377699     18.2365    84.70319    87.60094
---------+--------------------------------------------------------------------
    diff |           -4.44544     2.23009               -8.825041   -.0658397
------------------------------------------------------------------------------
    diff = mean(0) - mean(1)                                      t =  -1.9934
Ho: diff = 0                                     degrees of freedom =      609

    Ha: diff < 0                 Ha: diff != 0                 Ha: diff > 0
 Pr(T < t) = 0.0233         Pr(|T| > |t|) = 0.0467          Pr(T > t) = 0.9767

. ttest preop_quick_tot , by(psm_robot)

Two-sample t test with equal variances
------------------------------------------------------------------------------
   Group |     Obs        Mean    Std. Err.   Std. Dev.   [95% Conf. Interval]
---------+--------------------------------------------------------------------
       0 |     535    106.8875    .6595028    15.25434     105.592     108.183
       1 |      76      107.25    1.423503    12.40981    104.4142    110.0858
---------+--------------------------------------------------------------------
combined |     611    106.9326    .6036613    14.92155    105.7471    108.1181
---------+--------------------------------------------------------------------
    diff |           -.3624953    1.830599              -3.957548    3.232557
------------------------------------------------------------------------------
    diff = mean(0) - mean(1)                                      t =  -0.1980
Ho: diff = 0                                     degrees of freedom =      609

    Ha: diff < 0                 Ha: diff != 0                 Ha: diff > 0
 Pr(T < t) = 0.4215         Pr(|T| > |t|) = 0.8431          Pr(T > t) = 0.5785

. ttest preop_albumin_tot , by(psm_robot)

Two-sample t test with equal variances
------------------------------------------------------------------------------
   Group |     Obs        Mean    Std. Err.   Std. Dev.   [95% Conf. Interval]
---------+--------------------------------------------------------------------
       0 |     535    39.90129    .1856283    4.293594    39.53664    40.26594
       1 |      76    40.26274    .4071059    3.549067    39.45174    41.07373
---------+--------------------------------------------------------------------
combined |     611    39.94625    .1702076    4.207263    39.61198    40.28051
---------+--------------------------------------------------------------------
    diff |            -.361449    .5159623               -1.37473    .6518323
------------------------------------------------------------------------------
    diff = mean(0) - mean(1)                                      t =  -0.7005
Ho: diff = 0                                     degrees of freedom =      609

    Ha: diff < 0                 Ha: diff != 0                 Ha: diff > 0
 Pr(T < t) = 0.2419         Pr(|T| > |t|) = 0.4839          Pr(T > t) = 0.7581

. ttest preop_biliribin_tot , by(psm_robot)

Two-sample t test with equal variances
------------------------------------------------------------------------------
   Group |     Obs        Mean    Std. Err.   Std. Dev.   [95% Conf. Interval]
---------+--------------------------------------------------------------------
       0 |     535    .4455589    .0099258    .2295839    .4260605    .4650572
       1 |      76    .4324868    .0222062    .1935889    .3882499    .4767238
---------+--------------------------------------------------------------------
combined |     611    .4439329    .0091154     .225318    .4260315    .4618343
---------+--------------------------------------------------------------------
    diff |             .013072    .0276382              -.0412057    .0673497
------------------------------------------------------------------------------
    diff = mean(0) - mean(1)                                      t =   0.4730
Ho: diff = 0                                     degrees of freedom =      609

    Ha: diff < 0                 Ha: diff != 0                 Ha: diff > 0
 Pr(T < t) = 0.6818         Pr(|T| > |t|) = 0.6364          Pr(T > t) = 0.3182

. ttest preop_platelet_tot , by(psm_robot)

Two-sample t test with equal variances
------------------------------------------------------------------------------
   Group |     Obs        Mean    Std. Err.   Std. Dev.   [95% Conf. Interval]
---------+--------------------------------------------------------------------
       0 |     535    244.1798    3.630513    83.97401     237.048    251.3117
       1 |      76    238.0789     8.69934    75.83909     220.749    255.4089
---------+--------------------------------------------------------------------
combined |     611     243.421    3.356673    82.97162    236.8289     250.013
---------+--------------------------------------------------------------------
    diff |            6.100888    10.17641              -13.88423    26.08601
------------------------------------------------------------------------------
    diff = mean(0) - mean(1)                                      t =   0.5995
Ho: diff = 0                                     degrees of freedom =      609

    Ha: diff < 0                 Ha: diff != 0                 Ha: diff > 0
 Pr(T < t) = 0.7255         Pr(|T| > |t|) = 0.5491          Pr(T > t) = 0.2745
```

```
. ttest preop_leuko_tot , by(psm_robot)

Two-sample t test with equal variances
------------------------------------------------------------------------------
   Group |     Obs        Mean    Std. Err.   Std. Dev.   [95% Conf. Interval]
---------+--------------------------------------------------------------------
       0 |     535     6.39515    .1094007    2.530446    6.180241    6.610058
       1 |      76    5.996974    .2378569    2.073588    5.523138    6.470809
---------+--------------------------------------------------------------------
combined |     611    6.345622    .1003378     2.48019    6.148573    6.542671
---------+--------------------------------------------------------------------
    diff |            .3981758    .3038553               -.1985554    .9949071
------------------------------------------------------------------------------
    diff = mean(0) - mean(1)                                      t =   1.3104
Ho: diff = 0                                     degrees of freedom =      609

    Ha: diff < 0                 Ha: diff != 0                 Ha: diff > 0
 Pr(T < t) = 0.9047         Pr(|T| > |t|) = 0.1906          Pr(T > t) = 0.0953

. ttest preop_fev1_tot , by(psm_robot)

Two-sample t test with equal variances
------------------------------------------------------------------------------
   Group |     Obs        Mean    Std. Err.   Std. Dev.   [95% Conf. Interval]
---------+--------------------------------------------------------------------
       0 |     535    3.054955    .0342746    .7927748    2.987625    3.122285
       1 |      76    3.160789    .0774698    .6753661    3.006462    3.315117
---------+--------------------------------------------------------------------
combined |     611    3.068119    .0315318    .7794165    3.006195    3.130044
---------+--------------------------------------------------------------------
    diff |           -.1058343    .0955269               -.2934364    .0817677
------------------------------------------------------------------------------
    diff = mean(0) - mean(1)                                      t =  -1.1079
Ho: diff = 0                                     degrees of freedom =      609

    Ha: diff < 0                 Ha: diff != 0                 Ha: diff > 0
 Pr(T < t) = 0.1342         Pr(|T| > |t|) = 0.2683          Pr(T > t) = 0.8658

. ttest preop_vc_tot , by(psm_robot)

Two-sample t test with equal variances
------------------------------------------------------------------------------
   Group |     Obs        Mean    Std. Err.   Std. Dev.   [95% Conf. Interval]
---------+--------------------------------------------------------------------
       0 |     535    3.890011    .0405159    .9371364    3.810421    3.969601
       1 |      76    4.135974    .1009466    .8800325    3.934878     4.33707
---------+--------------------------------------------------------------------
combined |     611    3.920606    .0377481    .9330737    3.846474    3.994738
---------+--------------------------------------------------------------------
    diff |           -.2459625    .1140399               -.4699216   -.0220033
------------------------------------------------------------------------------
    diff = mean(0) - mean(1)                                      t =  -2.1568
Ho: diff = 0                                     degrees of freedom =      609

    Ha: diff < 0                 Ha: diff != 0                 Ha: diff > 0
 Pr(T < t) = 0.0157         Pr(|T| > |t|) = 0.0314          Pr(T > t) = 0.9843

.
. tabulate psm_robot preop_sex , chi2

           |       preop_sex
 psm_robot |         0          1 |     Total
-----------+----------------------+----------
         0 |       453         82 |       535
         1 |        61         15 |        76
-----------+----------------------+----------
     Total |       514         97 |       611

          Pearson chi2(1) =   0.9689   Pr = 0.325

. tabulate psm_robot preop_liver , chi2

           |      preop_liver
 psm_robot |         0          1 |     Total
-----------+----------------------+----------
         0 |       505         30 |       535
         1 |        72          4 |        76
-----------+----------------------+----------
     Total |       577         34 |       611

          Pearson chi2(1) =   0.0150   Pr = 0.902

. tabulate psm_robot preop_khk , chi2

           |       preop_khk
 psm_robot |         0          1 |     Total
-----------+----------------------+----------
         0 |       461         74 |       535
         1 |        69          7 |        76
-----------+----------------------+----------
     Total |       530         81 |       611

          Pearson chi2(1) =   1.2359   Pr = 0.266

. tabulate psm_robot preop_vhf , chi2

           |       preop_vhf
 psm_robot |         0          1 |     Total
-----------+----------------------+----------
         0 |       493         42 |       535
```

```
         1 |        70          6 |        76 
-----------+----------------------+----------
     Total |       563         48 |       611 

          Pearson chi2(1) =   0.0002   Pr = 0.989

. tabulate psm_robot preop_mi , chi2

           |       preop_mi
 psm_robot |         0          1 |     Total
-----------+----------------------+----------
         0 |       490         45 |       535 
         1 |        72          4 |        76 
-----------+----------------------+----------
     Total |       562         49 |       611 

          Pearson chi2(1) =   0.8940   Pr = 0.344

. tabulate psm_robot preop_revasc , chi2

           |     preop_revasc
 psm_robot |         0          1 |     Total
-----------+----------------------+----------
         0 |       496         39 |       535 
         1 |        73          3 |        76 
-----------+----------------------+----------
     Total |       569         42 |       611 

          Pearson chi2(1) =   1.1613   Pr = 0.281

. tabulate psm_robot preop_aht , chi2

           |       preop_aht
 psm_robot |         0          1 |     Total
-----------+----------------------+----------
         0 |       229        306 |       535 
         1 |        45         31 |        76 
-----------+----------------------+----------
     Total |       274        337 |       611 

          Pearson chi2(1) =   7.2423   Pr = 0.007

. tabulate psm_robot preop_pavk , chi2

           |      preop_pavk
 psm_robot |         0          1 |     Total
-----------+----------------------+----------
         0 |       514         21 |       535 
         1 |        75          1 |        76 
-----------+----------------------+----------
     Total |       589         22 |       611 

          Pearson chi2(1) =   1.3055   Pr = 0.253

. tabulate psm_robot preop_diabetes , chi2

           |    preop_diabetes
 psm_robot |         0          1 |     Total
-----------+----------------------+----------
         0 |       468         67 |       535 
         1 |        69          7 |        76 
-----------+----------------------+----------
     Total |       537         74 |       611 

          Pearson chi2(1) =   0.6861   Pr = 0.407

. tabulate psm_robot preop_weightloss_bin , chi2

           | preop_weightloss_bin
 psm_robot |         0          1 |     Total
-----------+----------------------+----------
         0 |       410        125 |       535 
         1 |        59         17 |        76 
-----------+----------------------+----------
     Total |       469        142 |       611 

          Pearson chi2(1) =   0.0370   Pr = 0.847

. tabulate psm_robot preop_diag , chi2

           |      preop_diag
 psm_robot |         0          1 |     Total
-----------+----------------------+----------
         0 |       432        103 |       535 
         1 |        58         18 |        76 
-----------+----------------------+----------
     Total |       490        121 |       611 

          Pearson chi2(1) =   0.8230   Pr = 0.364

. tabulate psm_robot preop_platelet_bin , chi2

           |  preop_platelet_bin
 psm_robot |         0          1 |     Total
-----------+----------------------+----------
         0 |       505         30 |       535 
         1 |        68          8 |        76 
-----------+----------------------+----------
     Total |       573         38 |       611 

          Pearson chi2(1) =   2.7605   Pr = 0.097
```

```
. tabulate psm_robot preop_leuko_bin , chi2

           |    preop_leuko_bin
 psm_robot |         0          1 |     Total
-----------+----------------------+----------
         0 |       484         51 |       535 
         1 |        60         16 |        76 
-----------+----------------------+----------
     Total |       544         67 |       611 

          Pearson chi2(1) =   9.0455   Pr = 0.003

. tabulate psm_robot preop_fev1_bin , chi2

           |    preop_fev1_bin
 psm_robot |         0          1 |     Total
-----------+----------------------+----------
         0 |       411        124 |       535 
         1 |        64         12 |        76 
-----------+----------------------+----------
     Total |       475        136 |       611 

          Pearson chi2(1) =   2.0991   Pr = 0.147

. tabulate psm_robot preop_vc_bin , chi2

           |     preop_vc_bin
 psm_robot |         0          1 |     Total
-----------+----------------------+----------
         0 |       428        107 |       535 
         1 |        68          8 |        76 
-----------+----------------------+----------
     Total |       496        115 |       611 

          Pearson chi2(1) =   3.9090   Pr = 0.048

. 
. ranksum preop_smoke , by(psm_robot)

Two-sample Wilcoxon rank-sum (Mann-Whitney) test

   psm_robot |      obs    rank sum    expected
-------------+---------------------------------
           0 |      535      166571      163710
           1 |       76       20395       23256
-------------+---------------------------------
    combined |      611      186966      186966

unadjusted variance  2073660.00
adjustment for ties  -312340.28
                     ----------
adjusted variance    1761319.72

Ho: preop~ke(psm_ro~t==0) = preop~ke(psm_ro~t==1)
             z =   2.156
    Prob > |z| =   0.0311

. ranksum preop_alcohol , by(psm_robot)

Two-sample Wilcoxon rank-sum (Mann-Whitney) test

   psm_robot |      obs    rank sum    expected
-------------+---------------------------------
           0 |      535      166451      163710
           1 |       76       20515       23256
-------------+---------------------------------
    combined |      611      186966      186966

unadjusted variance  2073660.00
adjustment for ties  -473888.75
                     ----------
adjusted variance    1599771.25

Ho: preop_~l(psm_ro~t==0) = preop_~l(psm_ro~t==1)
             z =   2.167
    Prob > |z| =   0.0302

. ranksum preop_neoadj , by(psm_robot)

Two-sample Wilcoxon rank-sum (Mann-Whitney) test

   psm_robot |      obs    rank sum    expected
-------------+---------------------------------
           0 |      535      165254      163710
           1 |       76       21712       23256
-------------+---------------------------------
    combined |      611      186966      186966

unadjusted variance  2073660.00
adjustment for ties  -440384.59
                     ----------
adjusted variance    1633275.41

Ho: preop_~j(psm_ro~t==0) = preop_~j(psm_ro~t==1)
             z =   1.208
    Prob > |z| =   0.2270

. ranksum preop_tstage , by(psm_robot)

Two-sample Wilcoxon rank-sum (Mann-Whitney) test
```

```
   psm_robot |      obs    rank sum    expected
-------------+---------------------------------
           0 |      535    164891.5      163710
           1 |       76     22074.5       23256
-------------+---------------------------------
    combined |      611      186966      186966

unadjusted variance  2073660.00
adjustment for ties   -1.18e+06
                     ----------
adjusted variance     893275.80

Ho: pre~tage(psm_ro~t==0) = pre~tage(psm_ro~t==1)
             z =   1.250
    Prob > |z| =   0.2113

. ranksum preop_asa , by(psm_robot)

Two-sample Wilcoxon rank-sum (Mann-Whitney) test

   psm_robot |      obs    rank sum    expected
-------------+---------------------------------
           0 |      535      153710      163710
           1 |       76       33256       23256
-------------+---------------------------------
    combined |      611      186966      186966

unadjusted variance  2073660.00
adjustment for ties  -346127.65
                     ----------
adjusted variance    1727532.35

Ho: preop_~a(psm_ro~t==0) = preop_~a(psm_ro~t==1)
             z =  -7.608
    Prob > |z| =   0.0000

. ranksum preop_ecog , by(psm_robot)

Two-sample Wilcoxon rank-sum (Mann-Whitney) test

   psm_robot |      obs    rank sum    expected
-------------+---------------------------------
           0 |      535      168959      163710
           1 |       76       18007       23256
-------------+---------------------------------
    combined |      611      186966      186966

unadjusted variance  2073660.00
adjustment for ties  -463553.89
                     ----------
adjusted variance    1610106.11

Ho: preop~og(psm_ro~t==0) = preop~og(psm_ro~t==1)
             z =   4.137
    Prob > |z| =   0.0000

. ranksum preop_copd , by(psm_robot)

Two-sample Wilcoxon rank-sum (Mann-Whitney) test

   psm_robot |      obs    rank sum    expected
-------------+---------------------------------
           0 |      535    164854.5      163710
           1 |       76     22111.5       23256
-------------+---------------------------------
    combined |      611      186966      186966

unadjusted variance  2073660.00
adjustment for ties   -1.57e+06
                     ----------
adjusted variance     501643.45

Ho: preop_~d(psm_ro~t==0) = preop_~d(psm_ro~t==1)
             z =   1.616
    Prob > |z| =   0.1061

.
```

```
  ___  ____  ____  ____  ____ (R)
 /__    /   ____/   /   ____/
___/   /   /___/   /   /___/   15.0   Copyright 1985-2017 StataCorp LLC
  Statistics/Data Analysis            StataCorp
                                      4905 Lakeway Drive
     Special Edition                  College Station, Texas 77845 USA
                                      800-STATA-PC        http://www.stata.com
                                      979-696-4600        stata@stata.com
                                      979-696-4601 (fax)

-----------------------------AFTER MATCHING-----------------------------------

. ttest preop_age , by(psm_robot)

Two-sample t test with equal variances
------------------------------------------------------------------------------
```

```
   Group |     Obs        Mean    Std. Err.   Std. Dev.   [95% Conf. Interval]
---------+--------------------------------------------------------------------
       0 |      71    62.83099    1.142905    9.630288    60.55153    65.11044
       1 |      71    62.59155    .9719564     8.18985    60.65304    64.53006
---------+--------------------------------------------------------------------
combined |     142    62.71127    .7475583    8.908185     61.2334    64.18914
---------+--------------------------------------------------------------------
    diff |            .2394366     1.50031               -2.726757    3.205631
------------------------------------------------------------------------------
    diff = mean(0) - mean(1)                                      t =   0.1596
Ho: diff = 0                                     degrees of freedom =      140

    Ha: diff < 0                 Ha: diff != 0                 Ha: diff > 0
 Pr(T < t) = 0.5633         Pr(|T| > |t|) = 0.8734          Pr(T > t) = 0.4367

. ttest preop_height , by(psm_robot)

Two-sample t test with equal variances
------------------------------------------------------------------------------
   Group |     Obs        Mean    Std. Err.   Std. Dev.   [95% Conf. Interval]
---------+--------------------------------------------------------------------
       0 |      71    1.758352      .01095    .0922664    1.736513    1.780191
       1 |      71    1.771268    .0087462    .0736969    1.753824    1.788711
---------+--------------------------------------------------------------------
combined |     142     1.76481    .0070034    .0834548    1.750965    1.778655
---------+--------------------------------------------------------------------
    diff |           -.0129155    .0140142               -.0406224    .0147914
------------------------------------------------------------------------------
    diff = mean(0) - mean(1)                                      t =  -0.9216
Ho: diff = 0                                     degrees of freedom =      140

    Ha: diff < 0                 Ha: diff != 0                 Ha: diff > 0
 Pr(T < t) = 0.1792         Pr(|T| > |t|) = 0.3583          Pr(T > t) = 0.8208

. ttest preop_weight , by(psm_robot)

Two-sample t test with equal variances
------------------------------------------------------------------------------
   Group |     Obs        Mean    Std. Err.   Std. Dev.   [95% Conf. Interval]
---------+--------------------------------------------------------------------
       0 |      71    86.10845     2.30304    19.40576    81.51518    90.70172
       1 |      71    80.30986    1.605554    13.52864    77.10768    83.51204
---------+--------------------------------------------------------------------
combined |     142    83.20915    1.419891    16.91996    80.40213    86.01618
---------+--------------------------------------------------------------------
    diff |            5.798592    2.807454                 .248104    11.34908
------------------------------------------------------------------------------
    diff = mean(0) - mean(1)                                      t =   2.0654
Ho: diff = 0                                     degrees of freedom =      140

    Ha: diff < 0                 Ha: diff != 0                 Ha: diff > 0
 Pr(T < t) = 0.9796         Pr(|T| > |t|) = 0.0407          Pr(T > t) = 0.0204

. ttest preop_bmi , by(psm_robot)

Two-sample t test with equal variances
------------------------------------------------------------------------------
   Group |     Obs        Mean    Std. Err.   Std. Dev.   [95% Conf. Interval]
---------+--------------------------------------------------------------------
       0 |      71    27.74558    .6651121    5.604335    26.41905     29.0721
       1 |      71    25.55742    .4542892    3.827909    24.65137    26.46347
---------+--------------------------------------------------------------------
combined |     142     26.6515     .411737    4.906412    25.83752    27.46548
---------+--------------------------------------------------------------------
    diff |            2.188155    .8054519                .5957332    3.780577
------------------------------------------------------------------------------
    diff = mean(0) - mean(1)                                      t =   2.7167
Ho: diff = 0                                     degrees of freedom =      140

    Ha: diff < 0                 Ha: diff != 0                 Ha: diff > 0
 Pr(T < t) = 0.9963         Pr(|T| > |t|) = 0.0074          Pr(T > t) = 0.0037

. ttest preop_crea_tot , by(psm_robot)

Two-sample t test with equal variances
------------------------------------------------------------------------------
   Group |     Obs        Mean    Std. Err.   Std. Dev.   [95% Conf. Interval]
---------+--------------------------------------------------------------------
       0 |      71    .8885915    .0236459    .1992435    .8414314    .9357517
       1 |      71    .8395352     .021503    .1811879    .7966487    .8824217
---------+--------------------------------------------------------------------
combined |     142    .8640634    .0160572    .1913432    .8323195    .8958073
---------+--------------------------------------------------------------------
    diff |            .0490563     .031961               -.0141323     .112245
------------------------------------------------------------------------------
    diff = mean(0) - mean(1)                                      t =   1.5349
Ho: diff = 0                                     degrees of freedom =      140

    Ha: diff < 0                 Ha: diff != 0                 Ha: diff > 0
 Pr(T < t) = 0.9365         Pr(|T| > |t|) = 0.1271          Pr(T > t) = 0.0635

. ttest preop_gfr_tot , by(psm_robot)

Two-sample t test with equal variances
------------------------------------------------------------------------------
   Group |     Obs        Mean    Std. Err.   Std. Dev.   [95% Conf. Interval]
---------+--------------------------------------------------------------------
       0 |      71    85.09634    1.922689    16.20087    81.26166    88.93102
       1 |      71    89.86459    2.274332    19.16386    85.32858     94.4006
---------+--------------------------------------------------------------------
combined |     142    87.48046    1.497303    17.84243     84.5204    90.44053
```

```
---------+--------------------------------------------------------------------
    diff |          -4.768254    2.978141               -10.6562     1.11969
------------------------------------------------------------------------------
    diff = mean(0) - mean(1)                                      t =  -1.6011
Ho: diff = 0                                     degrees of freedom =      140

    Ha: diff < 0                 Ha: diff != 0                 Ha: diff > 0
 Pr(T < t) = 0.0558         Pr(|T| > |t|) = 0.1116          Pr(T > t) = 0.9442

. ttest preop_quick_tot , by(psm_robot)

Two-sample t test with equal variances
------------------------------------------------------------------------------
   Group |     Obs        Mean    Std. Err.   Std. Dev.   [95% Conf. Interval]
---------+--------------------------------------------------------------------
       0 |      71    108.0423    1.852448    15.60901    104.3477    111.7368
       1 |      71    107.5352    1.201748    10.12611    105.1384     109.932
---------+--------------------------------------------------------------------
combined |     142    107.7887    1.100341    13.11208    105.6134     109.964
---------+--------------------------------------------------------------------
    diff |           .5070423    2.208113              -3.858515      4.8726
------------------------------------------------------------------------------
    diff = mean(0) - mean(1)                                      t =   0.2296
Ho: diff = 0                                     degrees of freedom =      140

    Ha: diff < 0                 Ha: diff != 0                 Ha: diff > 0
 Pr(T < t) = 0.5906         Pr(|T| > |t|) = 0.8187          Pr(T > t) = 0.4094

. ttest preop_albumin_tot , by(psm_robot)

Two-sample t test with equal variances
------------------------------------------------------------------------------
   Group |     Obs        Mean    Std. Err.   Std. Dev.   [95% Conf. Interval]
---------+--------------------------------------------------------------------
       0 |      71    40.27406    .4747842    4.000602    39.32713    41.22098
       1 |      71    40.29532    .4223973    3.559183    39.45288    41.13777
---------+--------------------------------------------------------------------
combined |     142    40.28469    .3166143    3.772895    39.65877    40.91061
---------+--------------------------------------------------------------------
    diff |          -.0212676    .6354836              -1.277653    1.235118
------------------------------------------------------------------------------
    diff = mean(0) - mean(1)                                      t =  -0.0335
Ho: diff = 0                                     degrees of freedom =      140

    Ha: diff < 0                 Ha: diff != 0                 Ha: diff > 0
 Pr(T < t) = 0.4867         Pr(|T| > |t|) = 0.9734          Pr(T > t) = 0.5133

. ttest preop_biliribin_tot , by(psm_robot)

Two-sample t test with equal variances
------------------------------------------------------------------------------
   Group |     Obs        Mean    Std. Err.   Std. Dev.   [95% Conf. Interval]
---------+--------------------------------------------------------------------
       0 |      71    .4756338    .0342012    .2881845    .4074217     .543846
       1 |      71        .439     .023104    .1946781    .3929204    .4850796
---------+--------------------------------------------------------------------
combined |     142    .4573169    .0206213    .2457314      .41655    .4980838
---------+--------------------------------------------------------------------
    diff |           .0366338    .0412737              -.0449665    .1182341
------------------------------------------------------------------------------
    diff = mean(0) - mean(1)                                      t =   0.8876
Ho: diff = 0                                     degrees of freedom =      140

    Ha: diff < 0                 Ha: diff != 0                 Ha: diff > 0
 Pr(T < t) = 0.8119         Pr(|T| > |t|) = 0.3763          Pr(T > t) = 0.1881

. ttest preop_platelet_tot , by(psm_robot)

Two-sample t test with equal variances
------------------------------------------------------------------------------
   Group |     Obs        Mean    Std. Err.   Std. Dev.   [95% Conf. Interval]
---------+--------------------------------------------------------------------
       0 |      71    268.2958    14.00975    118.0482    240.3542    296.2373
       1 |      71    240.1268    9.207182     77.5811    221.7636    258.4899
---------+--------------------------------------------------------------------
combined |     142    254.2113    8.436225    100.5292    237.5334    270.8891
---------+--------------------------------------------------------------------
    diff |           28.16901     16.7644              -4.975111    61.31314
------------------------------------------------------------------------------
    diff = mean(0) - mean(1)                                      t =   1.6803
Ho: diff = 0                                     degrees of freedom =      140

    Ha: diff < 0                 Ha: diff != 0                 Ha: diff > 0
 Pr(T < t) = 0.9524         Pr(|T| > |t|) = 0.0951          Pr(T > t) = 0.0476

. ttest preop_leuko_tot , by(psm_robot)

Two-sample t test with equal variances
------------------------------------------------------------------------------
   Group |     Obs        Mean    Std. Err.   Std. Dev.   [95% Conf. Interval]
---------+--------------------------------------------------------------------
       0 |      71    6.717606    .3803583    3.204956    5.959005    7.476206
       1 |      71    6.078732     .239684    2.019613    5.600698    6.556767
---------+--------------------------------------------------------------------
combined |     142    6.398169    .2256004    2.688339    5.952173    6.844165
---------+--------------------------------------------------------------------
    diff |           .6388732    .4495785              -.2499676    1.527714
------------------------------------------------------------------------------
    diff = mean(0) - mean(1)                                      t =   1.4210
Ho: diff = 0                                     degrees of freedom =      140
```

```
    Ha: diff < 0                 Ha: diff != 0                 Ha: diff > 0
 Pr(T < t) = 0.9212         Pr(|T| > |t|) = 0.1575          Pr(T > t) = 0.0788

. ttest preop_fev1_tot , by(psm_robot)

Two-sample t test with equal variances
------------------------------------------------------------------------------
   Group |     Obs        Mean    Std. Err.   Std. Dev.   [95% Conf. Interval]
---------+--------------------------------------------------------------------
       0 |      71    3.119901    .0870921    .7338513    2.946202    3.293601
       1 |      71    3.203944    .0772152    .6506271    3.049943    3.357945
---------+--------------------------------------------------------------------
combined |     142    3.161923    .0580974    .6923107    3.047068    3.276777
---------+--------------------------------------------------------------------
    diff |           -.0840423    .1163926               -.3141566    .1460721
------------------------------------------------------------------------------
    diff = mean(0) - mean(1)                                      t =  -0.7221
Ho: diff = 0                                     degrees of freedom =      140

    Ha: diff < 0                 Ha: diff != 0                 Ha: diff > 0
 Pr(T < t) = 0.2357         Pr(|T| > |t|) = 0.4715          Pr(T > t) = 0.7643

. ttest preop_vc_tot , by(psm_robot)

Two-sample t test with equal variances
------------------------------------------------------------------------------
   Group |     Obs        Mean    Std. Err.   Std. Dev.   [95% Conf. Interval]
---------+--------------------------------------------------------------------
       0 |      71    4.155972    .1274106    1.073581    3.901859    4.410084
       1 |      71    4.182451    .1026649    .8650695    3.977692    4.387209
---------+--------------------------------------------------------------------
combined |     142    4.169211    .0815301    .9715431    4.008032    4.330391
---------+--------------------------------------------------------------------
    diff |           -.0264789    .1636262               -.3499766    .2970189
------------------------------------------------------------------------------
    diff = mean(0) - mean(1)                                      t =  -0.1618
Ho: diff = 0                                     degrees of freedom =      140

    Ha: diff < 0                 Ha: diff != 0                 Ha: diff > 0
 Pr(T < t) = 0.4358         Pr(|T| > |t|) = 0.8717          Pr(T > t) = 0.5642

.
. tabulate psm_robot preop_sex , chi2

           |       preop_sex
 psm_robot |         0          1 |     Total
-----------+----------------------+----------
         0 |        60         11 |        71
         1 |        59         12 |        71
-----------+----------------------+----------
     Total |       119         23 |       142

          Pearson chi2(1) =   0.0519   Pr = 0.820

. tabulate psm_robot preop_liver , chi2

           |      preop_liver
 psm_robot |         0          1 |     Total
-----------+----------------------+----------
         0 |        66          5 |        71
         1 |        67          4 |        71
-----------+----------------------+----------
     Total |       133          9 |       142

          Pearson chi2(1) =   0.1186   Pr = 0.731

. tabulate psm_robot preop_khk , chi2

           |       preop_khk
 psm_robot |         0          1 |     Total
-----------+----------------------+----------
         0 |        66          5 |        71
         1 |        64          7 |        71
-----------+----------------------+----------
     Total |       130         12 |       142

          Pearson chi2(1) =   0.3641   Pr = 0.546

. tabulate psm_robot preop_vhf , chi2

           |       preop_vhf
 psm_robot |         0          1 |     Total
-----------+----------------------+----------
         0 |        69          2 |        71
         1 |        66          5 |        71
-----------+----------------------+----------
     Total |       135          7 |       142

          Pearson chi2(1) =   1.3524   Pr = 0.245

. tabulate psm_robot preop_mi , chi2

           |       preop_mi
 psm_robot |         0          1 |     Total
-----------+----------------------+----------
         0 |        68          3 |        71
         1 |        67          4 |        71
-----------+----------------------+----------
     Total |       135          7 |       142

          Pearson chi2(1) =   0.1503   Pr = 0.698
```

```
. tabulate psm_robot preop_revasc , chi2

           |     preop_revasc
 psm_robot |         0          1 |     Total
-----------+----------------------+----------
         0 |        69          2 |        71 
         1 |        68          3 |        71 
-----------+----------------------+----------
     Total |       137          5 |       142 

          Pearson chi2(1) =   0.2073   Pr = 0.649

. tabulate psm_robot preop_aht , chi2

           |       preop_aht
 psm_robot |         0          1 |     Total
-----------+----------------------+----------
         0 |        34         37 |        71 
         1 |        41         30 |        71 
-----------+----------------------+----------
     Total |        75         67 |       142 

          Pearson chi2(1) =   1.3847   Pr = 0.239

. tabulate psm_robot preop_pavk , chi2

           |      preop_pavk
 psm_robot |         0          1 |     Total
-----------+----------------------+----------
         0 |        71          0 |        71 
         1 |        70          1 |        71 
-----------+----------------------+----------
     Total |       141          1 |       142 

          Pearson chi2(1) =   1.0071   Pr = 0.316

. tabulate psm_robot preop_diabetes , chi2

           |    preop_diabetes
 psm_robot |         0          1 |     Total
-----------+----------------------+----------
         0 |        64          7 |        71 
         1 |        64          7 |        71 
-----------+----------------------+----------
     Total |       128         14 |       142 

          Pearson chi2(1) =   0.0000   Pr = 1.000

. tabulate psm_robot preop_weightloss_bin , chi2

           | preop_weightloss_bin
 psm_robot |         0          1 |     Total
-----------+----------------------+----------
         0 |        62          9 |        71 
         1 |        55         16 |        71 
-----------+----------------------+----------
     Total |       117         25 |       142 

          Pearson chi2(1) =   2.3788   Pr = 0.123

. tabulate psm_robot preop_diag , chi2

           |      preop_diag
 psm_robot |         0          1 |     Total
-----------+----------------------+----------
         0 |        63          8 |        71 
         1 |        57         14 |        71 
-----------+----------------------+----------
     Total |       120         22 |       142 

          Pearson chi2(1) =   1.9364   Pr = 0.164

. tabulate psm_robot preop_platelet_bin , chi2

           |  preop_platelet_bin
 psm_robot |         0          1 |     Total
-----------+----------------------+----------
         0 |        65          6 |        71 
         1 |        63          8 |        71 
-----------+----------------------+----------
     Total |       128         14 |       142 

          Pearson chi2(1) =   0.3170   Pr = 0.573

. tabulate psm_robot preop_leuko_bin , chi2

           |    preop_leuko_bin
 psm_robot |         0          1 |     Total
-----------+----------------------+----------
         0 |        59         12 |        71 
         1 |        58         13 |        71 
-----------+----------------------+----------
     Total |       117         25 |       142 

          Pearson chi2(1) =   0.0485   Pr = 0.826

. tabulate psm_robot preop_fev1_bin , chi2

           |    preop_fev1_bin
 psm_robot |         0          1 |     Total
```

```
-----------+----------------------+----------
         0 |        54         17 |        71 
         1 |        60         11 |        71 
-----------+----------------------+----------
     Total |       114         28 |       142 

          Pearson chi2(1) =   1.6015   Pr = 0.206

. tabulate psm_robot preop_vc_bin , chi2

           |     preop_vc_bin
 psm_robot |         0          1 |     Total
-----------+----------------------+----------
         0 |        66          5 |        71 
         1 |        63          8 |        71 
-----------+----------------------+----------
     Total |       129         13 |       142 

          Pearson chi2(1) =   0.7621   Pr = 0.383

. 
. ranksum preop_smoke , by(psm_robot)

Two-sample Wilcoxon rank-sum (Mann-Whitney) test

   psm_robot |      obs    rank sum    expected
-------------+---------------------------------
           0 |       71      4821.5      5076.5
           1 |       71      5331.5      5076.5
-------------+---------------------------------
    combined |      142       10153       10153

unadjusted variance    60071.92
adjustment for ties    -9138.23
                     ----------
adjusted variance      50933.69

Ho: preop~ke(psm_ro~t==0) = preop~ke(psm_ro~t==1)
             z =  -1.130
    Prob > |z| =   0.2585

. ranksum preop_alcohol , by(psm_robot)

Two-sample Wilcoxon rank-sum (Mann-Whitney) test

   psm_robot |      obs    rank sum    expected
-------------+---------------------------------
           0 |       71      5208.5      5076.5
           1 |       71      4944.5      5076.5
-------------+---------------------------------
    combined |      142       10153       10153

unadjusted variance    60071.92
adjustment for ties   -21057.92
                     ----------
adjusted variance      39014.00

Ho: preop_~l(psm_ro~t==0) = preop_~l(psm_ro~t==1)
             z =   0.668
    Prob > |z| =   0.5039

. ranksum preop_neoadj , by(psm_robot)

Two-sample Wilcoxon rank-sum (Mann-Whitney) test

   psm_robot |      obs    rank sum    expected
-------------+---------------------------------
           0 |       71        4514      5076.5
           1 |       71        5639      5076.5
-------------+---------------------------------
    combined |      142       10153       10153

unadjusted variance    60071.92
adjustment for ties    -7840.59
                     ----------
adjusted variance      52231.33

Ho: preop_~j(psm_ro~t==0) = preop_~j(psm_ro~t==1)
             z =  -2.461
    Prob > |z| =   0.0138

. ranksum preop_tstage , by(psm_robot)

Two-sample Wilcoxon rank-sum (Mann-Whitney) test

   psm_robot |      obs    rank sum    expected
-------------+---------------------------------
           0 |       71      4680.5      5076.5
           1 |       71      5472.5      5076.5
-------------+---------------------------------
    combined |      142       10153       10153

unadjusted variance    60071.92
adjustment for ties   -18458.87
                     ----------
adjusted variance      41613.05

Ho: pre~tage(psm_ro~t==0) = pre~tage(psm_ro~t==1)
             z =  -1.941
    Prob > |z| =   0.0522
```

```
. ranksum preop_asa , by(psm_robot)

Two-sample Wilcoxon rank-sum (Mann-Whitney) test

   psm_robot |      obs    rank sum    expected
-------------+---------------------------------
           0 |       71      5157.5      5076.5
           1 |       71      4995.5      5076.5
-------------+---------------------------------
    combined |      142       10153       10153

unadjusted variance    60071.92
adjustment for ties   -13961.95
                     ----------
adjusted variance      46109.97

Ho: preop_~a(psm_ro~t==0) = preop_~a(psm_ro~t==1)
             z =   0.377
    Prob > |z| =   0.7060

. ranksum preop_ecog , by(psm_robot)

Two-sample Wilcoxon rank-sum (Mann-Whitney) test

   psm_robot |      obs    rank sum    expected
-------------+---------------------------------
           0 |       71      5173.5      5076.5
           1 |       71      4979.5      5076.5
-------------+---------------------------------
    combined |      142       10153       10153

unadjusted variance    60071.92
adjustment for ties   -26524.42
                     ----------
adjusted variance      33547.50

Ho: preop~og(psm_ro~t==0) = preop~og(psm_ro~t==1)
             z =   0.530
    Prob > |z| =   0.5964

. ranksum preop_copd , by(psm_robot)

Two-sample Wilcoxon rank-sum (Mann-Whitney) test

   psm_robot |      obs    rank sum    expected
-------------+---------------------------------
           0 |       71        5041      5076.5
           1 |       71        5112      5076.5
-------------+---------------------------------
    combined |      142       10153       10153

unadjusted variance    60071.92
adjustment for ties   -53949.43
                     ----------
adjusted variance       6122.49

Ho: preop_~d(psm_ro~t==0) = preop_~d(psm_ro~t==1)
             z =  -0.454
    Prob > |z| =   0.6500

.
```
