# Supplementary material for: Robot-assisted minimally invasive esophagectomy (RAMIE) vs. hybrid minimally invasive esophagectomy: propensity score matched short-term outcome analysis of a European high-volume center
Source: Surg Endosc. 2022 May 3;36(10):7747–55. doi: 10.1007/s00464-022-09254-2 (PMC9485091; doi:10.1007/s00464-022-09254-2)
Supplement: Supplementary file 2 — Supplementary file2 (PDF 94 kb) [file 464_2022_9254_MOESM2_ESM.pdf]

```
  ___  ____  ____  ____  ____ (R)
 /__    /   ____/   /   ____/
___/   /   /___/   /   /___/   15.0   Copyright 1985-2017 StataCorp LLC
  Statistics/Data Analysis            StataCorp
                                      4905 Lakeway Drive
     Special Edition                  College Station, Texas 77845 USA
                                      800-STATA-PC        http://www.stata.com
                                      979-696-4600        stata@stata.com
                                      979-696-4601 (fax)

. logistic target_taa preop_diag

Logistic regression                             Number of obs     =        611
                                                LR chi2(1)        =       0.17
                                                Prob > chi2       =     0.6845
Log likelihood = -235.18096                     Pseudo R2         =     0.0004

------------------------------------------------------------------------------
  target_taa | Odds Ratio   Std. Err.      z    P>|z|     [95% Conf. Interval]
-------------+----------------------------------------------------------------
  preop_diag |   1.128412   .3326508     0.41   0.682     .6331947    2.010935
       _cons |   .1448598   .0196847   -14.22   0.000      .110989     .189067
------------------------------------------------------------------------------
Note: _cons estimates baseline odds.

. logistic target_taa preop_age

Logistic regression                             Number of obs     =        611
                                                LR chi2(1)        =      21.31
                                                Prob > chi2       =     0.0000
Log likelihood = -224.60664                     Pseudo R2         =     0.0453

------------------------------------------------------------------------------
  target_taa | Odds Ratio   Std. Err.      z    P>|z|     [95% Conf. Interval]
-------------+----------------------------------------------------------------
   preop_age |   1.062508   .0145247     4.44   0.000     1.034418     1.09136
       _cons |   .0028301   .0026222    -6.33   0.000     .0004604    .0173964
------------------------------------------------------------------------------
Note: _cons estimates baseline odds.

. logistic target_taa preop_sex

Logistic regression                             Number of obs     =        611
                                                LR chi2(1)        =       1.47
                                                Prob > chi2       =     0.2253
Log likelihood = -234.52829                     Pseudo R2         =     0.0031

------------------------------------------------------------------------------
  target_taa | Odds Ratio   Std. Err.      z    P>|z|     [95% Conf. Interval]
-------------+----------------------------------------------------------------
   preop_sex |   .6487013   .2418644    -1.16   0.246     .3123772    1.347132
       _cons |   .1576577   .0202748   -14.36   0.000     .1225323    .2028521
------------------------------------------------------------------------------
Note: _cons estimates baseline odds.

. logistic target_taa preop_smoke

Logistic regression                             Number of obs     =        611
                                                LR chi2(1)        =       0.06
                                                Prob > chi2       =     0.8068
Log likelihood = -235.23363                     Pseudo R2         =     0.0001

------------------------------------------------------------------------------
  target_taa | Odds Ratio   Std. Err.      z    P>|z|     [95% Conf. Interval]
-------------+----------------------------------------------------------------
 preop_smoke |   .9595532   .1620538    -0.24   0.807     .6891507    1.336054
       _cons |   .1545803   .0313329    -9.21   0.000     .1039008    .2299798
------------------------------------------------------------------------------
Note: _cons estimates baseline odds.

. logistic target_taa preop_alcohol

Logistic regression                             Number of obs     =        611
                                                LR chi2(1)        =       0.04
                                                Prob > chi2       =     0.8410
Log likelihood = -235.24341                     Pseudo R2         =     0.0001

-------------------------------------------------------------------------------
   target_taa | Odds Ratio   Std. Err.      z    P>|z|     [95% Conf. Interval]
--------------+----------------------------------------------------------------
preop_alcohol |   .9737657   .1296444    -0.20   0.842     .7501151    1.264099
        _cons |   .1509584     .02195   -13.00   0.000     .1135243    .2007362
-------------------------------------------------------------------------------
Note: _cons estimates baseline odds.

. logistic target_taa preop_khk

Logistic regression                             Number of obs     =        611
                                                LR chi2(1)        =       9.76
                                                Prob > chi2       =     0.0018
Log likelihood = -230.38437                     Pseudo R2         =     0.0207

------------------------------------------------------------------------------
  target_taa | Odds Ratio   Std. Err.      z    P>|z|     [95% Conf. Interval]
-------------+----------------------------------------------------------------
   preop_khk |   2.617394   .7651826     3.29   0.001     1.475789    4.642092
       _cons |   .1252654   .0172995   -15.04   0.000     .0955604    .1642042
------------------------------------------------------------------------------
Note: _cons estimates baseline odds.
```

```
. logistic target_taa preop_aht

Logistic regression                             Number of obs     =        611
                                                LR chi2(1)        =      11.03
                                                Prob > chi2       =     0.0009
Log likelihood = -229.75097                     Pseudo R2         =     0.0234

------------------------------------------------------------------------------
  target_taa | Odds Ratio   Std. Err.      z    P>|z|     [95% Conf. Interval]
-------------+----------------------------------------------------------------
   preop_aht |   2.331818   .6193078     3.19   0.001     1.385556    3.924329
       _cons |   .0873016   .0194082   -10.97   0.000     .0564663    .1349755
------------------------------------------------------------------------------
Note: _cons estimates baseline odds.

. logistic target_taa preop_vhf

Logistic regression                             Number of obs     =        611
                                                LR chi2(1)        =      14.79
                                                Prob > chi2       =     0.0001
Log likelihood = -227.86757                     Pseudo R2         =     0.0314

------------------------------------------------------------------------------
  target_taa | Odds Ratio   Std. Err.      z    P>|z|     [95% Conf. Interval]
-------------+----------------------------------------------------------------
   preop_vhf |   3.968254   1.325795     4.13   0.000      2.06164    7.638114
       _cons |       .126   .0168449   -15.49   0.000     .0969557    .1637449
------------------------------------------------------------------------------
Note: _cons estimates baseline odds.

. logistic target_taa preop_mi

Logistic regression                             Number of obs     =        611
                                                LR chi2(1)        =       3.69
                                                Prob > chi2       =     0.0548
Log likelihood = -233.41904                     Pseudo R2         =     0.0078

------------------------------------------------------------------------------
  target_taa | Odds Ratio   Std. Err.      z    P>|z|     [95% Conf. Interval]
-------------+----------------------------------------------------------------
    preop_mi |   2.102941   .7696738     2.03   0.042     1.026333    4.308894
       _cons |   .1376518   .0178046   -15.33   0.000     .1068275    .1773702
------------------------------------------------------------------------------
Note: _cons estimates baseline odds.

. logistic target_taa preop_revasc

Logistic regression                             Number of obs     =        611
                                                LR chi2(1)        =       1.35
                                                Prob > chi2       =     0.2455
Log likelihood = -234.58919                     Pseudo R2         =     0.0029

------------------------------------------------------------------------------
  target_taa | Odds Ratio   Std. Err.      z    P>|z|     [95% Conf. Interval]
-------------+----------------------------------------------------------------
preop_revasc |   1.650375   .6814753     1.21   0.225     .7346884    3.707337
       _cons |   .1425703   .0180859   -15.36   0.000     .1111856     .182814
------------------------------------------------------------------------------
Note: _cons estimates baseline odds.

. logistic target_taa preop_pavk

Logistic regression                             Number of obs     =        611
                                                LR chi2(1)        =       0.01
                                                Prob > chi2       =     0.9204
Log likelihood = -235.25854                     Pseudo R2         =     0.0000

------------------------------------------------------------------------------
  target_taa | Odds Ratio   Std. Err.      z    P>|z|     [95% Conf. Interval]
-------------+----------------------------------------------------------------
  preop_pavk |    1.06579   .6749675     0.10   0.920     .3080376    3.687565
       _cons |   .1481481   .0182091   -15.54   0.000     .1164324    .1885031
------------------------------------------------------------------------------
Note: _cons estimates baseline odds.

. logistic target_taa preop_copd

Logistic regression                             Number of obs     =        611
                                                LR chi2(1)        =       1.29
                                                Prob > chi2       =     0.2568
Log likelihood = -234.62047                     Pseudo R2         =     0.0027

------------------------------------------------------------------------------
  target_taa | Odds Ratio   Std. Err.      z    P>|z|     [95% Conf. Interval]
-------------+----------------------------------------------------------------
  preop_copd |    .638036   .2778031    -1.03   0.302     .2717872    1.497826
       _cons |   .1541846    .019139   -15.06   0.000     .1208874    .1966531
------------------------------------------------------------------------------
Note: _cons estimates baseline odds.

. logistic target_taa preop_liver

Logistic regression                             Number of obs     =        611
                                                LR chi2(1)        =       0.59
                                                Prob > chi2       =     0.4408
Log likelihood = -234.96641                     Pseudo R2         =     0.0013

------------------------------------------------------------------------------
  target_taa | Odds Ratio   Std. Err.      z    P>|z|     [95% Conf. Interval]
-------------+----------------------------------------------------------------
 preop_liver |   .6379457   .3936416    -0.73   0.466     .1903503     2.13803
```

```
       _cons |   .1516966    .018674   -15.32   0.000     .1191769      .19309
------------------------------------------------------------------------------
Note: _cons estimates baseline odds.

. logistic target_taa preop_crea_tot

Logistic regression                             Number of obs     =        611
                                                LR chi2(1)        =       6.43
                                                Prob > chi2       =     0.0112
Log likelihood = -232.04856                     Pseudo R2         =     0.0137

--------------------------------------------------------------------------------
    target_taa | Odds Ratio   Std. Err.      z    P>|z|     [95% Conf. Interval]
---------------+----------------------------------------------------------------
preop_crea_tot |   3.706033   1.901321     2.55   0.011     1.355857    10.12989
         _cons |   .0457193    .022274    -6.33   0.000     .0175955    .1187949
--------------------------------------------------------------------------------
Note: _cons estimates baseline odds.

. logistic target_taa preop_gfr_tot

Logistic regression                             Number of obs     =        611
                                                LR chi2(1)        =      16.41
                                                Prob > chi2       =     0.0001
Log likelihood = -227.05897                     Pseudo R2         =     0.0349

-------------------------------------------------------------------------------
   target_taa | Odds Ratio   Std. Err.      z    P>|z|     [95% Conf. Interval]
--------------+----------------------------------------------------------------
preop_gfr_tot |   .9730203   .0066902    -3.98   0.000     .9599957    .9862216
        _cons |   1.433115   .8056801     0.64   0.522     .4761523    4.313367
-------------------------------------------------------------------------------
Note: _cons estimates baseline odds.

. logistic target_taa preop_quick_tot

Logistic regression                             Number of obs     =        611
                                                LR chi2(1)        =       7.51
                                                Prob > chi2       =     0.0061
Log likelihood = -231.50712                     Pseudo R2         =     0.0160

---------------------------------------------------------------------------------
     target_taa | Odds Ratio   Std. Err.      z    P>|z|     [95% Conf. Interval]
----------------+----------------------------------------------------------------
preop_quick_tot |   .9800072   .0069898    -2.83   0.005     .9664028     .993803
          _cons |   1.243018   .9298173     0.29   0.771      .286914    5.385219
---------------------------------------------------------------------------------
Note: _cons estimates baseline odds.

. logistic target_taa preop_albumin_tot

Logistic regression                             Number of obs     =        611
                                                LR chi2(1)        =       0.01
                                                Prob > chi2       =     0.9209
Log likelihood =  -235.2586                     Pseudo R2         =     0.0000

-----------------------------------------------------------------------------------
       target_taa | Odds Ratio   Std. Err.      z    P>|z|     [95% Conf. Interval]
------------------+----------------------------------------------------------------
preop_albumin_tot |   1.002858   .0288767     0.10   0.921      .947828    1.061083
            _cons |   .1324903   .1533705    -1.75   0.081     .0137036    1.280953
-----------------------------------------------------------------------------------
Note: _cons estimates baseline odds.

. logistic target_taa preop_biliribin_tot

Logistic regression                             Number of obs     =        611
                                                LR chi2(1)        =       0.00
                                                Prob > chi2       =     0.9672
Log likelihood = -235.26268                     Pseudo R2         =     0.0000

-------------------------------------------------------------------------------------
         target_taa | Odds Ratio   Std. Err.      z    P>|z|     [95% Conf. Interval]
--------------------+----------------------------------------------------------------
preop_biliribin_tot |   .9781445   .5262033    -0.04   0.967     .3407925    2.807476
              _cons |   .1499588   .0400588    -7.10   0.000     .0888359    .2531368
-------------------------------------------------------------------------------------
Note: _cons estimates baseline odds.

. logistic target_taa preop_platelet_bin

Logistic regression                             Number of obs     =        611
                                                LR chi2(1)        =       0.28
                                                Prob > chi2       =     0.5974
Log likelihood =  -235.1241                     Pseudo R2         =     0.0006

------------------------------------------------------------------------------------
        target_taa | Odds Ratio   Std. Err.      z    P>|z|     [95% Conf. Interval]
-------------------+----------------------------------------------------------------
preop_platelet_bin |   1.284247     .59356     0.54   0.588     .5190815    3.177323
             _cons |       .146   .0182929   -15.36   0.000     .1142095    .1866395
------------------------------------------------------------------------------------
Note: _cons estimates baseline odds.

. logistic target_taa preop_platelet_tot

Logistic regression                             Number of obs     =        611
                                                LR chi2(1)        =       0.09
                                                Prob > chi2       =     0.7673
Log likelihood = -235.21976                     Pseudo R2         =     0.0002
```

```
------------------------------------------------------------------------------------
        target_taa | Odds Ratio   Std. Err.      z    P>|z|     [95% Conf. Interval]
-------------------+----------------------------------------------------------------
preop_platelet_tot |   .9995634   .0014865    -0.29   0.769     .9966541    1.002481
             _cons |   .1650721   .0624743    -4.76   0.000      .078618    .3465972
------------------------------------------------------------------------------------
Note: _cons estimates baseline odds.

. logistic target_taa preop_leuko_bin

Logistic regression                             Number of obs     =        611
                                                LR chi2(1)        =       0.02
                                                Prob > chi2       =     0.8970
Log likelihood = -235.25514                     Pseudo R2         =     0.0000

---------------------------------------------------------------------------------
     target_taa | Odds Ratio   Std. Err.      z    P>|z|     [95% Conf. Interval]
----------------+----------------------------------------------------------------
preop_leuko_bin |   1.050739   .3997616     0.13   0.896     .4984813    2.214833
          _cons |   .1476793   .0189095   -14.94   0.000     .1149021    .1898067
---------------------------------------------------------------------------------
Note: _cons estimates baseline odds.

. logistic target_taa preop_leuko_tot

Logistic regression                             Number of obs     =        611
                                                LR chi2(1)        =       3.95
                                                Prob > chi2       =     0.0468
Log likelihood = -233.28696                     Pseudo R2         =     0.0084

---------------------------------------------------------------------------------
     target_taa | Odds Ratio   Std. Err.      z    P>|z|     [95% Conf. Interval]
----------------+----------------------------------------------------------------
preop_leuko_tot |   1.093553   .0476595     2.05   0.040      1.00402    1.191069
          _cons |   .0826202   .0263416    -7.82   0.000     .0442283    .1543379
---------------------------------------------------------------------------------
Note: _cons estimates baseline odds.

. logistic target_taa preop_diabetes

Logistic regression                             Number of obs     =        611
                                                LR chi2(1)        =       3.60
                                                Prob > chi2       =     0.0579
Log likelihood =  -233.4657                     Pseudo R2         =     0.0076

--------------------------------------------------------------------------------
    target_taa | Odds Ratio   Std. Err.      z    P>|z|     [95% Conf. Interval]
---------------+----------------------------------------------------------------
preop_diabetes |   1.878972   .5981953     1.98   0.048     1.006768    3.506804
         _cons |   .1353066   .0180213   -15.02   0.000     .1042194    .1756665
--------------------------------------------------------------------------------
Note: _cons estimates baseline odds.

. logistic target_taa preop_weight

Logistic regression                             Number of obs     =        611
                                                LR chi2(1)        =       0.84
                                                Prob > chi2       =     0.3584
Log likelihood = -234.84181                     Pseudo R2         =     0.0018

------------------------------------------------------------------------------
  target_taa | Odds Ratio   Std. Err.      z    P>|z|     [95% Conf. Interval]
-------------+----------------------------------------------------------------
preop_weight |   1.006116   .0066377     0.92   0.355     .9931905    1.019211
       _cons |   .0886003   .0510642    -4.21   0.000     .0286319    .2741701
------------------------------------------------------------------------------
Note: _cons estimates baseline odds.

. logistic target_taa preop_height

Logistic regression                             Number of obs     =        611
                                                LR chi2(1)        =       2.58
                                                Prob > chi2       =     0.1084
Log likelihood = -233.97515                     Pseudo R2         =     0.0055

------------------------------------------------------------------------------
  target_taa | Odds Ratio   Std. Err.      z    P>|z|     [95% Conf. Interval]
-------------+----------------------------------------------------------------
preop_height |   9.688909   13.77749     1.60   0.110     .5968681    157.2792
       _cons |   .0026341   .0066807    -2.34   0.019     .0000183    .3797143
------------------------------------------------------------------------------
Note: _cons estimates baseline odds.

. logistic target_taa preop_bmi

Logistic regression                             Number of obs     =        611
                                                LR chi2(1)        =       0.04
                                                Prob > chi2       =     0.8470
Log likelihood = -235.24492                     Pseudo R2         =     0.0001

------------------------------------------------------------------------------
  target_taa | Odds Ratio   Std. Err.      z    P>|z|     [95% Conf. Interval]
-------------+----------------------------------------------------------------
   preop_bmi |   1.004787   .0248219     0.19   0.847     .9572962    1.054634
       _cons |    .130691   .0879041    -3.03   0.002     .0349719    .4883965
------------------------------------------------------------------------------
Note: _cons estimates baseline odds.

. logistic target_taa preop_ecog

Logistic regression                             Number of obs     =        611
```

```
                                                LR chi2(1)        =       3.75
                                                Prob > chi2       =     0.0529
Log likelihood = -233.39055                     Pseudo R2         =     0.0080

------------------------------------------------------------------------------
  target_taa | Odds Ratio   Std. Err.      z    P>|z|     [95% Conf. Interval]
-------------+----------------------------------------------------------------
  preop_ecog |   1.368788   .2163941     1.99   0.047      1.00408    1.865968
       _cons |   .1231826   .0196146   -13.15   0.000     .0901594    .1683015
------------------------------------------------------------------------------
Note: _cons estimates baseline odds.

. logistic target_taa preop_tstage

Logistic regression                             Number of obs     =        611
                                                LR chi2(1)        =       0.11
                                                Prob > chi2       =     0.7367
Log likelihood =   -235.207                     Pseudo R2         =     0.0002

------------------------------------------------------------------------------
  target_taa | Odds Ratio   Std. Err.      z    P>|z|     [95% Conf. Interval]
-------------+----------------------------------------------------------------
preop_tstage |   1.076618   .2392557     0.33   0.740     .6964662    1.664268
       _cons |    .120774   .0767448    -3.33   0.001     .0347604    .4196255
------------------------------------------------------------------------------
Note: _cons estimates baseline odds.

. logistic target_taa preop_neoadj

Logistic regression                             Number of obs     =        611
                                                LR chi2(1)        =       1.31
                                                Prob > chi2       =     0.2517
Log likelihood = -234.60652                     Pseudo R2         =     0.0028

------------------------------------------------------------------------------
  target_taa | Odds Ratio   Std. Err.      z    P>|z|     [95% Conf. Interval]
-------------+----------------------------------------------------------------
preop_neoadj |   .8382181   .1277898    -1.16   0.247     .6217101    1.130124
       _cons |   .1912633   .0467677    -6.76   0.000     .1184393     .308864
------------------------------------------------------------------------------
Note: _cons estimates baseline odds.

. logistic target_taa preop_asa

Logistic regression                             Number of obs     =        611
                                                LR chi2(1)        =       0.02
                                                Prob > chi2       =     0.8804
Log likelihood = -235.25221                     Pseudo R2         =     0.0000

------------------------------------------------------------------------------
  target_taa | Odds Ratio   Std. Err.      z    P>|z|     [95% Conf. Interval]
-------------+----------------------------------------------------------------
   preop_asa |   .9747713   .1655762    -0.15   0.880     .6987411    1.359844
       _cons |   .1534527   .0381545    -7.54   0.000     .0942609    .2498144
------------------------------------------------------------------------------
Note: _cons estimates baseline odds.

. logistic target_taa preop_weightloss_bin

Logistic regression                             Number of obs     =        611
                                                LR chi2(1)        =       0.47
                                                Prob > chi2       =     0.4947
Log likelihood = -235.03033                     Pseudo R2         =     0.0010

--------------------------------------------------------------------------------------
          target_taa | Odds Ratio   Std. Err.      z    P>|z|     [95% Conf. Interval]
---------------------+----------------------------------------------------------------
preop_weightloss_bin |   .8183422   .2438228    -0.67   0.501     .4563749    1.467399
               _cons |   .1551724    .021012   -13.76   0.000     .1190016    .2023375
--------------------------------------------------------------------------------------
Note: _cons estimates baseline odds.

. logistic target_taa preop_fev1_bin

Logistic regression                             Number of obs     =        611
                                                LR chi2(1)        =       0.03
                                                Prob > chi2       =     0.8651
Log likelihood = -235.24909                     Pseudo R2         =     0.0001

--------------------------------------------------------------------------------
    target_taa | Odds Ratio   Std. Err.      z    P>|z|     [95% Conf. Interval]
---------------+----------------------------------------------------------------
preop_fev1_bin |    .951613    .278706    -0.17   0.866     .5359973      1.6895
         _cons |   .1501211   .0204464   -13.92   0.000     .1149498    .1960537
--------------------------------------------------------------------------------
Note: _cons estimates baseline odds.

. logistic target_taa preop_fev1_tot

Logistic regression                             Number of obs     =        611
                                                LR chi2(1)        =       2.65
                                                Prob > chi2       =     0.1037
Log likelihood = -233.93936                     Pseudo R2         =     0.0056

--------------------------------------------------------------------------------
    target_taa | Odds Ratio   Std. Err.      z    P>|z|     [95% Conf. Interval]
---------------+----------------------------------------------------------------
preop_fev1_tot |   .7745804   .1226185    -1.61   0.107     .5679611    1.056366
         _cons |   .3204551   .1548371    -2.36   0.019     .1243039    .8261323
--------------------------------------------------------------------------------
Note: _cons estimates baseline odds.
```

```
. logistic target_taa preop_vc_bin

Logistic regression                             Number of obs     =        611
                                                LR chi2(1)        =       2.35
                                                Prob > chi2       =     0.1256
Log likelihood = -234.09039                     Pseudo R2         =     0.0050

------------------------------------------------------------------------------
  target_taa | Odds Ratio   Std. Err.      z    P>|z|     [95% Conf. Interval]
-------------+----------------------------------------------------------------
preop_vc_bin |   1.559322   .4403914     1.57   0.116     .8964693    2.712291
       _cons |   .1350114    .018726   -14.44   0.000     .1028749    .1771869
------------------------------------------------------------------------------
Note: _cons estimates baseline odds.

. logistic target_taa preop_vc_tot

Logistic regression                             Number of obs     =        611
                                                LR chi2(1)        =       1.50
                                                Prob > chi2       =     0.2209
Log likelihood = -234.51423                     Pseudo R2         =     0.0032

------------------------------------------------------------------------------
  target_taa | Odds Ratio   Std. Err.      z    P>|z|     [95% Conf. Interval]
-------------+----------------------------------------------------------------
preop_vc_tot |   .8529321   .1111919    -1.22   0.222     .6606146    1.101237
       _cons |    .274819   .1407824    -2.52   0.012      .100693    .7500572
------------------------------------------------------------------------------
Note: _cons estimates baseline odds.

. logistic target_taa psm_robot

Logistic regression                             Number of obs     =        611
                                                LR chi2(1)        =       0.09
                                                Prob > chi2       =     0.7602
Log likelihood = -235.21696                     Pseudo R2         =     0.0002

------------------------------------------------------------------------------
  target_taa | Odds Ratio   Std. Err.      z    P>|z|     [95% Conf. Interval]
-------------+----------------------------------------------------------------
   psm_robot |   .8923241   .3368127    -0.30   0.763     .4258257    1.869879
       _cons |   .1505376   .0192995   -14.77   0.000     .1170894    .1935408
------------------------------------------------------------------------------
Note: _cons estimates baseline odds.

.
. logistic target_taa preop_diag preop_age preop_sex preop_smoke preop_alcohol preop_khk preop_aht preop_vhf preop_mi
preop_revasc preop_pavk preop_copd preop_liver preop_crea_tot
>  preop_gfr_tot preop_quick_tot preop_albumin_tot preop_biliribin_tot preop_platelet_bin preop_platelet_tot preop_leuko_bin
preop_leuko_tot preop_diabetes preop_weight preop_heig
> ht preop_bmi preop_ecog preop_tstage preop_neoadj preop_asa preop_weightloss_bin preop_fev1_bin preop_fev1_tot
preop_vc_bin preop_vc_tot psm_robot

Logistic regression                             Number of obs     =        611
                                                LR chi2(36)       =      69.28
                                                Prob > chi2       =     0.0007
Log likelihood = -200.62342                     Pseudo R2         =     0.1472

--------------------------------------------------------------------------------------
          target_taa | Odds Ratio   Std. Err.      z    P>|z|     [95% Conf. Interval]
---------------------+----------------------------------------------------------------
          preop_diag |   1.212468   .4504613     0.52   0.604     .5853668    2.511379
           preop_age |   1.029272    .025438     1.17   0.243     .9806033    1.080357
           preop_sex |   .6157468   .3764063    -0.79   0.428     .1858071    2.040526
         preop_smoke |   1.243441   .2663737     1.02   0.309     .8171052    1.892222
       preop_alcohol |   .9473085    .148236    -0.35   0.729     .6970988    1.287326
           preop_khk |   2.930509   1.413912     2.23   0.026     1.138297    7.544503
           preop_aht |   1.492541   .4576605     1.31   0.192     .8183158    2.722274
           preop_vhf |    1.81805     .75713     1.44   0.151     .8037509    4.112351
            preop_mi |   1.142326   .6500726     0.23   0.815     .3744464    3.484899
        preop_revasc |   .3333396   .2136388    -1.71   0.087     .0949179    1.170646
          preop_pavk |   .4052242   .3054319    -1.20   0.231     .0924942    1.775319
          preop_copd |    .466229   .2383133    -1.49   0.135     .1712013    1.269672
         preop_liver |   .8653545   .5740192    -0.22   0.827     .2358105    3.175595
      preop_crea_tot |   .3843441   .4853329    -0.76   0.449     .0323489    4.566467
       preop_gfr_tot |   .9764239   .0171285    -1.36   0.174     .9434232    1.010579
     preop_quick_tot |   .9893801   .0087404    -1.21   0.227     .9723967     1.00666
   preop_albumin_tot |   1.057479   .0383321     1.54   0.123     .9849557    1.135341
 preop_biliribin_tot |   .4025788   .2644462    -1.39   0.166     .1110998    1.458776
  preop_platelet_bin |   1.220499   .6698717     0.36   0.717     .4162503    3.578656
  preop_platelet_tot |   .9987144   .0017791    -0.72   0.470     .9952335    1.002207
     preop_leuko_bin |   1.488119   .6534005     0.91   0.365     .6293525    3.518695
     preop_leuko_tot |   1.176902   .0679721     2.82   0.005     1.050943    1.317958
      preop_diabetes |   1.518711   .5537135     1.15   0.252     .7432444    3.103262
        preop_weight |   1.069186   .1026121     0.70   0.486     .8858527    1.290461
        preop_height |   .2339828    2.23999    -0.15   0.879     1.66e-09    3.30e+07
           preop_bmi |   .8020703   .2455157    -0.72   0.471     .4402076    1.461394
          preop_ecog |   1.247289   .2364959     1.17   0.244     .8601463     1.80868
        preop_tstage |   1.269842   .3545417     0.86   0.392     .7346714    2.194858
        preop_neoadj |    1.18925    .252531     0.82   0.414     .7843769    1.803106
           preop_asa |   .7364878   .1604385    -1.40   0.160     .4805489    1.128739
preop_weightloss_bin |   .8502397   .2841309    -0.49   0.627      .441661    1.636793
      preop_fev1_bin |   .4554011   .2264932    -1.58   0.114     .1718106    1.207086
      preop_fev1_tot |   .6347472   .2685108    -1.07   0.283     .2770292    1.454374
        preop_vc_bin |   1.984129   .9041616     1.50   0.133     .8122318    4.846854
        preop_vc_tot |   1.019245   .3342744     0.06   0.954     .5359399    1.938389
           psm_robot |    1.53985   .7173701     0.93   0.354     .6179158    3.837318
               _cons |   1.526897   26.69758     0.02   0.981     2.00e-15    1.17e+15
--------------------------------------------------------------------------------------
Note: _cons estimates baseline odds.
```

```
. logistic target_taa preop_khk preop_leuko_tot

Logistic regression                             Number of obs     =        611
                                                LR chi2(2)        =      12.92
                                                Prob > chi2       =     0.0016
Log likelihood = -228.80571                     Pseudo R2         =     0.0274

--------------------------------------------------------------------------------
     target_taa | Odds Ratio   Std. Err.      z    P>|z|     [95% Conf. Interval]
---------------+----------------------------------------------------------------
      preop_khk |    2.52477   .7432057     3.15   0.002     1.417939    4.495587
preop_leuko_tot |   1.083835   .0478323     1.82   0.068     .9940257    1.181759
          _cons |    .074406   .0241831    -7.99   0.000     .0393509    .1406894
--------------------------------------------------------------------------------
Note: _cons estimates baseline odds.

.
```

```
  ___  ____  ____  ____  ____ (R)
 /__    /   ____/   /   ____/
___/   /   /___/   /   /___/   15.0   Copyright 1985-2017 StataCorp LLC
  Statistics/Data Analysis            StataCorp
                                      4905 Lakeway Drive
     Special Edition                  College Station, Texas 77845 USA
                                      800-STATA-PC        http://www.stata.com
                                      979-696-4600        stata@stata.com
                                      979-696-4601 (fax)

. logistic target_pneumonia preop_diag

Logistic regression                             Number of obs     =        611
                                                LR chi2(1)        =       3.02
                                                Prob > chi2       =     0.0823
Log likelihood = -220.03814                     Pseudo R2         =     0.0068

---------------------------------------------------------------------------------
target_pneumonia | Odds Ratio   Std. Err.      z    P>|z|     [95% Conf. Interval]
----------------+----------------------------------------------------------------
      preop_diag |   .5446429   .2025124    -1.63   0.102     .2627925    1.128784
           _cons |    .147541   .0199125   -14.18   0.000     .1132484    .1922176
---------------------------------------------------------------------------------
Note: _cons estimates baseline odds.

. logistic target_pneumonia preop_age

Logistic regression                             Number of obs     =        611
                                                LR chi2(1)        =       4.04
                                                Prob > chi2       =     0.0444
Log likelihood =  -219.5272                     Pseudo R2         =     0.0091

---------------------------------------------------------------------------------
target_pneumonia | Odds Ratio   Std. Err.      z    P>|z|     [95% Conf. Interval]
----------------+----------------------------------------------------------------
       preop_age |   1.026848   .0136856     1.99   0.047     1.000372    1.054025
           _cons |   .0243534   .0213705    -4.23   0.000     .0043613    .1359893
---------------------------------------------------------------------------------
Note: _cons estimates baseline odds.

. logistic target_pneumonia preop_sex

Logistic regression                             Number of obs     =        611
                                                LR chi2(1)        =       0.25
                                                Prob > chi2       =     0.6179
Log likelihood = -221.42319                     Pseudo R2         =     0.0006

---------------------------------------------------------------------------------
target_pneumonia | Odds Ratio   Std. Err.      z    P>|z|     [95% Conf. Interval]
----------------+----------------------------------------------------------------
       preop_sex |   .8379681   .3019427    -0.49   0.624      .413539    1.698003
           _cons |   .1371681   .0185768   -14.67   0.000       .10519    .1788677
---------------------------------------------------------------------------------
Note: _cons estimates baseline odds.

. logistic target_pneumonia preop_smoke

Logistic regression                             Number of obs     =        611
                                                LR chi2(1)        =       0.21
                                                Prob > chi2       =     0.6495
Log likelihood = -221.44435                     Pseudo R2         =     0.0005

---------------------------------------------------------------------------------
target_pneumonia | Odds Ratio   Std. Err.      z    P>|z|     [95% Conf. Interval]
----------------+----------------------------------------------------------------
     preop_smoke |   .9232233   .1624066    -0.45   0.650     .6539879    1.303298
           _cons |   .1442831   .0301385    -9.27   0.000     .0958105    .2172791
---------------------------------------------------------------------------------
Note: _cons estimates baseline odds.

. logistic target_pneumonia preop_alcohol

Logistic regression                             Number of obs     =        611
                                                LR chi2(1)        =       0.73
                                                Prob > chi2       =     0.3918
Log likelihood = -221.18097                     Pseudo R2         =     0.0017

---------------------------------------------------------------------------------
```

```
target_pneumonia | Odds Ratio   Std. Err.      z    P>|z|     [95% Conf. Interval]
-----------------+----------------------------------------------------------------
   preop_alcohol |   .8848654   .1293992    -0.84   0.403      .664356    1.178565
           _cons |   .1435524   .0214208   -13.01   0.000     .1071506    .1923209
----------------------------------------------------------------------------------
Note: _cons estimates baseline odds.

. logistic target_pneumonia preop_khk

Logistic regression                             Number of obs     =        611
                                                LR chi2(1)        =       3.63
                                                Prob > chi2       =     0.0567
Log likelihood = -219.73119                     Pseudo R2         =     0.0082

----------------------------------------------------------------------------------
target_pneumonia | Odds Ratio   Std. Err.      z    P>|z|     [95% Conf. Interval]
-----------------+----------------------------------------------------------------
       preop_khk |   1.885964   .6007801     1.99   0.046     1.010137    3.521166
           _cons |   .1205074    .016896   -15.09   0.000     .0915523    .1586201
----------------------------------------------------------------------------------
Note: _cons estimates baseline odds.

. logistic target_pneumonia preop_aht

Logistic regression                             Number of obs     =        611
                                                LR chi2(1)        =       2.56
                                                Prob > chi2       =     0.1099
Log likelihood = -220.26963                     Pseudo R2         =     0.0058

----------------------------------------------------------------------------------
target_pneumonia | Odds Ratio   Std. Err.      z    P>|z|     [95% Conf. Interval]
-----------------+----------------------------------------------------------------
       preop_aht |   1.507798   .3922285     1.58   0.114     .9055601    2.510551
           _cons |   .1048387   .0216115   -10.94   0.000      .069993    .1570321
----------------------------------------------------------------------------------
Note: _cons estimates baseline odds.

. logistic target_pneumonia preop_vhf

Logistic regression                             Number of obs     =        611
                                                LR chi2(1)        =       0.03
                                                Prob > chi2       =     0.8736
Log likelihood = -221.53494                     Pseudo R2         =     0.0001

----------------------------------------------------------------------------------
target_pneumonia | Odds Ratio   Std. Err.      z    P>|z|     [95% Conf. Interval]
-----------------+----------------------------------------------------------------
       preop_vhf |   1.075759   .4901963     0.16   0.873     .4403964    2.627762
           _cons |   .1327968   .0173977   -15.41   0.000      .102724    .1716735
----------------------------------------------------------------------------------
Note: _cons estimates baseline odds.

. logistic target_pneumonia preop_mi

Logistic regression                             Number of obs     =        611
                                                LR chi2(1)        =       1.96
                                                Prob > chi2       =     0.1610
Log likelihood = -220.56511                     Pseudo R2         =     0.0044

----------------------------------------------------------------------------------
target_pneumonia | Odds Ratio   Std. Err.      z    P>|z|     [95% Conf. Interval]
-----------------+----------------------------------------------------------------
        preop_mi |   1.782142   .6993361     1.47   0.141     .8258848    3.845608
           _cons |   .1262525   .0168806   -15.48   0.000     .0971472    .1640778
----------------------------------------------------------------------------------
Note: _cons estimates baseline odds.

. logistic target_pneumonia preop_revasc

Logistic regression                             Number of obs     =        611
                                                LR chi2(1)        =       0.00
                                                Prob > chi2       =     0.9800
Log likelihood = -221.54727                     Pseudo R2         =     0.0000

----------------------------------------------------------------------------------
target_pneumonia | Odds Ratio   Std. Err.      z    P>|z|     [95% Conf. Interval]
-----------------+----------------------------------------------------------------
    preop_revasc |   1.012505   .5000836     0.03   0.980     .3845763    2.665704
           _cons |   .1334661   .0173595   -15.48   0.000     .1034327    .1722203
----------------------------------------------------------------------------------
Note: _cons estimates baseline odds.

. logistic target_pneumonia preop_pavk

Logistic regression                             Number of obs     =        611
                                                LR chi2(1)        =       2.16
                                                Prob > chi2       =     0.1416
Log likelihood = -220.46725                     Pseudo R2         =     0.0049

----------------------------------------------------------------------------------
target_pneumonia | Odds Ratio   Std. Err.      z    P>|z|     [95% Conf. Interval]
-----------------+----------------------------------------------------------------
      preop_pavk |   2.291484   1.203115     1.58   0.114     .8188491    6.412534
           _cons |   .1283525   .0166567   -15.82   0.000     .0995271    .1655265
----------------------------------------------------------------------------------
Note: _cons estimates baseline odds.

. logistic target_pneumonia preop_copd

Logistic regression                             Number of obs     =        611
                                                LR chi2(1)        =       3.03
```

```
                                                Prob > chi2       =     0.0816
Log likelihood = -220.03128                     Pseudo R2         =     0.0068

------------------------------------------------------------------------------------
target_pneumonia | Odds Ratio   Std. Err.      z    P>|z|     [95% Conf. Interval]
-----------------+------------------------------------------------------------------
      preop_copd |   1.652755   .4460945     1.86   0.063     .9737804    2.805149
           _cons |   .1250924   .0166341   -15.63   0.000     .0963925    .1623375
------------------------------------------------------------------------------------
Note: _cons estimates baseline odds.

. logistic target_pneumonia preop_liver

Logistic regression                             Number of obs     =        611
                                                LR chi2(1)        =       0.33
                                                Prob > chi2       =     0.5672
Log likelihood = -221.38388                     Pseudo R2         =     0.0007

------------------------------------------------------------------------------------
target_pneumonia | Odds Ratio   Std. Err.      z    P>|z|     [95% Conf. Interval]
-----------------+------------------------------------------------------------------
     preop_liver |   .7124825   .4403883    -0.55   0.583       .21215    2.392794
           _cons |   .1358268   .0174268   -15.56   0.000      .105627    .1746609
------------------------------------------------------------------------------------
Note: _cons estimates baseline odds.

. logistic target_pneumonia preop_crea_tot

Logistic regression                             Number of obs     =        611
                                                LR chi2(1)        =       0.21
                                                Prob > chi2       =     0.6487
Log likelihood = -221.44385                     Pseudo R2         =     0.0005

------------------------------------------------------------------------------------
target_pneumonia | Odds Ratio   Std. Err.      z    P>|z|     [95% Conf. Interval]
-----------------+------------------------------------------------------------------
  preop_crea_tot |    1.29684   .7314277     0.46   0.645     .4293409    3.917153
           _cons |   .1062236   .0547478    -4.35   0.000     .0386822    .2916967
------------------------------------------------------------------------------------
Note: _cons estimates baseline odds.

. logistic target_pneumonia preop_gfr_tot

Logistic regression                             Number of obs     =        611
                                                LR chi2(1)        =       1.39
                                                Prob > chi2       =     0.2381
Log likelihood = -220.85163                     Pseudo R2         =     0.0031

------------------------------------------------------------------------------------
target_pneumonia | Odds Ratio   Std. Err.      z    P>|z|     [95% Conf. Interval]
-----------------+------------------------------------------------------------------
   preop_gfr_tot |    .991886   .0068627    -1.18   0.239     .9785262    1.005428
           _cons |   .2672543   .1590871    -2.22   0.027     .0832213    .8582523
------------------------------------------------------------------------------------
Note: _cons estimates baseline odds.

. logistic target_pneumonia preop_quick_tot

Logistic regression                             Number of obs     =        611
                                                LR chi2(1)        =       0.23
                                                Prob > chi2       =     0.6305
Log likelihood = -221.43188                     Pseudo R2         =     0.0005

------------------------------------------------------------------------------------
target_pneumonia | Odds Ratio   Std. Err.      z    P>|z|     [95% Conf. Interval]
-----------------+------------------------------------------------------------------
 preop_quick_tot |   .9960469   .0081143    -0.49   0.627     .9802695    1.012078
           _cons |    .203753    .178199    -1.82   0.069     .0366993     1.13123
------------------------------------------------------------------------------------
Note: _cons estimates baseline odds.

. logistic target_pneumonia preop_albumin_tot

Logistic regression                             Number of obs     =        611
                                                LR chi2(1)        =       0.04
                                                Prob > chi2       =     0.8378
Log likelihood = -221.52664                     Pseudo R2         =     0.0001

-------------------------------------------------------------------------------------
 target_pneumonia | Odds Ratio   Std. Err.      z    P>|z|     [95% Conf. Interval]
------------------+------------------------------------------------------------------
preop_albumin_tot |   1.006164   .0302955     0.20   0.838     .9485044    1.067329
            _cons |   .1044771   .1266027    -1.86   0.062     .0097175    1.123275
-------------------------------------------------------------------------------------
Note: _cons estimates baseline odds.

. logistic target_pneumonia preop_biliribin_tot

Logistic regression                             Number of obs     =        611
                                                LR chi2(1)        =       2.30
                                                Prob > chi2       =     0.1292
Log likelihood = -220.39684                     Pseudo R2         =     0.0052

---------------------------------------------------------------------------------------
   target_pneumonia | Odds Ratio   Std. Err.      z    P>|z|     [95% Conf. Interval]
--------------------+------------------------------------------------------------------
preop_biliribin_tot |    2.14787   1.040168     1.58   0.114     .8313596    5.549156
              _cons |    .094019   .0246498    -9.02   0.000     .0562404    .1571748
---------------------------------------------------------------------------------------
Note: _cons estimates baseline odds.
```

```
. logistic target_pneumonia preop_platelet_bin

Logistic regression                             Number of obs     =        611
                                                LR chi2(1)        =       2.01
                                                Prob > chi2       =     0.1560
Log likelihood = -220.54129                     Pseudo R2         =     0.0045

------------------------------------------------------------------------------------
  target_pneumonia | Odds Ratio   Std. Err.      z    P>|z|     [95% Conf. Interval]
-------------------+----------------------------------------------------------------
preop_platelet_bin |   .3992063    .294454    -1.24   0.213     .0940486    1.694503
             _cons |    .139165   .0177531   -15.46   0.000     .1083786    .1786968
------------------------------------------------------------------------------------
Note: _cons estimates baseline odds.

. logistic target_pneumonia preop_platelet_tot

Logistic regression                             Number of obs     =        611
                                                LR chi2(1)        =       0.44
                                                Prob > chi2       =     0.5080
Log likelihood = -221.32852                     Pseudo R2         =     0.0010

------------------------------------------------------------------------------------
  target_pneumonia | Odds Ratio   Std. Err.      z    P>|z|     [95% Conf. Interval]
-------------------+----------------------------------------------------------------
preop_platelet_tot |    .998962   .0015957    -0.65   0.516     .9958394    1.002095
             _cons |    .171526    .068717    -4.40   0.000     .0782204    .3761314
------------------------------------------------------------------------------------
Note: _cons estimates baseline odds.

. logistic target_pneumonia preop_leuko_bin

Logistic regression                             Number of obs     =        611
                                                LR chi2(1)        =       1.51
                                                Prob > chi2       =     0.2194
Log likelihood =  -220.7935                     Pseudo R2         =     0.0034

----------------------------------------------------------------------------------
target_pneumonia | Odds Ratio   Std. Err.      z    P>|z|     [95% Conf. Interval]
-----------------+----------------------------------------------------------------
 preop_leuko_bin |   .5741455   .2772301    -1.15   0.250     .2228504    1.479212
           _cons |   .1404612   .0183256   -15.04   0.000     .1087683    .1813889
----------------------------------------------------------------------------------
Note: _cons estimates baseline odds.

. logistic target_pneumonia preop_leuko_tot

Logistic regression                             Number of obs     =        611
                                                LR chi2(1)        =       1.47
                                                Prob > chi2       =     0.2257
Log likelihood = -220.81366                     Pseudo R2         =     0.0033

----------------------------------------------------------------------------------
target_pneumonia | Odds Ratio   Std. Err.      z    P>|z|     [95% Conf. Interval]
-----------------+----------------------------------------------------------------
 preop_leuko_tot |   1.059728   .0494569     1.24   0.214     .9670952    1.161233
           _cons |   .0916921   .0306199    -7.15   0.000     .0476519    .1764347
----------------------------------------------------------------------------------
Note: _cons estimates baseline odds.

. logistic target_pneumonia preop_diabetes

Logistic regression                             Number of obs     =        611
                                                LR chi2(1)        =       0.72
                                                Prob > chi2       =     0.3946
Log likelihood = -221.18525                     Pseudo R2         =     0.0016

----------------------------------------------------------------------------------
target_pneumonia | Odds Ratio   Std. Err.      z    P>|z|     [95% Conf. Interval]
-----------------+----------------------------------------------------------------
  preop_diabetes |   1.362477   .4822407     0.87   0.382     .6808502    2.726509
           _cons |   .1281513   .0174278   -15.11   0.000     .0981668    .1672942
----------------------------------------------------------------------------------
Note: _cons estimates baseline odds.

. logistic target_pneumonia preop_weight

Logistic regression                             Number of obs     =        611
                                                LR chi2(1)        =       2.99
                                                Prob > chi2       =     0.0836
Log likelihood = -220.05063                     Pseudo R2         =     0.0068

----------------------------------------------------------------------------------
target_pneumonia | Odds Ratio   Std. Err.      z    P>|z|     [95% Conf. Interval]
-----------------+----------------------------------------------------------------
    preop_weight |   1.011881   .0068336     1.75   0.080     .9985753    1.025363
           _cons |    .048704   .0292329    -5.03   0.000     .0150197    .1579315
----------------------------------------------------------------------------------
Note: _cons estimates baseline odds.

. logistic target_pneumonia preop_height

Logistic regression                             Number of obs     =        611
                                                LR chi2(1)        =       1.46
                                                Prob > chi2       =     0.2265
Log likelihood = -220.81618                     Pseudo R2         =     0.0033

----------------------------------------------------------------------------------
target_pneumonia | Odds Ratio   Std. Err.      z    P>|z|     [95% Conf. Interval]
-----------------+----------------------------------------------------------------
    preop_height |   .1713387   .2498772    -1.21   0.226     .0098283    2.986977
```

```
           _cons |   3.002383   7.712809     0.43   0.669     .0195352    461.4387
------------------------------------------------------------------------------
Note: _cons estimates baseline odds.

. logistic target_pneumonia preop_bmi

Logistic regression                             Number of obs     =        611
                                                LR chi2(1)        =       6.52
                                                Prob > chi2       =     0.0107
Log likelihood = -218.28724                     Pseudo R2         =     0.0147

------------------------------------------------------------------------------
target_pneumonia | Odds Ratio   Std. Err.      z    P>|z|     [95% Conf. Interval]
-----------------+------------------------------------------------------------
       preop_bmi |   1.064806   .0257114     2.60   0.009     1.015587    1.116411
           _cons |   .0240884   .0164869    -5.44   0.000     .0062982    .0921296
------------------------------------------------------------------------------
Note: _cons estimates baseline odds.

. logistic target_pneumonia preop_ecog

Logistic regression                             Number of obs     =        611
                                                LR chi2(1)        =       1.25
                                                Prob > chi2       =     0.2643
Log likelihood = -220.92464                     Pseudo R2         =     0.0028

------------------------------------------------------------------------------
target_pneumonia | Odds Ratio   Std. Err.      z    P>|z|     [95% Conf. Interval]
-----------------+------------------------------------------------------------
      preop_ecog |   1.212052   .2048519     1.14   0.255     .8702774    1.688049
           _cons |   .1196294   .0194543   -13.06   0.000      .086979    .1645362
------------------------------------------------------------------------------
Note: _cons estimates baseline odds.

. logistic target_pneumonia preop_tstage

Logistic regression                             Number of obs     =        611
                                                LR chi2(1)        =       2.87
                                                Prob > chi2       =     0.0904
Log likelihood = -220.11381                     Pseudo R2         =     0.0065

------------------------------------------------------------------------------
target_pneumonia | Odds Ratio   Std. Err.      z    P>|z|     [95% Conf. Interval]
-----------------+------------------------------------------------------------
    preop_tstage |   1.550225   .4354009     1.56   0.119     .8939719    2.688226
           _cons |   .0384915   .0315138    -3.98   0.000     .0077351    .1915411
------------------------------------------------------------------------------
Note: _cons estimates baseline odds.

. logistic target_pneumonia preop_neoadj

Logistic regression                             Number of obs     =        611
                                                LR chi2(1)        =       0.67
                                                Prob > chi2       =     0.4118
Log likelihood = -221.21078                     Pseudo R2         =     0.0015

------------------------------------------------------------------------------
target_pneumonia | Odds Ratio   Std. Err.      z    P>|z|     [95% Conf. Interval]
-----------------+------------------------------------------------------------
    preop_neoadj |   .8762583   .1398851    -0.83   0.408     .6408364    1.198166
           _cons |   .1616324   .0417216    -7.06   0.000     .0974566    .2680686
------------------------------------------------------------------------------
Note: _cons estimates baseline odds.

. logistic target_pneumonia preop_asa

Logistic regression                             Number of obs     =        611
                                                LR chi2(1)        =       1.47
                                                Prob > chi2       =     0.2254
Log likelihood = -220.81265                     Pseudo R2         =     0.0033

------------------------------------------------------------------------------
target_pneumonia | Odds Ratio   Std. Err.      z    P>|z|     [95% Conf. Interval]
-----------------+------------------------------------------------------------
       preop_asa |   .8069558   .1429649    -1.21   0.226     .5702247    1.141967
           _cons |   .1745973   .0433115    -7.04   0.000     .1073703    .2839167
------------------------------------------------------------------------------
Note: _cons estimates baseline odds.

. logistic target_pneumonia preop_weightloss_bin

Logistic regression                             Number of obs     =        611
                                                LR chi2(1)        =       1.29
                                                Prob > chi2       =     0.2554
Log likelihood = -220.90085                     Pseudo R2         =     0.0029

--------------------------------------------------------------------------------------
    target_pneumonia | Odds Ratio   Std. Err.      z    P>|z|     [95% Conf. Interval]
---------------------+----------------------------------------------------------------
preop_weightloss_bin |   .7003022   .2259093    -1.10   0.269     .3721327    1.317872
               _cons |   .1439024   .0200372   -13.92   0.000     .1095332    .1890561
--------------------------------------------------------------------------------------
Note: _cons estimates baseline odds.

. logistic target_pneumonia preop_fev1_bin

Logistic regression                             Number of obs     =        611
                                                LR chi2(1)        =      11.64
                                                Prob > chi2       =     0.0006
Log likelihood = -215.72677                     Pseudo R2         =     0.0263
```

```
------------------------------------------------------------------------------
target_pneumonia | Odds Ratio   Std. Err.      z    P>|z|     [95% Conf. Interval]
-----------------+----------------------------------------------------------------
  preop_fev1_bin |   2.539562   .6720058     3.52   0.000     1.511883    4.265789
           _cons |   .1020882   .0161569   -14.42   0.000     .0748619    .1392162
------------------------------------------------------------------------------
Note: _cons estimates baseline odds.

. logistic target_pneumonia preop_fev1_tot

Logistic regression                             Number of obs     =        611
                                                LR chi2(1)        =       9.35
                                                Prob > chi2       =     0.0022
Log likelihood = -216.87408                     Pseudo R2         =     0.0211

------------------------------------------------------------------------------
target_pneumonia | Odds Ratio   Std. Err.      z    P>|z|     [95% Conf. Interval]
-----------------+----------------------------------------------------------------
  preop_fev1_tot |   .6009886   .1023609    -2.99   0.003     .4304165    .8391578
           _cons |   .6009521   .3007956    -1.02   0.309     .2253131    1.602852
------------------------------------------------------------------------------
Note: _cons estimates baseline odds.

. logistic target_pneumonia preop_vc_bin

Logistic regression                             Number of obs     =        611
                                                LR chi2(1)        =       6.59
                                                Prob > chi2       =     0.0102
Log likelihood = -218.25076                     Pseudo R2         =     0.0149

------------------------------------------------------------------------------
target_pneumonia | Odds Ratio   Std. Err.      z    P>|z|     [95% Conf. Interval]
-----------------+----------------------------------------------------------------
    preop_vc_bin |   2.110108   .5910162     2.67   0.008      1.21869    3.653559
           _cons |   .1121076   .0167195   -14.67   0.000     .0836929    .1501695
------------------------------------------------------------------------------
Note: _cons estimates baseline odds.

. logistic target_pneumonia preop_vc_tot

Logistic regression                             Number of obs     =        611
                                                LR chi2(1)        =       6.95
                                                Prob > chi2       =     0.0084
Log likelihood = -218.07505                     Pseudo R2         =     0.0157

------------------------------------------------------------------------------
target_pneumonia | Odds Ratio   Std. Err.      z    P>|z|     [95% Conf. Interval]
-----------------+----------------------------------------------------------------
    preop_vc_tot |   .6976682   .0964002    -2.61   0.009     .5321503    .9146683
           _cons |   .5252087   .2757488    -1.23   0.220     .1876843    1.469724
------------------------------------------------------------------------------
Note: _cons estimates baseline odds.

. logistic target_pneumonia psm_robot

Logistic regression                             Number of obs     =        611
                                                LR chi2(1)        =       1.39
                                                Prob > chi2       =     0.2386
Log likelihood = -220.85331                     Pseudo R2         =     0.0031

------------------------------------------------------------------------------
target_pneumonia | Odds Ratio   Std. Err.      z    P>|z|     [95% Conf. Interval]
-----------------+----------------------------------------------------------------
       psm_robot |   .6090909   .2711899    -1.11   0.265     .2545046    1.457702
           _cons |   .1407249   .0185008   -14.92   0.000     .1087591     .182086
------------------------------------------------------------------------------
Note: _cons estimates baseline odds.

.
. logistic target_pneumonia preop_diag preop_age preop_sex preop_smoke preop_alcohol preop_khk preop_aht preop_vhf preop_mi
preop_revasc preop_pavk preop_copd preop_liver preop_cr
> ea_tot preop_gfr_tot preop_quick_tot preop_albumin_tot preop_biliribin_tot preop_platelet_bin preop_platelet_tot
preop_leuko_bin preop_leuko_tot preop_diabetes preop_weight preo
> p_height preop_bmi preop_ecog preop_tstage preop_neoadj preop_asa preop_weightloss_bin preop_fev1_bin preop_fev1_tot
preop_vc_bin preop_vc_tot psm_robot

Logistic regression                             Number of obs     =        611
                                                LR chi2(36)       =      56.63
                                                Prob > chi2       =     0.0156
Log likelihood = -193.23406                     Pseudo R2         =     0.1278

----------------------------------------------------------------------------------
    target_pneumonia | Odds Ratio   Std. Err.      z    P>|z|     [95% Conf. Interval]
---------------------+----------------------------------------------------------------
          preop_diag |   .6326231   .2773847    -1.04   0.296      .267868    1.494064
           preop_age |   1.030439   .0259448     1.19   0.234     .9808226    1.082566
           preop_sex |   .6766491   .3989017    -0.66   0.508     .2130879    2.148663
         preop_smoke |   .9652471   .2068341    -0.17   0.869     .6342233    1.469044
       preop_alcohol |   .8430321   .1401301    -1.03   0.304     .6086338    1.167702
           preop_khk |   2.545186   1.280836     1.86   0.063     .9492094      6.8246
           preop_aht |   1.000203   .3008694     0.00   0.999     .5546763    1.803585
           preop_vhf |   .8666103   .4616931    -0.27   0.788     .3050281    2.462112
            preop_mi |   1.155817   .6826678     0.25   0.806     .3631936    3.678238
        preop_revasc |   .3334004    .228813    -1.60   0.109     .0868532    1.279813
          preop_pavk |   1.517129   .9486582     0.67   0.505     .4454181    5.167463
          preop_copd |    1.29609   .4190533     0.80   0.422     .6877394    2.442568
         preop_liver |   .6629907   .4446334    -0.61   0.540     .1780946    2.468108
      preop_crea_tot |   .8040614   1.081115    -0.16   0.871     .0576489     11.2147
       preop_gfr_tot |    1.00374   .0177446     0.21   0.833     .9695566    1.039128
     preop_quick_tot |   1.004471   .0100242     0.45   0.655     .9850148    1.024312
   preop_albumin_tot |   1.032309   .0379173     0.87   0.387     .9606043    1.109366
```

```
 preop_biliribin_tot |   3.053028   1.912407     1.78   0.075     .8944188    10.42127
  preop_platelet_bin |   .3256787    .266718    -1.37   0.171     .0654168    1.621398
  preop_platelet_tot |   .9984637   .0018594    -0.83   0.409     .9948259    1.002115
     preop_leuko_bin |   .8873336   .4689343    -0.23   0.821     .3149543    2.499921
     preop_leuko_tot |   1.064366   .0660533     1.01   0.315     .9424676    1.202031
      preop_diabetes |   1.046191   .4283657     0.11   0.912     .4689044    2.334198
        preop_weight |   1.179865    .101476     1.92   0.054     .9968354    1.396501
        preop_height |   1.54e-08   1.37e-07    -2.03   0.043     4.31e-16    .5519352
           preop_bmi |   .6383791   .1714509    -1.67   0.095     .3771114    1.080657
          preop_ecog |   1.015169   .2001605     0.08   0.939     .6897743    1.494065
        preop_tstage |   2.182758   .7671301     2.22   0.026     1.096102    4.346706
        preop_neoadj |   .9146731   .1982635    -0.41   0.681     .5980816    1.398851
           preop_asa |   .6895084   .1475092    -1.74   0.082     .4533553    1.048674
preop_weightloss_bin |   .6172502    .219861    -1.35   0.176     .3070917    1.240664
      preop_fev1_bin |   2.408109   1.115677     1.90   0.058     .9712132    5.970873
      preop_fev1_tot |   .8938355   .3566181    -0.28   0.778     .4089303    1.953736
        preop_vc_bin |   1.224192    .532008     0.47   0.642     .5223186    2.869219
        preop_vc_tot |   .9881699   .3374035    -0.03   0.972     .5060518    1.929604
           psm_robot |   1.275943   .6665867     0.47   0.641     .4582902    3.552399
               _cons |   4.47e+09   7.21e+10     1.38   0.168     .0000829    2.41e+23
--------------------------------------------------------------------------------------
Note: _cons estimates baseline odds.

.
```

```
  ___  ____  ____  ____  ____ (R)
 /__    /   ____/   /   ____/
___/   /   /___/   /   /___/   15.0   Copyright 1985-2017 StataCorp LLC
  Statistics/Data Analysis            StataCorp
                                      4905 Lakeway Drive
     Special Edition                  College Station, Texas 77845 USA
                                      800-STATA-PC        http://www.stata.com
                                      979-696-4600        stata@stata.com
                                      979-696-4601 (fax)

. logistic target_ai preop_diag

Logistic regression                             Number of obs     =        611
                                                LR chi2(1)        =       2.01
                                                Prob > chi2       =     0.1565
Log likelihood = -212.36349                     Pseudo R2         =     0.0047

------------------------------------------------------------------------------
   target_ai | Odds Ratio   Std. Err.      z    P>|z|     [95% Conf. Interval]
-------------+----------------------------------------------------------------
  preop_diag |   1.537864   .4550026     1.45   0.146     .8611444    2.746377
       _cons |   .1136364   .0169591   -14.57   0.000     .0848171    .1522478
------------------------------------------------------------------------------
Note: _cons estimates baseline odds.

. logistic target_ai preop_age

Logistic regression                             Number of obs     =        611
                                                LR chi2(1)        =       1.68
                                                Prob > chi2       =     0.1946
Log likelihood = -212.52618                     Pseudo R2         =     0.0039

------------------------------------------------------------------------------
   target_ai | Odds Ratio   Std. Err.      z    P>|z|     [95% Conf. Interval]
-------------+----------------------------------------------------------------
   preop_age |   1.017557   .0137386     1.29   0.197     .9909825    1.044843
       _cons |   .0411576   .0362642    -3.62   0.000      .007319    .2314456
------------------------------------------------------------------------------
Note: _cons estimates baseline odds.

. logistic target_ai preop_sex

Logistic regression                             Number of obs     =        611
                                                LR chi2(1)        =       1.04
                                                Prob > chi2       =     0.3085
Log likelihood = -212.84875                     Pseudo R2         =     0.0024

------------------------------------------------------------------------------
   target_ai | Odds Ratio   Std. Err.      z    P>|z|     [95% Conf. Interval]
-------------+----------------------------------------------------------------
   preop_sex |   .6801498   .2678662    -0.98   0.328     .3143203    1.471759
       _cons |   .1321586    .018154   -14.73   0.000     .1009647    .1729901
------------------------------------------------------------------------------
Note: _cons estimates baseline odds.

. logistic target_ai preop_smoke

Logistic regression                             Number of obs     =        611
                                                LR chi2(1)        =       0.06
                                                Prob > chi2       =     0.8100
Log likelihood = -213.33844                     Pseudo R2         =     0.0001

------------------------------------------------------------------------------
   target_ai | Odds Ratio   Std. Err.      z    P>|z|     [95% Conf. Interval]
-------------+----------------------------------------------------------------
 preop_smoke |   1.044254   .1880339     0.24   0.810     .7337269    1.486203
       _cons |   .1199805   .0265081    -9.60   0.000     .0778124    .1850004
------------------------------------------------------------------------------
Note: _cons estimates baseline odds.

. logistic target_ai preop_alcohol
```

```
Logistic regression                             Number of obs     =        611
                                                LR chi2(1)        =       1.56
                                                Prob > chi2       =     0.2114
Log likelihood = -212.58662                     Pseudo R2         =     0.0037

-------------------------------------------------------------------------------
    target_ai | Odds Ratio   Std. Err.      z    P>|z|     [95% Conf. Interval]
--------------+----------------------------------------------------------------
preop_alcohol |   .8287765   .1294038    -1.20   0.229     .6102843    1.125492
        _cons |   .1393335   .0211287   -13.00   0.000      .103509    .1875567
-------------------------------------------------------------------------------
Note: _cons estimates baseline odds.

. logistic target_ai preop_khk

Logistic regression                             Number of obs     =        611
                                                LR chi2(1)        =       3.19
                                                Prob > chi2       =     0.0739
Log likelihood = -211.76995                     Pseudo R2         =     0.0075

------------------------------------------------------------------------------
   target_ai | Odds Ratio   Std. Err.      z    P>|z|     [95% Conf. Interval]
-------------+----------------------------------------------------------------
   preop_khk |   1.841901   .6024263     1.87   0.062     .9702096    3.496768
       _cons |   .1134454   .0162901   -15.16   0.000     .0856168    .1503193
------------------------------------------------------------------------------
Note: _cons estimates baseline odds.

. logistic target_ai preop_aht

Logistic regression                             Number of obs     =        611
                                                LR chi2(1)        =      14.90
                                                Prob > chi2       =     0.0001
Log likelihood = -205.91597                     Pseudo R2         =     0.0349

------------------------------------------------------------------------------
   target_ai | Odds Ratio   Std. Err.      z    P>|z|     [95% Conf. Interval]
-------------+----------------------------------------------------------------
   preop_aht |   2.942105   .8782838     3.61   0.000     1.638912    5.281542
       _cons |   .0620155   .0159774   -10.79   0.000     .0374283    .1027543
------------------------------------------------------------------------------
Note: _cons estimates baseline odds.

. logistic target_ai preop_vhf

Logistic regression                             Number of obs     =        611
                                                LR chi2(1)        =       0.58
                                                Prob > chi2       =     0.4450
Log likelihood = -213.07569                     Pseudo R2         =     0.0014

------------------------------------------------------------------------------
   target_ai | Odds Ratio   Std. Err.      z    P>|z|     [95% Conf. Interval]
-------------+----------------------------------------------------------------
   preop_vhf |   1.405038   .6053625     0.79   0.430     .6038747    3.269109
       _cons |   .1215139   .0164764   -15.54   0.000     .0931556    .1585051
------------------------------------------------------------------------------
Note: _cons estimates baseline odds.

. logistic target_ai preop_mi

Logistic regression                             Number of obs     =        611
                                                LR chi2(1)        =       0.50
                                                Prob > chi2       =     0.4790
Log likelihood = -213.11678                     Pseudo R2         =     0.0012

------------------------------------------------------------------------------
   target_ai | Odds Ratio   Std. Err.      z    P>|z|     [95% Conf. Interval]
-------------+----------------------------------------------------------------
    preop_mi |   1.368853    .588855     0.73   0.465     .5890952    3.180737
       _cons |   .1217565   .0165111   -15.53   0.000     .0933389     .158826
------------------------------------------------------------------------------
Note: _cons estimates baseline odds.

. logistic target_ai preop_revasc

Logistic regression                             Number of obs     =        611
                                                LR chi2(1)        =       2.45
                                                Prob > chi2       =     0.1174
Log likelihood = -212.14159                     Pseudo R2         =     0.0057

------------------------------------------------------------------------------
   target_ai | Odds Ratio   Std. Err.      z    P>|z|     [95% Conf. Interval]
-------------+----------------------------------------------------------------
preop_revasc |   1.996078   .8303376     1.66   0.097     .8832649    4.510911
       _cons |   .1178782     .01609   -15.66   0.000     .0902085     .154035
------------------------------------------------------------------------------
Note: _cons estimates baseline odds.

. logistic target_ai preop_pavk

Logistic regression                             Number of obs     =        611
                                                LR chi2(1)        =       2.50
                                                Prob > chi2       =     0.1140
Log likelihood = -212.11821                     Pseudo R2         =     0.0059

------------------------------------------------------------------------------
   target_ai | Odds Ratio   Std. Err.      z    P>|z|     [95% Conf. Interval]
-------------+----------------------------------------------------------------
  preop_pavk |   2.455649   1.291489     1.71   0.088      .875987    6.883906
       _cons |   .1197719    .015968   -15.92   0.000     .0922301    .1555381
------------------------------------------------------------------------------
```

```
Note: _cons estimates baseline odds.

. logistic target_ai preop_copd

Logistic regression                             Number of obs     =        611
                                                LR chi2(1)        =       2.66
                                                Prob > chi2       =     0.1028
Log likelihood = -212.03659                     Pseudo R2         =     0.0062

------------------------------------------------------------------------------
   target_ai | Odds Ratio   Std. Err.      z    P>|z|     [95% Conf. Interval]
-------------+----------------------------------------------------------------
  preop_copd |   .4550399   .2592654    -1.38   0.167     .1489585     1.39006
       _cons |   .1324684   .0174371   -15.36   0.000      .102345    .1714581
------------------------------------------------------------------------------
Note: _cons estimates baseline odds.

. logistic target_ai preop_liver

Logistic regression                             Number of obs     =        611
                                                LR chi2(1)        =       0.21
                                                Prob > chi2       =     0.6500
Log likelihood = -213.26442                     Pseudo R2         =     0.0005

------------------------------------------------------------------------------
   target_ai | Odds Ratio   Std. Err.      z    P>|z|     [95% Conf. Interval]
-------------+----------------------------------------------------------------
 preop_liver |   .7622829   .4717103    -0.44   0.661     .2266636    2.563601
       _cons |   .1269531   .0167163   -15.67   0.000     .0980761    .1643325
------------------------------------------------------------------------------
Note: _cons estimates baseline odds.

. logistic target_ai preop_crea_tot

Logistic regression                             Number of obs     =        611
                                                LR chi2(1)        =       1.92
                                                Prob > chi2       =     0.1662
Log likelihood = -212.40876                     Pseudo R2         =     0.0045

--------------------------------------------------------------------------------
     target_ai | Odds Ratio   Std. Err.      z    P>|z|     [95% Conf. Interval]
---------------+----------------------------------------------------------------
preop_crea_tot |   2.167454   1.176318     1.43   0.154      .748147    6.279325
         _cons |   .0628475   .0319337    -5.45   0.000     .0232157    .1701355
--------------------------------------------------------------------------------
Note: _cons estimates baseline odds.

. logistic target_ai preop_gfr_tot

Logistic regression                             Number of obs     =        611
                                                LR chi2(1)        =       1.95
                                                Prob > chi2       =     0.1621
Log likelihood = -212.38997                     Pseudo R2         =     0.0046

-------------------------------------------------------------------------------
    target_ai | Odds Ratio   Std. Err.      z    P>|z|     [95% Conf. Interval]
--------------+----------------------------------------------------------------
preop_gfr_tot |   .9901436   .0070322    -1.39   0.163     .9764562    1.004023
        _cons |   .2903003   .1764113    -2.04   0.042     .0882236    .9552351
-------------------------------------------------------------------------------
Note: _cons estimates baseline odds.

. logistic target_ai preop_quick_tot

Logistic regression                             Number of obs     =        611
                                                LR chi2(1)        =       1.42
                                                Prob > chi2       =     0.2326
Log likelihood = -212.65497                     Pseudo R2         =     0.0033

---------------------------------------------------------------------------------
      target_ai | Odds Ratio   Std. Err.      z    P>|z|     [95% Conf. Interval]
----------------+----------------------------------------------------------------
preop_quick_tot |   1.011024   .0095882     1.16   0.248      .992405    1.029992
          _cons |   .0383925   .0398849    -3.14   0.002     .0050114    .2941283
---------------------------------------------------------------------------------
Note: _cons estimates baseline odds.

. logistic target_ai preop_albumin_tot

Logistic regression                             Number of obs     =        611
                                                LR chi2(1)        =       0.31
                                                Prob > chi2       =     0.5805
Log likelihood = -213.21461                     Pseudo R2         =     0.0007

-----------------------------------------------------------------------------------
        target_ai | Odds Ratio   Std. Err.      z    P>|z|     [95% Conf. Interval]
------------------+----------------------------------------------------------------
preop_albumin_tot |   1.017346   .0319252     0.55   0.584     .9566591    1.081882
            _cons |   .0628771   .0796891    -2.18   0.029     .0052444    .7538537
-----------------------------------------------------------------------------------
Note: _cons estimates baseline odds.

. logistic target_ai preop_biliribin_tot

Logistic regression                             Number of obs     =        611
                                                LR chi2(1)        =       0.05
                                                Prob > chi2       =     0.8241
Log likelihood = -213.34265                     Pseudo R2         =     0.0001

-------------------------------------------------------------------------------------
          target_ai | Odds Ratio   Std. Err.      z    P>|z|     [95% Conf. Interval]
```

```
--------------------+----------------------------------------------------------------
preop_biliribin_tot |   1.132906   .6303181     0.22   0.823     .3807227    3.371159
              _cons |   .1184447   .0332778    -7.59   0.000     .0682913    .2054311
-------------------------------------------------------------------------------------
Note: _cons estimates baseline odds.

. logistic target_ai preop_platelet_bin

Logistic regression                             Number of obs     =        611
                                                LR chi2(1)        =       0.81
                                                Prob > chi2       =     0.3694
Log likelihood = -212.96449                     Pseudo R2         =     0.0019

------------------------------------------------------------------------------------
         target_ai | Odds Ratio   Std. Err.      z    P>|z|     [95% Conf. Interval]
-------------------+----------------------------------------------------------------
preop_platelet_bin |   1.545364   .7182246     0.94   0.349      .621478    3.842695
             _cons |   .1213307   .0163171   -15.68   0.000     .0932176    .1579225
------------------------------------------------------------------------------------
Note: _cons estimates baseline odds.

. logistic target_ai preop_platelet_tot

Logistic regression                             Number of obs     =        611
                                                LR chi2(1)        =       0.41
                                                Prob > chi2       =     0.5207
Log likelihood = -213.16115                     Pseudo R2         =     0.0010

------------------------------------------------------------------------------------
         target_ai | Odds Ratio   Std. Err.      z    P>|z|     [95% Conf. Interval]
-------------------+----------------------------------------------------------------
preop_platelet_tot |   1.000963   .0014758     0.65   0.514     .9980749     1.00386
             _cons |   .0988148   .0384038    -5.96   0.000     .0461325    .2116589
------------------------------------------------------------------------------------
Note: _cons estimates baseline odds.

. logistic target_ai preop_leuko_bin

Logistic regression                             Number of obs     =        611
                                                LR chi2(1)        =       0.04
                                                Prob > chi2       =     0.8497
Log likelihood =  -213.3494                     Pseudo R2         =     0.0001

---------------------------------------------------------------------------------
      target_ai | Odds Ratio   Std. Err.      z    P>|z|     [95% Conf. Interval]
----------------+----------------------------------------------------------------
preop_leuko_bin |   .9237718    .389726    -0.19   0.851     .4040685    2.111905
          _cons |    .126294    .017161   -15.23   0.000     .0967654    .1648335
---------------------------------------------------------------------------------
Note: _cons estimates baseline odds.

. logistic target_ai preop_leuko_tot

Logistic regression                             Number of obs     =        611
                                                LR chi2(1)        =       7.59
                                                Prob > chi2       =     0.0059
Log likelihood =  -209.5732                     Pseudo R2         =     0.0178

---------------------------------------------------------------------------------
      target_ai | Odds Ratio   Std. Err.      z    P>|z|     [95% Conf. Interval]
----------------+----------------------------------------------------------------
preop_leuko_tot |   1.136185   .0506514     2.86   0.004     1.041123    1.239926
          _cons |   .0534632   .0180411    -8.68   0.000     .0275942     .103584
---------------------------------------------------------------------------------
Note: _cons estimates baseline odds.

. logistic target_ai preop_diabetes

Logistic regression                             Number of obs     =        611
                                                LR chi2(1)        =       0.01
                                                Prob > chi2       =     0.9257
Log likelihood = -213.36301                     Pseudo R2         =     0.0000

--------------------------------------------------------------------------------
     target_ai | Odds Ratio   Std. Err.      z    P>|z|     [95% Conf. Interval]
---------------+----------------------------------------------------------------
preop_diabetes |   .9636364   .3841449    -0.09   0.926     .4411532    2.104927
         _cons |   .1257862     .01723   -15.14   0.000     .0961693     .164524
--------------------------------------------------------------------------------
Note: _cons estimates baseline odds.

. logistic target_ai preop_weight

Logistic regression                             Number of obs     =        611
                                                LR chi2(1)        =       1.97
                                                Prob > chi2       =     0.1605
Log likelihood = -212.38255                     Pseudo R2         =     0.0046

------------------------------------------------------------------------------
   target_ai | Odds Ratio   Std. Err.      z    P>|z|     [95% Conf. Interval]
-------------+----------------------------------------------------------------
preop_weight |     1.0099   .0070184     1.42   0.156     .9962377     1.02375
       _cons |   .0540962   .0332429    -4.75   0.000     .0162215    .1804023
------------------------------------------------------------------------------
Note: _cons estimates baseline odds.

. logistic target_ai preop_height

Logistic regression                             Number of obs     =        611
                                                LR chi2(1)        =       3.57
                                                Prob > chi2       =     0.0589
```

```
Log likelihood = -211.58301                     Pseudo R2         =     0.0084

------------------------------------------------------------------------------
   target_ai | Odds Ratio   Std. Err.      z    P>|z|     [95% Conf. Interval]
-------------+----------------------------------------------------------------
preop_height |   17.42878   26.54851     1.88   0.061     .8803766    345.0369
       _cons |   .0007789   .0021207    -2.63   0.009     3.75e-06    .1618102
------------------------------------------------------------------------------
Note: _cons estimates baseline odds.

. logistic target_ai preop_bmi

Logistic regression                             Number of obs     =        611
                                                LR chi2(1)        =       0.40
                                                Prob > chi2       =     0.5287
Log likelihood = -213.16888                     Pseudo R2         =     0.0009

------------------------------------------------------------------------------
   target_ai | Odds Ratio   Std. Err.      z    P>|z|     [95% Conf. Interval]
-------------+----------------------------------------------------------------
   preop_bmi |    1.01659   .0263484     0.63   0.526     .9662383    1.069567
       _cons |    .080502   .0572953    -3.54   0.000     .0199519    .3248091
------------------------------------------------------------------------------
Note: _cons estimates baseline odds.

. logistic target_ai preop_ecog

Logistic regression                             Number of obs     =        611
                                                LR chi2(1)        =       0.08
                                                Prob > chi2       =     0.7804
Log likelihood =  -213.3285                     Pseudo R2         =     0.0002

------------------------------------------------------------------------------
   target_ai | Odds Ratio   Std. Err.      z    P>|z|     [95% Conf. Interval]
-------------+----------------------------------------------------------------
  preop_ecog |    .949336   .1781635    -0.28   0.782     .6571637    1.371407
       _cons |   .1287093   .0207446   -12.72   0.000     .0938466     .176523
------------------------------------------------------------------------------
Note: _cons estimates baseline odds.

. logistic target_ai preop_tstage

Logistic regression                             Number of obs     =        611
                                                LR chi2(1)        =       0.16
                                                Prob > chi2       =     0.6924
Log likelihood = -213.28912                     Pseudo R2         =     0.0004

------------------------------------------------------------------------------
   target_ai | Odds Ratio   Std. Err.      z    P>|z|     [95% Conf. Interval]
-------------+----------------------------------------------------------------
preop_tstage |   .9158231   .2007689    -0.40   0.688     .5959492    1.407388
       _cons |   .1599019   .0991961    -2.96   0.003      .047403    .5393882
------------------------------------------------------------------------------
Note: _cons estimates baseline odds.

. logistic target_ai preop_neoadj

Logistic regression                             Number of obs     =        611
                                                LR chi2(1)        =       2.29
                                                Prob > chi2       =     0.1301
Log likelihood = -212.22189                     Pseudo R2         =     0.0054

------------------------------------------------------------------------------
   target_ai | Odds Ratio   Std. Err.      z    P>|z|     [95% Conf. Interval]
-------------+----------------------------------------------------------------
preop_neoadj |   .7816487   .1255859    -1.53   0.125     .5704936    1.070958
       _cons |   .1774869   .0449599    -6.82   0.000     .1080302    .2916001
------------------------------------------------------------------------------
Note: _cons estimates baseline odds.

. logistic target_ai preop_asa

Logistic regression                             Number of obs     =        611
                                                LR chi2(1)        =       0.61
                                                Prob > chi2       =     0.4352
Log likelihood = -213.06291                     Pseudo R2         =     0.0014

------------------------------------------------------------------------------
   target_ai | Odds Ratio   Std. Err.      z    P>|z|     [95% Conf. Interval]
-------------+----------------------------------------------------------------
   preop_asa |   1.152269   .2096101     0.78   0.436     .8066982    1.645874
       _cons |   .1039008   .0287242    -8.19   0.000     .0604363    .1786241
------------------------------------------------------------------------------
Note: _cons estimates baseline odds.

. logistic target_ai preop_weightloss_bin

Logistic regression                             Number of obs     =        611
                                                LR chi2(1)        =       8.40
                                                Prob > chi2       =     0.0037
Log likelihood = -209.16636                     Pseudo R2         =     0.0197

--------------------------------------------------------------------------------------
           target_ai | Odds Ratio   Std. Err.      z    P>|z|     [95% Conf. Interval]
---------------------+----------------------------------------------------------------
preop_weightloss_bin |   .3468124   .1426192    -2.58   0.010     .1549017    .7764849
               _cons |   .1495098    .020524   -13.84   0.000     .1142407    .1956673
--------------------------------------------------------------------------------------
Note: _cons estimates baseline odds.

. logistic target_ai preop_fev1_bin
```

```
Logistic regression                             Number of obs     =        611
                                                LR chi2(1)        =       0.13
                                                Prob > chi2       =     0.7233
Log likelihood = -213.30466                     Pseudo R2         =     0.0003

--------------------------------------------------------------------------------
     target_ai | Odds Ratio   Std. Err.      z    P>|z|     [95% Conf. Interval]
---------------+----------------------------------------------------------------
preop_fev1_bin |    .894657   .2836487    -0.35   0.726     .4806016    1.665436
         _cons |    .128266   .0185405   -14.21   0.000     .0966213    .1702747
--------------------------------------------------------------------------------
Note: _cons estimates baseline odds.

. logistic target_ai preop_fev1_tot

Logistic regression                             Number of obs     =        611
                                                LR chi2(1)        =       0.11
                                                Prob > chi2       =     0.7387
Log likelihood = -213.31171                     Pseudo R2         =     0.0003

--------------------------------------------------------------------------------
     target_ai | Odds Ratio   Std. Err.      z    P>|z|     [95% Conf. Interval]
---------------+----------------------------------------------------------------
preop_fev1_tot |   1.056522   .1739559     0.33   0.738     .7651183     1.45891
         _cons |   .1057151   .0555463    -4.28   0.000     .0377474    .2960652
--------------------------------------------------------------------------------
Note: _cons estimates baseline odds.

. logistic target_ai preop_vc_bin

Logistic regression                             Number of obs     =        611
                                                LR chi2(1)        =       0.07
                                                Prob > chi2       =     0.7912
Log likelihood = -213.33231                     Pseudo R2         =     0.0002

------------------------------------------------------------------------------
   target_ai | Odds Ratio   Std. Err.      z    P>|z|     [95% Conf. Interval]
-------------+----------------------------------------------------------------
preop_vc_bin |   .9153947   .3079483    -0.26   0.793     .4734295    1.769952
       _cons |   .1272727   .0180574   -14.53   0.000     .0963756    .1680752
------------------------------------------------------------------------------
Note: _cons estimates baseline odds.

. logistic target_ai preop_vc_tot

Logistic regression                             Number of obs     =        611
                                                LR chi2(1)        =       0.71
                                                Prob > chi2       =     0.3990
Log likelihood = -213.01162                     Pseudo R2         =     0.0017

------------------------------------------------------------------------------
   target_ai | Odds Ratio   Std. Err.      z    P>|z|     [95% Conf. Interval]
-------------+----------------------------------------------------------------
preop_vc_tot |   1.123223   .1546842     0.84   0.399     .8575176    1.471258
       _cons |   .0790442   .0447057    -4.49   0.000     .0260885     .239492
------------------------------------------------------------------------------
Note: _cons estimates baseline odds.

. logistic target_ai psm_robot

Logistic regression                             Number of obs     =        611
                                                LR chi2(1)        =       1.00
                                                Prob > chi2       =     0.3180
Log likelihood = -212.86877                     Pseudo R2         =     0.0023

------------------------------------------------------------------------------
   target_ai | Odds Ratio   Std. Err.      z    P>|z|     [95% Conf. Interval]
-------------+----------------------------------------------------------------
   psm_robot |   .6539171   .2918519    -0.95   0.341     .2726592    1.568286
       _cons |   .1310782   .0177044   -15.04   0.000     .1005914    .1708048
------------------------------------------------------------------------------
Note: _cons estimates baseline odds.

.
. logistic target_ai preop_diag preop_age preop_sex preop_smoke preop_alcohol preop_khk preop_aht preop_vhf preop_mi
preop_revasc preop_pavk preop_copd preop_liver preop_crea_tot
>  preop_gfr_tot preop_quick_tot preop_albumin_tot preop_biliribin_tot preop_platelet_bin preop_platelet_tot preop_leuko_bin
preop_leuko_tot preop_diabetes preop_weight preop_heig
> ht preop_bmi preop_ecog preop_tstage preop_neoadj preop_asa preop_weightloss_bin preop_fev1_bin preop_fev1_tot
preop_vc_bin preop_vc_tot psm_robot

Logistic regression                             Number of obs     =        611
                                                LR chi2(36)       =      60.01
                                                Prob > chi2       =     0.0073
Log likelihood =  -183.3616                     Pseudo R2         =     0.1406

--------------------------------------------------------------------------------------
           target_ai | Odds Ratio   Std. Err.      z    P>|z|     [95% Conf. Interval]
---------------------+----------------------------------------------------------------
          preop_diag |   2.310569   .8731838     2.22   0.027     1.101647    4.846132
           preop_age |   1.024795    .026794     0.94   0.349     .9736024    1.078679
           preop_sex |   1.415994    .890799     0.55   0.580     .4126411    4.859041
         preop_smoke |   1.079326   .2385854     0.35   0.730      .699833    1.664604
       preop_alcohol |   .7860342   .1402737    -1.35   0.177     .5540362    1.115179
           preop_khk |   1.307555   .7721066     0.45   0.650     .4109875    4.159982
           preop_aht |   2.584473   .8759357     2.80   0.005     1.330089    5.021844
           preop_vhf |   1.018108   .5336737     0.03   0.973     .3644261    2.844318
            preop_mi |   .6427659   .4499564    -0.63   0.528      .162999    2.534666
        preop_revasc |   1.611995   1.171279     0.66   0.511     .3880422    6.696511
          preop_pavk |   2.103952   1.340246     1.17   0.243     .6036826    7.332684
```

```
           preop_copd |   .3014692   .2007494    -1.80   0.072     .0817382    1.111888
          preop_liver |    .943999   .6293319    -0.09   0.931     .2555669    3.486892
       preop_crea_tot |   .7077344    1.03222    -0.24   0.813     .0405887    12.34057
        preop_gfr_tot |   1.000223   .0191804     0.01   0.991     .9633283    1.038532
      preop_quick_tot |   1.023174   .0108426     2.16   0.031     1.002142    1.044648
    preop_albumin_tot |   1.027106   .0384173     0.72   0.475     .9545027    1.105231
  preop_biliribin_tot |    .721798   .4782202    -0.49   0.623     .1969974    2.644667
   preop_platelet_bin |   2.177683   1.212951     1.40   0.162     .7309414    6.487941
   preop_platelet_tot |   1.000311   .0017891     0.17   0.862     .9968101    1.003823
      preop_leuko_bin |   .9801346    .476376    -0.04   0.967     .3780726    2.540951
      preop_leuko_tot |    1.20783   .0713723     3.20   0.001      1.07574     1.35614
       preop_diabetes |   .7686087   .3472366    -0.58   0.560     .3170702    1.863181
         preop_weight |   1.030079   .0943466     0.32   0.746     .8608105    1.232632
         preop_height |   3.048991   28.41221     0.12   0.905     3.57e-08    2.61e+08
            preop_bmi |   .9216954    .267963    -0.28   0.779     .5213375    1.629505
           preop_ecog |    .852713   .1932214    -0.70   0.482     .5469202     1.32948
         preop_tstage |   1.272556   .3649224     0.84   0.401     .7254101    2.232391
          preop_neoadj |   .7445071   .1664722    -1.32   0.187       .48033    1.153979
            preop_asa |   1.207088   .2691586     0.84   0.399     .7797162    1.868707
preop_weightloss_bin |    .366332   .1640709    -2.24   0.025       .15228    .8812654
       preop_fev1_bin |   1.042478   .5303422     0.08   0.935     .3846215    2.825534
       preop_fev1_tot |   .9010651   .3929313    -0.24   0.811     .3833267    2.118084
         preop_vc_bin |   1.267274   .6333678     0.47   0.636     .4758286     3.37513
         preop_vc_tot |    1.39338   .4826533     0.96   0.338     .7066779    2.747372
            psm_robot |   .5440629   .2846882    -1.16   0.245     .1950951    1.517231
                _cons |   3.89e-06   .0000669    -0.72   0.469     8.90e-21    1.70e+09
------------------------------------------------------------------------------------
Note: _cons estimates baseline odds.

.
. logistic target_ai preop_diag preop_aht preop_quick_tot preop_leuko_tot preop_weightloss_bin

Logistic regression                             Number of obs     =        611
                                                LR chi2(5)        =      35.55
                                                Prob > chi2       =     0.0000
Log likelihood = -195.59436                     Pseudo R2         =     0.0833

------------------------------------------------------------------------------------
           target_ai | Odds Ratio   Std. Err.      z    P>|z|     [95% Conf. Interval]
---------------------+--------------------------------------------------------------
          preop_diag |   1.627234   .5040927     1.57   0.116     .8866666    2.986341
           preop_aht |   2.895973   .8825219     3.49   0.000      1.59367    5.262481
     preop_quick_tot |   1.017207   .0100239     1.73   0.083     .9977495    1.037045
     preop_leuko_tot |   1.163409   .0552633     3.19   0.001     1.059984    1.276925
preop_weightloss_bin |   .3405975   .1438994    -2.55   0.011     .1488047    .7795903
               _cons |   .0038732   .0047597    -4.52   0.000     .0003484    .0430618
------------------------------------------------------------------------------------
Note: _cons estimates baseline odds.

.




  ___  ____  ____  ____  ____ (R)
 /__    /   ____/   /   ____/
___/   /   /___/   /   /___/   15.0   Copyright 1985-2017 StataCorp LLC
  Statistics/Data Analysis            StataCorp
                                      4905 Lakeway Drive
     Special Edition                  College Station, Texas 77845 USA
                                      800-STATA-PC        http://www.stata.com
                                      979-696-4600        stata@stata.com
                                      979-696-4601 (fax)

. ologit target_clavien preop_age

Iteration 0:   log likelihood = -981.52826
Iteration 1:   log likelihood = -977.96471
Iteration 2:   log likelihood = -977.96282
Iteration 3:   log likelihood = -977.96282

Ordered logistic regression                     Number of obs     =        611
                                                LR chi2(1)        =       7.13
                                                Prob > chi2       =     0.0076
Log likelihood = -977.96282                     Pseudo R2         =     0.0036

------------------------------------------------------------------------------
target_clavien |      Coef.   Std. Err.      z    P>|z|     [95% Conf. Interval]
---------------+----------------------------------------------------------------
     preop_age |   .0197859   .0074462     2.66   0.008     .0051916    .0343803
---------------+----------------------------------------------------------------
         /cut1 |   .6396768   .4779252                     -.2970394    1.576393
         /cut2 |   .8581883   .4785229                     -.0796992    1.796076
         /cut3 |   1.212152   .4792392                      .2728604    2.151444
         /cut4 |   2.917729   .4898475                      1.957646    3.877813
         /cut5 |   3.427267   .4962155                      2.454702    4.399831
         /cut6 |   4.007845   .5080483                      3.012089    5.003602
         /cut7 |   5.022014   .5510373                         3.942    6.102027
------------------------------------------------------------------------------

. ologit target_clavien preop_diag

Iteration 0:   log likelihood = -981.52826
Iteration 1:   log likelihood = -981.01808
Iteration 2:   log likelihood = -981.01804

Ordered logistic regression                     Number of obs     =        611
                                                LR chi2(1)        =       1.02
                                                Prob > chi2       =     0.3124
Log likelihood = -981.01804                     Pseudo R2         =     0.0005
```

```
--------------------------------------------------------------------------------
target_clavien |      Coef.   Std. Err.      z    P>|z|     [95% Conf. Interval]
---------------+----------------------------------------------------------------
    preop_diag |   .1838522   .1819773     1.01   0.312    -.1728167    .5405212
---------------+----------------------------------------------------------------
         /cut1 |  -.5736223   .0922999                     -.7545267   -.3927179
         /cut2 |  -.3570458   .0904314                      -.534288   -.1798036
         /cut3 |  -.0050464   .0890494                       -.17958    .1694873
         /cut4 |   1.693269   .1167491                      1.464445    1.922093
         /cut5 |    2.20148   .1386094                       1.92981    2.473149
         /cut6 |   2.780482     .17414                      2.439174     3.12179
         /cut7 |   3.791978   .2733005                      3.256319    4.327638
--------------------------------------------------------------------------------

. ologit target_clavien preop_age

Iteration 0:   log likelihood = -981.52826
Iteration 1:   log likelihood = -977.96471
Iteration 2:   log likelihood = -977.96282
Iteration 3:   log likelihood = -977.96282

Ordered logistic regression                     Number of obs     =        611
                                                LR chi2(1)        =       7.13
                                                Prob > chi2       =     0.0076
Log likelihood = -977.96282                     Pseudo R2         =     0.0036

--------------------------------------------------------------------------------
target_clavien |      Coef.   Std. Err.      z    P>|z|     [95% Conf. Interval]
---------------+----------------------------------------------------------------
     preop_age |   .0197859   .0074462     2.66   0.008     .0051916    .0343803
---------------+----------------------------------------------------------------
         /cut1 |   .6396768   .4779252                     -.2970394    1.576393
         /cut2 |   .8581883   .4785229                     -.0796992    1.796076
         /cut3 |   1.212152   .4792392                      .2728604    2.151444
         /cut4 |   2.917729   .4898475                      1.957646    3.877813
         /cut5 |   3.427267   .4962155                      2.454702    4.399831
         /cut6 |   4.007845   .5080483                      3.012089    5.003602
         /cut7 |   5.022014   .5510373                         3.942    6.102027
--------------------------------------------------------------------------------

. ologit target_clavien preop_sex

Iteration 0:   log likelihood = -981.52826
Iteration 1:   log likelihood = -981.46485
Iteration 2:   log likelihood = -981.46485

Ordered logistic regression                     Number of obs     =        611
                                                LR chi2(1)        =       0.13
                                                Prob > chi2       =     0.7217
Log likelihood = -981.46485                     Pseudo R2         =     0.0001

--------------------------------------------------------------------------------
target_clavien |      Coef.   Std. Err.      z    P>|z|     [95% Conf. Interval]
---------------+----------------------------------------------------------------
     preop_sex |   .0706135   .1982215     0.36   0.722    -.3178935    .4591205
---------------+----------------------------------------------------------------
         /cut1 |  -.5993481   .0905671                     -.7768563   -.4218399
         /cut2 |  -.3831094   .0885271                     -.5566193   -.2095995
         /cut3 |  -.0310706   .0871216                     -.2018258    .1396846
         /cut4 |   1.667177   .1152558                       1.44128    1.893074
         /cut5 |   2.175033   .1372413                      1.906045    2.444021
         /cut6 |   2.753561   .1729161                      2.414652    3.092471
         /cut7 |   3.764557   .2724088                      3.230645    4.298468
--------------------------------------------------------------------------------

. ologit target_clavien preop_smoke

Iteration 0:   log likelihood = -981.52826
Iteration 1:   log likelihood =  -981.5219
Iteration 2:   log likelihood =  -981.5219

Ordered logistic regression                     Number of obs     =        611
                                                LR chi2(1)        =       0.01
                                                Prob > chi2       =     0.9102
Log likelihood =  -981.5219                     Pseudo R2         =     0.0000

--------------------------------------------------------------------------------
target_clavien |      Coef.   Std. Err.      z    P>|z|     [95% Conf. Interval]
---------------+----------------------------------------------------------------
   preop_smoke |   .0117176   .1038398     0.11   0.910    -.1918046    .2152398
---------------+----------------------------------------------------------------
         /cut1 |     -.5993   .1323467                     -.8586947   -.3399053
         /cut2 |   -.383085   .1309721                     -.6397856   -.1263844
         /cut3 |  -.0311025   .1298393                     -.2855829    .2233779
         /cut4 |   1.666599   .1489375                      1.374686    1.958511
         /cut5 |   2.174433    .166535                      1.848031    2.500836
         /cut6 |   2.753061   .1972526                      2.366453    3.139669
         /cut7 |   3.764298   .2887969                      3.198267     4.33033
--------------------------------------------------------------------------------

. ologit target_clavien preop_alcohol

Iteration 0:   log likelihood = -981.52826
Iteration 1:   log likelihood = -980.81531
Iteration 2:   log likelihood = -980.81524

Ordered logistic regression                     Number of obs     =        611
                                                LR chi2(1)        =       1.43
                                                Prob > chi2       =     0.2324
Log likelihood = -980.81524                     Pseudo R2         =     0.0007
```

```
------------------------------------------------------------------------------
target_clavien |      Coef.   Std. Err.      z    P>|z|     [95% Conf. Interval]
---------------+--------------------------------------------------------------
 preop_alcohol |  -.0940144   .0788496    -1.19   0.233    -.2485568    .0605281
---------------+--------------------------------------------------------------
         /cut1 |  -.6732586   .0997441                     -.8687535   -.4777638
         /cut2 |  -.4569086   .0977678                       -.64853   -.2652873
         /cut3 |  -.1042545   .0960949                     -.2925972    .0840881
         /cut4 |   1.597406   .1201621                      1.361893     1.83292
         /cut5 |   2.106474   .1409901                      1.830138    2.382809
         /cut6 |   2.685627   .1757703                      2.341123     3.03013
         /cut7 |   3.697044   .2742272                      3.159569     4.23452
------------------------------------------------------------------------------

. ologit target_clavien preop_khk

Iteration 0:   log likelihood = -981.52826
Iteration 1:   log likelihood = -979.94973
Iteration 2:   log likelihood = -979.94928
Iteration 3:   log likelihood = -979.94928

Ordered logistic regression                     Number of obs     =        611
                                                LR chi2(1)        =       3.16
                                                Prob > chi2       =     0.0756
Log likelihood = -979.94928                     Pseudo R2         =     0.0016

------------------------------------------------------------------------------
target_clavien |      Coef.   Std. Err.      z    P>|z|     [95% Conf. Interval]
---------------+--------------------------------------------------------------
     preop_khk |    .375409   .2113306     1.78   0.076    -.0387913    .7896093
---------------+--------------------------------------------------------------
         /cut1 |   -.560531   .0892853                      -.735527    -.385535
         /cut2 |  -.3429824   .0874493                     -.5143798   -.1715849
         /cut3 |   .0104842   .0863167                     -.1586933    .1796618
         /cut4 |   1.711139   .1151229                      1.485502    1.936775
         /cut5 |   2.219068   .1371624                      1.950235    2.487902
         /cut6 |   2.798834   .1730473                      2.459668    3.138001
         /cut7 |   3.811399   .2726747                      3.276967    4.345832
------------------------------------------------------------------------------

. ologit target_clavien preop_aht

Iteration 0:   log likelihood = -981.52826
Iteration 1:   log likelihood = -980.50199
Iteration 2:   log likelihood = -980.50183
Iteration 3:   log likelihood = -980.50183

Ordered logistic regression                     Number of obs     =        611
                                                LR chi2(1)        =       2.05
                                                Prob > chi2       =     0.1519
Log likelihood = -980.50183                     Pseudo R2         =     0.0010

------------------------------------------------------------------------------
target_clavien |      Coef.   Std. Err.      z    P>|z|     [95% Conf. Interval]
---------------+--------------------------------------------------------------
     preop_aht |    .211314   .1475971     1.43   0.152    -.0779711     .500599
---------------+--------------------------------------------------------------
         /cut1 |  -.4962321    .116432                     -.7244346   -.2680296
         /cut2 |  -.2794915   .1151351                     -.5051521   -.0538309
         /cut3 |   .0727592   .1142449                     -.1511567     .296675
         /cut4 |   1.772242   .1379269                       1.50191    2.042573
         /cut5 |   2.281289   .1572586                      1.973068    2.589511
         /cut6 |   2.861701   .1898244                      2.489652     3.23375
         /cut7 |   3.874786    .283923                      3.318308    4.431265
------------------------------------------------------------------------------

. ologit target_clavien preop_vhf

Iteration 0:   log likelihood = -981.52826
Iteration 1:   log likelihood = -978.92727
Iteration 2:   log likelihood = -978.92461
Iteration 3:   log likelihood = -978.92461

Ordered logistic regression                     Number of obs     =        611
                                                LR chi2(1)        =       5.21
                                                Prob > chi2       =     0.0225
Log likelihood = -978.92461                     Pseudo R2         =     0.0027

------------------------------------------------------------------------------
target_clavien |      Coef.   Std. Err.      z    P>|z|     [95% Conf. Interval]
---------------+--------------------------------------------------------------
     preop_vhf |   .6054983   .2649856     2.29   0.022     .0861361     1.12486
---------------+--------------------------------------------------------------
         /cut1 |  -.5662132   .0869927                     -.7367159   -.3957106
         /cut2 |  -.3480773   .0850505                     -.5147732   -.1813815
         /cut3 |   .0075701   .0840003                     -.1570674    .1722077
         /cut4 |   1.715841   .1141332                      1.492144    1.939538
         /cut5 |   2.223814     .13634                      1.956592    2.491035
         /cut6 |   2.803466   .1723555                      2.465656    3.141277
         /cut7 |   3.816317   .2722321                      3.282752    4.349882
------------------------------------------------------------------------------

. ologit target_clavien preop_mi

Iteration 0:   log likelihood = -981.52826
Iteration 1:   log likelihood = -981.19174
Iteration 2:   log likelihood = -981.19171

Ordered logistic regression                     Number of obs     =        611
                                                LR chi2(1)        =       0.67
```

```
                                                  Prob > chi2       =     0.4120
Log likelihood = -981.19171                       Pseudo R2         =     0.0003

------------------------------------------------------------------------------
target_clavien |      Coef.   Std. Err.      z    P>|z|     [95% Conf. Interval]
---------------+----------------------------------------------------------------
      preop_mi |   .2241527   .2732275     0.82   0.412    -.3113634    .7596689
---------------+----------------------------------------------------------------
         /cut1 |  -.5933848   .0872965                     -.7644828   -.4222868
         /cut2 |  -.3770443   .0851901                     -.5440138   -.2100748
         /cut3 |  -.0251194   .0836755                     -.1891204    .1388816
         /cut4 |   1.673004   .1124702                      1.452566    1.893441
         /cut5 |   2.181275   .1349955                      1.916688    2.445861
         /cut6 |   2.760483    .171282                      2.424776    3.096189
         /cut7 |   3.772448   .2715287                      3.240261    4.304634
------------------------------------------------------------------------------

. ologit target_clavien preop_revasc

Iteration 0:   log likelihood = -981.52826
Iteration 1:   log likelihood = -981.50739
Iteration 2:   log likelihood = -981.50739

Ordered logistic regression                       Number of obs     =        611
                                                  LR chi2(1)        =       0.04
                                                  Prob > chi2       =     0.8381
Log likelihood = -981.50739                       Pseudo R2         =     0.0000

------------------------------------------------------------------------------
target_clavien |      Coef.   Std. Err.      z    P>|z|     [95% Conf. Interval]
---------------+----------------------------------------------------------------
  preop_revasc |   .0586372   .2869263     0.20   0.838    -.5037279    .6210023
---------------+----------------------------------------------------------------
         /cut1 |  -.6065968   .0871376                     -.7773833   -.4358102
         /cut2 |  -.3903728   .0849967                     -.5569633   -.2237822
         /cut3 |  -.0384434   .0833961                     -.2018967      .12501
         /cut4 |   1.659309   .1119935                      1.439806    1.878812
         /cut5 |   2.167192    .134528                      1.903522    2.430862
         /cut6 |    2.74586   .1708494                      2.411002    3.080719
         /cut7 |   3.757099   .2711888                      3.225579     4.28862
------------------------------------------------------------------------------

. ologit target_clavien preop_pavk

Iteration 0:   log likelihood = -981.52826
Iteration 1:   log likelihood = -981.10124
Iteration 2:   log likelihood = -981.10118

Ordered logistic regression                       Number of obs     =        611
                                                  LR chi2(1)        =       0.85
                                                  Prob > chi2       =     0.3554
Log likelihood = -981.10118                       Pseudo R2         =     0.0004

------------------------------------------------------------------------------
target_clavien |      Coef.   Std. Err.      z    P>|z|     [95% Conf. Interval]
---------------+----------------------------------------------------------------
    preop_pavk |   .3398446   .3675006     0.92   0.355    -.3804433    1.060133
---------------+----------------------------------------------------------------
         /cut1 |  -.5975933   .0859028                     -.7659596    -.429227
         /cut2 |  -.3808158    .083823                     -.5451059   -.2165256
         /cut3 |  -.0281981   .0824237                     -.1897457    .1333494
         /cut4 |   1.670379   .1115776                       1.45169    1.889067
         /cut5 |   2.178111   .1341378                      1.915206    2.441016
         /cut6 |   2.756676   .1704891                      2.422524    3.090829
         /cut7 |   3.767664   .2709175                      3.236675    4.298652
------------------------------------------------------------------------------

. ologit target_clavien preop_copd

Iteration 0:   log likelihood = -981.52826
Iteration 1:   log likelihood = -981.13935
Iteration 2:   log likelihood =  -981.1393

Ordered logistic regression                       Number of obs     =        611
                                                  LR chi2(1)        =       0.78
                                                  Prob > chi2       =     0.3778
Log likelihood =  -981.1393                       Pseudo R2         =     0.0004

------------------------------------------------------------------------------
target_clavien |      Coef.   Std. Err.      z    P>|z|     [95% Conf. Interval]
---------------+----------------------------------------------------------------
    preop_copd |   .1830847   .2074226     0.88   0.377    -.2234561    .5896255
---------------+----------------------------------------------------------------
         /cut1 |  -.5930072   .0870495                     -.7636211   -.4223932
         /cut2 |  -.3766252   .0849443                      -.543113   -.2101375
         /cut3 |  -.0244099   .0835086                     -.1880838     .139264
         /cut4 |   1.674716   .1125868                       1.45405    1.895382
         /cut5 |   2.182809   .1350564                      1.918103    2.447515
         /cut6 |   2.761873   .1713133                      2.426105     3.09764
         /cut7 |   3.774009   .2715727                      3.241736    4.306281
------------------------------------------------------------------------------

. ologit target_clavien preop_liver

Iteration 0:   log likelihood = -981.52826
Iteration 1:   log likelihood = -981.11952
Iteration 2:   log likelihood =  -981.1195

Ordered logistic regression                       Number of obs     =        611
                                                  LR chi2(1)        =       0.82
                                                  Prob > chi2       =     0.3659
```

```
Log likelihood =  -981.1195                     Pseudo R2         =     0.0004

------------------------------------------------------------------------------
target_clavien |      Coef.   Std. Err.      z    P>|z|     [95% Conf. Interval]
---------------+----------------------------------------------------------------
   preop_liver |  -.2923422   .3241039    -0.90   0.367    -.9275742    .3428898
---------------+----------------------------------------------------------------
         /cut1 |  -.6277803   .0868475                     -.7979983   -.4575623
         /cut2 |  -.4112367   .0845855                     -.5770212   -.2454522
         /cut3 |  -.0586579   .0829019                     -.2211426    .1038268
         /cut4 |   1.641168   .1112937                      1.423036      1.8593
         /cut5 |   2.149525   .1338379                      1.887208    2.411843
         /cut6 |   2.728318   .1702229                      2.394688    3.061949
         /cut7 |   3.739179   .2707711                      3.208478    4.269881
------------------------------------------------------------------------------

. ologit target_clavien preop_crea_tot

Iteration 0:   log likelihood = -981.52826
Iteration 1:   log likelihood = -978.36014
Iteration 2:   log likelihood = -978.35218
Iteration 3:   log likelihood = -978.35218

Ordered logistic regression                     Number of obs     =        611
                                                LR chi2(1)        =       6.35
                                                Prob > chi2       =     0.0117
Log likelihood = -978.35218                     Pseudo R2         =     0.0032

------------------------------------------------------------------------------
target_clavien |      Coef.   Std. Err.      z    P>|z|     [95% Conf. Interval]
---------------+----------------------------------------------------------------
preop_crea_tot |   .8881583   .3470551     2.56   0.010     .2079428    1.568374
---------------+----------------------------------------------------------------
         /cut1 |   .1608635   .3129202                     -.4524489    .7741759
         /cut2 |    .378531   .3130046                     -.2349468    .9920087
         /cut3 |    .733757   .3140685                       .118194     1.34932
         /cut4 |   2.443843   .3295912                      1.797856    3.089829
         /cut5 |   2.953305   .3390982                      2.288685    3.617925
         /cut6 |   3.535264   .3572197                      2.835126    4.235402
         /cut7 |   4.552139   .4175773                      3.733703    5.370576
------------------------------------------------------------------------------

. ologit target_clavien preop_gfr_tot

Iteration 0:   log likelihood = -981.52826
Iteration 1:   log likelihood = -977.11707
Iteration 2:   log likelihood = -977.11431
Iteration 3:   log likelihood = -977.11431

Ordered logistic regression                     Number of obs     =        611
                                                LR chi2(1)        =       8.83
                                                Prob > chi2       =     0.0030
Log likelihood = -977.11431                     Pseudo R2         =     0.0045

------------------------------------------------------------------------------
target_clavien |      Coef.   Std. Err.      z    P>|z|     [95% Conf. Interval]
---------------+----------------------------------------------------------------
 preop_gfr_tot |  -.0119344   .0040387    -2.95   0.003    -.0198501   -.0040186
---------------+----------------------------------------------------------------
         /cut1 |  -1.646449   .3610245                     -2.354044   -.9388543
         /cut2 |  -1.428261   .3597246                     -2.133308   -.7232135
         /cut3 |   -1.07268   .3579142                     -1.774179   -.3711808
         /cut4 |   .6403928   .3585844                     -.0624196    1.343205
         /cut5 |   1.149555   .3655972                      .4329978    1.866113
         /cut6 |   1.729775   .3797228                      .9855323    2.474018
         /cut7 |   2.745033   .4328452                      1.896672    3.593394
------------------------------------------------------------------------------

. ologit target_clavien preop_quick_tot

Iteration 0:   log likelihood = -981.52826
Iteration 1:   log likelihood = -981.49916
Iteration 2:   log likelihood = -981.49915

Ordered logistic regression                     Number of obs     =        611
                                                LR chi2(1)        =       0.06
                                                Prob > chi2       =     0.8093
Log likelihood = -981.49915                     Pseudo R2         =     0.0000

-------------------------------------------------------------------------------
 target_clavien |      Coef.   Std. Err.      z    P>|z|     [95% Conf. Interval]
----------------+----------------------------------------------------------------
preop_quick_tot |   -.001196   .0049578    -0.24   0.809    -.0109132    .0085211
----------------+----------------------------------------------------------------
          /cut1 |  -.7387263   .5371624                     -1.791545    .3140926
          /cut2 |  -.5225544   .5369617                      -1.57498    .5298712
          /cut3 |  -.1706999   .5373203                     -1.223828    .8824284
          /cut4 |   1.526775   .5439297                      .4606924    2.592858
          /cut5 |   2.034515    .549356                       .957797    3.111233
          /cut6 |   2.613087    .559324                      1.516832    3.709342
          /cut7 |    3.62459      .5963                      2.455863    4.793316
-------------------------------------------------------------------------------

. ologit target_clavien preop_albumin_tot

Iteration 0:   log likelihood = -981.52826
Iteration 1:   log likelihood = -979.63938
Iteration 2:   log likelihood = -979.63889
Iteration 3:   log likelihood = -979.63889

Ordered logistic regression                     Number of obs     =        611
```

```
                                                  LR chi2(1)        =       3.78
                                                  Prob > chi2       =     0.0519
Log likelihood = -979.63889                       Pseudo R2         =     0.0019

------------------------------------------------------------------------------------
   target_clavien |      Coef.   Std. Err.      z    P>|z|     [95% Conf. Interval]
------------------+-----------------------------------------------------------------
preop_albumin_tot |   .0342749   .0176978     1.94   0.053    -.0004122    .0689619
------------------+-----------------------------------------------------------------
            /cut1 |   .7550477   .7100647                     -.6366535    2.146749
            /cut2 |   .9721878   .7103842                     -.4201397    2.364515
            /cut3 |    1.32643   .7115513                      -.068185    2.721045
            /cut4 |   3.033393   .7214558                      1.619366    4.447421
            /cut5 |   3.542404   .7260377                      2.119397    4.965412
            /cut6 |   4.122305   .7344104                      2.682887    5.561723
            /cut7 |   5.133724   .7642436                      3.635834    6.631614
------------------------------------------------------------------------------------

. ologit target_clavien preop_biliribin_tot

Iteration 0:   log likelihood = -981.52826
Iteration 1:   log likelihood = -981.19298
Iteration 2:   log likelihood = -981.19293

Ordered logistic regression                       Number of obs     =        611
                                                  LR chi2(1)        =       0.67
                                                  Prob > chi2       =     0.4128
Log likelihood = -981.19293                       Pseudo R2         =     0.0003

--------------------------------------------------------------------------------------
     target_clavien |      Coef.   Std. Err.      z    P>|z|     [95% Conf. Interval]
--------------------+-----------------------------------------------------------------
preop_biliribin_tot |   .2688064   .3297141     0.82   0.415    -.3774212    .9150341
--------------------+-----------------------------------------------------------------
              /cut1 |  -.4921464   .1681456                     -.8217058    -.162587
              /cut2 |  -.2756614   .1673699                     -.6037004    .0523777
              /cut3 |   .0768136   .1671881                      -.250869    .4044961
              /cut4 |    1.77502   .1840105                      1.414366    2.135674
              /cut5 |   2.282869   .1985835                      1.893652    2.672085
              /cut6 |   2.861859     .22512                      2.420631    3.303086
              /cut7 |   3.873487   .3085447                      3.268751    4.478224
--------------------------------------------------------------------------------------

. ologit target_clavien preop_platelet_bin

Iteration 0:   log likelihood = -981.52826
Iteration 1:   log likelihood = -981.50273
Iteration 2:   log likelihood = -981.50273

Ordered logistic regression                       Number of obs     =        611
                                                  LR chi2(1)        =       0.05
                                                  Prob > chi2       =     0.8212
Log likelihood = -981.50273                       Pseudo R2         =     0.0000

-------------------------------------------------------------------------------------
    target_clavien |      Coef.   Std. Err.      z    P>|z|     [95% Conf. Interval]
-------------------+-----------------------------------------------------------------
preop_platelet_bin |  -.0671303   .2972627    -0.23   0.821    -.6497546     .5154939
-------------------+-----------------------------------------------------------------
             /cut1 |  -.6151501   .0869077                      -.785486   -.4448142
             /cut2 |  -.3989604   .0847608                     -.5650886   -.2328322
             /cut3 |   -.046966    .083249                      -.210131     .116199
             /cut4 |   1.650954   .1118861                      1.431661    1.870247
             /cut5 |   2.158776   .1344019                      1.895353    2.422199
             /cut6 |   2.737404   .1706614                      2.402914    3.071894
             /cut7 |   3.748719   .2709847                      3.217598    4.279839
-------------------------------------------------------------------------------------

. ologit target_clavien preop_platelet_tot

Iteration 0:   log likelihood = -981.52826
Iteration 1:   log likelihood = -980.39596
Iteration 2:   log likelihood = -980.39578
Iteration 3:   log likelihood = -980.39578

Ordered logistic regression                       Number of obs     =        611
                                                  LR chi2(1)        =       2.26
                                                  Prob > chi2       =     0.1323
Log likelihood = -980.39578                       Pseudo R2         =     0.0012

-------------------------------------------------------------------------------------
    target_clavien |      Coef.   Std. Err.      z    P>|z|     [95% Conf. Interval]
-------------------+-----------------------------------------------------------------
preop_platelet_tot |  -.0013101   .0008739    -1.50   0.134    -.0030229     .0004027
-------------------+-----------------------------------------------------------------
             /cut1 |  -.9313406   .2302163                     -1.382556    -.480125
             /cut2 |  -.7142602   .2287963                     -1.162693   -.2658277
             /cut3 |  -.3601338   .2267717                     -.8045982    .0843305
             /cut4 |   1.341942   .2354458                      .8804765    1.803407
             /cut5 |   1.849706    .246907                      1.365777    2.333635
             /cut6 |   2.428738   .2681346                      1.903204    2.954272
             /cut7 |   3.439769   .3409394                       2.77154    4.107998
-------------------------------------------------------------------------------------

. ologit target_clavien preop_leuko_bin

Iteration 0:   log likelihood = -981.52826
Iteration 1:   log likelihood = -979.24189
Iteration 2:   log likelihood = -979.24118
Iteration 3:   log likelihood = -979.24118
```

```
Ordered logistic regression                     Number of obs     =        611
                                                LR chi2(1)        =       4.57
                                                Prob > chi2       =     0.0325
Log likelihood = -979.24118                     Pseudo R2         =     0.0023

--------------------------------------------------------------------------------
 target_clavien |      Coef.   Std. Err.      z    P>|z|     [95% Conf. Interval]
----------------+---------------------------------------------------------------
preop_leuko_bin |  -.4955335   .2334357    -2.12   0.034     -.953059   -.0380079
----------------+---------------------------------------------------------------
          /cut1 |  -.6705025    .089461                      -.8458427   -.4951622
          /cut2 |   -.453601   .0872112                      -.6245318   -.2826703
          /cut3 |   -.100145   .0854434                      -.2676109    .0673209
          /cut4 |   1.605438   .1125215                         1.3849    1.825976
          /cut5 |    2.11482   .1347966                       1.850623    2.379017
          /cut6 |   2.695028   .1709039                       2.360062    3.029993
          /cut7 |   3.708016   .2711237                       3.176623    4.239408
--------------------------------------------------------------------------------

. ologit target_clavien preop_leuko_tot

Iteration 0:   log likelihood = -981.52826
Iteration 1:   log likelihood = -979.80862
Iteration 2:   log likelihood = -979.80758
Iteration 3:   log likelihood = -979.80758

Ordered logistic regression                     Number of obs     =        611
                                                LR chi2(1)        =       3.44
                                                Prob > chi2       =     0.0636
Log likelihood = -979.80758                     Pseudo R2         =     0.0018

--------------------------------------------------------------------------------
 target_clavien |      Coef.   Std. Err.      z    P>|z|     [95% Conf. Interval]
----------------+---------------------------------------------------------------
preop_leuko_tot |   .0553174   .0297323     1.86   0.063    -.0029568    .1135917
----------------+---------------------------------------------------------------
          /cut1 |  -.2648015   .2040458                      -.6647239    .1351209
          /cut2 |  -.0481779   .2034309                      -.4468952    .3505393
          /cut3 |   .3050188   .2035663                      -.0939638    .7040013
          /cut4 |   2.010163   .2218114                       1.575421    2.444906
          /cut5 |   2.519138   .2347245                       2.059087     2.97919
          /cut6 |   3.098472   .2576517                       2.593484     3.60346
          /cut7 |   4.111363   .3333381                       3.458032    4.764694
--------------------------------------------------------------------------------

. ologit target_clavien preop_diabetes

Iteration 0:   log likelihood = -981.52826
Iteration 1:   log likelihood = -980.59378
Iteration 2:   log likelihood = -980.59358
Iteration 3:   log likelihood = -980.59358

Ordered logistic regression                     Number of obs     =        611
                                                LR chi2(1)        =       1.87
                                                Prob > chi2       =     0.1715
Log likelihood = -980.59358                     Pseudo R2         =     0.0010

-------------------------------------------------------------------------------
target_clavien |      Coef.   Std. Err.      z    P>|z|     [95% Conf. Interval]
---------------+---------------------------------------------------------------
preop_diabetes |   .3152519   .2304941     1.37   0.171    -.1365082    .7670119
---------------+---------------------------------------------------------------
         /cut1 |   -.576039   .0884104                      -.7493203   -.4027578
         /cut2 |  -.3594743   .0863784                      -.5287727   -.1901758
         /cut3 |  -.0069747   .0850407                      -.1736513    .1597019
         /cut4 |   1.693652   .1140997                       1.470021    1.917283
         /cut5 |   2.203263   .1365942                       1.935544    2.470983
         /cut6 |   2.783242   .1726502                       2.444854     3.12163
         /cut7 |   3.795397   .2724013                         3.2615    4.329294
-------------------------------------------------------------------------------

. ologit target_clavien preop_weight

Iteration 0:   log likelihood = -981.52826
Iteration 1:   log likelihood = -980.90855
Iteration 2:   log likelihood = -980.90847

Ordered logistic regression                     Number of obs     =        611
                                                LR chi2(1)        =       1.24
                                                Prob > chi2       =     0.2655
Log likelihood = -980.90847                     Pseudo R2         =     0.0006

-------------------------------------------------------------------------------
target_clavien |      Coef.   Std. Err.      z    P>|z|     [95% Conf. Interval]
---------------+---------------------------------------------------------------
  preop_weight |   .0045568   .0040894     1.11   0.265    -.0034582    .0125718
---------------+---------------------------------------------------------------
         /cut1 |  -.2317447   .3503959                       -.918508    .4550186
         /cut2 |   -.015367   .3500705                      -.7014926    .6707586
         /cut3 |   .3376547   .3507065                      -.3497173    1.025027
         /cut4 |   2.039933   .3633241                       1.327831    2.752035
         /cut5 |   2.548564   .3716715                       1.820101    3.277027
         /cut6 |   3.127454   .3865915                       2.369749     3.88516
         /cut7 |   4.138779   .4403513                       3.275707    5.001852
-------------------------------------------------------------------------------

. ologit target_clavien preop_height

Iteration 0:   log likelihood = -981.52826
Iteration 1:   log likelihood = -981.38544
Iteration 2:   log likelihood = -981.38543
```

```
Ordered logistic regression                     Number of obs     =        611
                                                LR chi2(1)        =       0.29
                                                Prob > chi2       =     0.5930
Log likelihood = -981.38543                     Pseudo R2         =     0.0001

------------------------------------------------------------------------------
target_clavien |      Coef.   Std. Err.      z    P>|z|     [95% Conf. Interval]
---------------+--------------------------------------------------------------
  preop_height |  -.4509119   .8436931    -0.53   0.593     -2.10452    1.202696
---------------+--------------------------------------------------------------
         /cut1 |  -1.407543   1.493286                      -4.33433    1.519243
         /cut2 |  -1.191056   1.492615                     -4.116528    1.734416
         /cut3 |  -.8390615   1.492498                     -3.764305    2.086182
         /cut4 |   .8581386   1.495175                     -2.072351    3.788628
         /cut5 |   1.366132    1.49668                     -1.567306    4.299571
         /cut6 |   1.944974   1.499839                     -.9946559    4.884605
         /cut7 |   2.956091   1.514594                      -.012459    5.924641
------------------------------------------------------------------------------

. ologit target_clavien preop_bmi

Iteration 0:   log likelihood = -981.52826
Iteration 1:   log likelihood = -980.32243
Iteration 2:   log likelihood = -980.32215
Iteration 3:   log likelihood = -980.32215

Ordered logistic regression                     Number of obs     =        611
                                                LR chi2(1)        =       2.41
                                                Prob > chi2       =     0.1204
Log likelihood = -980.32215                     Pseudo R2         =     0.0012

------------------------------------------------------------------------------
target_clavien |      Coef.   Std. Err.      z    P>|z|     [95% Conf. Interval]
---------------+--------------------------------------------------------------
     preop_bmi |   .0231354    .014889     1.55   0.120    -.0060464    .0523172
---------------+--------------------------------------------------------------
         /cut1 |   .0030112   .4037516                     -.7883274    .7943498
         /cut2 |     .21995   .4038279                     -.5715381    1.011438
         /cut3 |    .573616   .4047101                     -.2196012    1.366833
         /cut4 |   2.277297   .4164862                      1.460999    3.093595
         /cut5 |   2.786414   .4239976                      1.955394    3.617434
         /cut6 |   3.365793    .437382                       2.50854    4.223046
         /cut7 |   4.377236   .4856056                      3.425467    5.329006
------------------------------------------------------------------------------

. ologit target_clavien preop_ecog

Iteration 0:   log likelihood = -981.52826
Iteration 1:   log likelihood = -981.42379
Iteration 2:   log likelihood = -981.42379

Ordered logistic regression                     Number of obs     =        611
                                                LR chi2(1)        =       0.21
                                                Prob > chi2       =     0.6476
Log likelihood = -981.42379                     Pseudo R2         =     0.0001

------------------------------------------------------------------------------
target_clavien |      Coef.   Std. Err.      z    P>|z|     [95% Conf. Interval]
---------------+--------------------------------------------------------------
    preop_ecog |   .0484904   .1060566     0.46   0.648    -.1593766    .2563575
---------------+--------------------------------------------------------------
         /cut1 |  -.5848881   .1018574                      -.784525   -.3852512
         /cut2 |  -.3686078   .1001168                     -.5648331   -.1723824
         /cut3 |   -.016489   .0990021                     -.2105295    .1775515
         /cut4 |   1.681498   .1244086                      1.437662    1.925335
         /cut5 |   2.189207    .144894                       1.90522    2.473194
         /cut6 |   2.767822   .1791109                      2.416771    3.118873
         /cut7 |   3.779259   .2765768                      3.237178    4.321339
------------------------------------------------------------------------------

. ologit target_clavien preop_tstage

Iteration 0:   log likelihood = -981.52826
Iteration 1:   log likelihood = -981.50748
Iteration 2:   log likelihood = -981.50748

Ordered logistic regression                     Number of obs     =        611
                                                LR chi2(1)        =       0.04
                                                Prob > chi2       =     0.8384
Log likelihood = -981.50748                     Pseudo R2         =     0.0000

------------------------------------------------------------------------------
target_clavien |      Coef.   Std. Err.      z    P>|z|     [95% Conf. Interval]
---------------+--------------------------------------------------------------
  preop_tstage |   -.026637   .1305891    -0.20   0.838    -.2825869     .229313
---------------+--------------------------------------------------------------
         /cut1 |  -.6852817   .3749902                     -1.420249    .0496854
         /cut2 |  -.4690693   .3744726                     -1.203022    .2648835
         /cut3 |  -.1169521   .3735792                     -.8491538    .6152496
         /cut4 |   1.581528    .377957                       .840746     2.32231
         /cut5 |   2.089374   .3851371                      1.334519    2.844229
         /cut6 |     2.6678   .3998918                      1.884026    3.451573
         /cut7 |   3.678775   .4526155                      2.791665    4.565885
------------------------------------------------------------------------------

. ologit target_clavien preop_neoadj

Iteration 0:   log likelihood = -981.52826
Iteration 1:   log likelihood = -981.06367
Iteration 2:   log likelihood = -981.06364
```

```
Ordered logistic regression                     Number of obs     =        611
                                                LR chi2(1)        =       0.93
                                                Prob > chi2       =     0.3351
Log likelihood = -981.06364                     Pseudo R2         =     0.0005

------------------------------------------------------------------------------
target_clavien |      Coef.   Std. Err.      z    P>|z|     [95% Conf. Interval]
---------------+--------------------------------------------------------------
  preop_neoadj |  -.0922078   .0956946    -0.96   0.335    -.2797657    .0953501
---------------+--------------------------------------------------------------
         /cut1 |  -.7473949   .1653332                     -1.071442   -.4233479
         /cut2 |  -.5308846   .1638933                     -.8521097   -.2096596
         /cut3 |  -.1783423   .1625442                      -.496923    .1402385
         /cut4 |   1.521051   .1770817                      1.173977    1.868125
         /cut5 |   2.028874   .1920458                      1.652471    2.405276
         /cut6 |   2.607534   .2188789                       2.17854    3.036529
         /cut7 |   3.619168   .3034457                      3.024425     4.21391
------------------------------------------------------------------------------

. ologit target_clavien preop_asa

Iteration 0:   log likelihood = -981.52826
Iteration 1:   log likelihood = -981.51666
Iteration 2:   log likelihood = -981.51666

Ordered logistic regression                     Number of obs     =        611
                                                LR chi2(1)        =       0.02
                                                Prob > chi2       =     0.8789
Log likelihood = -981.51666                     Pseudo R2         =     0.0000

------------------------------------------------------------------------------
target_clavien |      Coef.   Std. Err.      z    P>|z|     [95% Conf. Interval]
---------------+--------------------------------------------------------------
     preop_asa |  -.0157401   .1033243    -0.15   0.879    -.2182519    .1867718
---------------+--------------------------------------------------------------
         /cut1 |  -.6308719   .1567989                      -.938192   -.3235517
         /cut2 |  -.4146385    .155474                     -.7193619   -.1099151
         /cut3 |  -.0626595   .1547901                     -.3660426    .2407235
         /cut4 |    1.63515   .1721419                      1.297758    1.972542
         /cut5 |   2.142996   .1874863                      1.775529    2.510462
         /cut6 |   2.721565   .2149934                      2.300186    3.142945
         /cut7 |   3.732672   .3010431                      3.142639    4.322706
------------------------------------------------------------------------------

. ologit target_clavien preop_weightloss_bin

Iteration 0:   log likelihood = -981.52826
Iteration 1:   log likelihood = -981.24008
Iteration 2:   log likelihood = -981.24007

Ordered logistic regression                     Number of obs     =        611
                                                LR chi2(1)        =       0.58
                                                Prob > chi2       =     0.4477
Log likelihood = -981.24007                     Pseudo R2         =     0.0003

------------------------------------------------------------------------------------
      target_clavien |      Coef.   Std. Err.      z    P>|z|     [95% Conf. Interval]
---------------------+--------------------------------------------------------------
preop_weightloss_bin |  -.1319197   .1738759    -0.76   0.448    -.4727102    .2088708
---------------------+--------------------------------------------------------------
               /cut1 |  -.6423225   .0944462                     -.8274336   -.4572114
               /cut2 |  -.4259735   .0923567                     -.6069893   -.2449577
               /cut3 |  -.0734346   .0906105                     -.2510279    .1041586
               /cut4 |   1.626328    .116509                      1.397975    1.854682
               /cut5 |   2.134391   .1381718                      1.863579    2.405203
               /cut6 |   2.713235    .173589                      2.373007    3.053463
               /cut7 |   3.724302   .2728953                      3.189437    4.259166
------------------------------------------------------------------------------------

. ologit target_clavien preop_fev1_bin

Iteration 0:   log likelihood = -981.52826
Iteration 1:   log likelihood =  -980.5515
Iteration 2:   log likelihood = -980.55133
Iteration 3:   log likelihood = -980.55133

Ordered logistic regression                     Number of obs     =        611
                                                LR chi2(1)        =       1.95
                                                Prob > chi2       =     0.1622
Log likelihood = -980.55133                     Pseudo R2         =     0.0010

------------------------------------------------------------------------------
target_clavien |      Coef.   Std. Err.      z    P>|z|     [95% Conf. Interval]
---------------+--------------------------------------------------------------
preop_fev1_bin |   .2468209   .1765836     1.40   0.162    -.0992766    .5929184
---------------+--------------------------------------------------------------
         /cut1 |  -.5568789   .0930141                     -.7391833   -.3745745
         /cut2 |  -.3402201   .0911587                     -.5188879   -.1615522
         /cut3 |    .011778   .0898081                     -.1642427    .1877987
         /cut4 |   1.711715    .117837                      1.480759    1.942672
         /cut5 |   2.221344   .1398448                      1.947253    2.495435
         /cut6 |   2.801263   .1752887                      2.457703    3.144822
         /cut7 |   3.812918   .2740493                      3.275791    4.350045
------------------------------------------------------------------------------

. ologit target_clavien preop_fev1_tot

Iteration 0:   log likelihood = -981.52826
Iteration 1:   log likelihood = -979.46657
Iteration 2:   log likelihood = -979.46574
```

```
Iteration 3:   log likelihood = -979.46574

Ordered logistic regression                     Number of obs     =        611
                                                LR chi2(1)        =       4.13
                                                Prob > chi2       =     0.0423
Log likelihood = -979.46574                     Pseudo R2         =     0.0021

--------------------------------------------------------------------------------
target_clavien |      Coef.   Std. Err.      z    P>|z|     [95% Conf. Interval]
---------------+----------------------------------------------------------------
preop_fev1_tot |  -.1886035   .0932769    -2.02   0.043    -.3714229   -.0057842
---------------+----------------------------------------------------------------
         /cut1 |  -1.188106   .2980295                     -1.772233   -.6039789
         /cut2 |  -.9703782   .2965848                     -1.551674   -.3890826
         /cut3 |   -.618018   .2957912                     -1.197758   -.0382779
         /cut4 |   1.081806   .3026641                      .4885953    1.675017
         /cut5 |   1.591114   .3109039                      .9817534    2.200474
         /cut6 |   2.171532   .3273603                      1.529918    2.813147
         /cut7 |   3.184538   .3885131                      2.423067     3.94601
--------------------------------------------------------------------------------

. ologit target_clavien preop_vc_bin

Iteration 0:   log likelihood = -981.52826
Iteration 1:   log likelihood = -981.19362
Iteration 2:   log likelihood =  -981.1936

Ordered logistic regression                     Number of obs     =        611
                                                LR chi2(1)        =       0.67
                                                Prob > chi2       =     0.4133
Log likelihood =  -981.1936                     Pseudo R2         =     0.0003

--------------------------------------------------------------------------------
target_clavien |      Coef.   Std. Err.      z    P>|z|     [95% Conf. Interval]
---------------+----------------------------------------------------------------
  preop_vc_bin |   .1528855   .1868424     0.82   0.413    -.2133189    .5190898
---------------+----------------------------------------------------------------
         /cut1 |  -.5817172   .0918089                     -.7616593   -.4017751
         /cut2 |  -.3653163   .0898696                     -.5414576   -.1891751
         /cut3 |  -.0136766   .0882937                     -.1867291     .159376
         /cut4 |   1.684506   .1160864                      1.456981    1.912031
         /cut5 |   2.193376   .1382898                      1.922333    2.464419
         /cut6 |   2.772504   .1739368                      2.431594    3.113414
         /cut7 |   3.783648   .2731155                      3.248352    4.318945
--------------------------------------------------------------------------------

. ologit target_clavien preop_vc_tot

Iteration 0:   log likelihood = -981.52826
Iteration 1:   log likelihood = -979.49341
Iteration 2:   log likelihood = -979.49249
Iteration 3:   log likelihood = -979.49249

Ordered logistic regression                     Number of obs     =        611
                                                LR chi2(1)        =       4.07
                                                Prob > chi2       =     0.0436
Log likelihood = -979.49249                     Pseudo R2         =     0.0021

--------------------------------------------------------------------------------
target_clavien |      Coef.   Std. Err.      z    P>|z|     [95% Conf. Interval]
---------------+----------------------------------------------------------------
  preop_vc_tot |   -.158622   .0789692    -2.01   0.045    -.3133988   -.0038453
---------------+----------------------------------------------------------------
         /cut1 |  -1.230946   .3203771                     -1.858873   -.6030182
         /cut2 |  -1.013086   .3188979                     -1.638114   -.3880573
         /cut3 |  -.6602034   .3178733                     -1.283224   -.0371832
         /cut4 |   1.039715   .3239606                       .404764    1.674666
         /cut5 |   1.548533   .3317715                       .898273    2.198793
         /cut6 |   2.129057   .3470875                      1.448778    2.809336
         /cut7 |   3.142086   .4051863                      2.347936    3.936237
--------------------------------------------------------------------------------

. ologit target_clavien psm_robot

Iteration 0:   log likelihood = -981.52826
Iteration 1:   log likelihood = -979.41838
Iteration 2:   log likelihood = -979.41765
Iteration 3:   log likelihood = -979.41765

Ordered logistic regression                     Number of obs     =        611
                                                LR chi2(1)        =       4.22
                                                Prob > chi2       =     0.0399
Log likelihood = -979.41765                     Pseudo R2         =     0.0022

--------------------------------------------------------------------------------
target_clavien |      Coef.   Std. Err.      z    P>|z|     [95% Conf. Interval]
---------------+----------------------------------------------------------------
     psm_robot |  -.4687046    .229703    -2.04   0.041    -.9189143   -.0184949
---------------+----------------------------------------------------------------
         /cut1 |  -.6694188   .0896567                     -.8451427    -.493695
         /cut2 |  -.4517284   .0872717                     -.6227778   -.2806791
         /cut3 |  -.0979531    .085441                     -.2654144    .0695081
         /cut4 |   1.605104   .1127251                      1.384167    1.826041
         /cut5 |   2.113214   .1350485                      1.848524    2.377904
         /cut6 |   2.692443   .1711465                      2.357002    3.027884
         /cut7 |    3.70471   .2712925                      3.172987    4.236434
--------------------------------------------------------------------------------

.
. ologit target_clavien preop_diag preop_age preop_sex preop_smoke preop_alcohol preop_khk preop_aht preop_vhf preop_mi
preop_revasc preop_pavk preop_copd preop_liver preop_crea_t
```

```
> ot preop_gfr_tot preop_quick_tot preop_albumin_tot preop_biliribin_tot preop_platelet_bin preop_platelet_tot
preop_leuko_bin preop_leuko_tot preop_diabetes preop_weight preop_he
> ight preop_bmi preop_ecog preop_tstage preop_neoadj preop_asa preop_weightloss_bin preop_fev1_bin preop_fev1_tot
preop_vc_bin preop_vc_tot psm_robot

Iteration 0:   log likelihood = -981.52826
Iteration 1:   log likelihood = -958.55393
Iteration 2:   log likelihood = -958.44063
Iteration 3:   log likelihood = -958.44059

Ordered logistic regression                     Number of obs     =        611
                                                LR chi2(36)       =      46.18
                                                Prob > chi2       =     0.1192
Log likelihood = -958.44059                     Pseudo R2         =     0.0235

-------------------------------------------------------------------------------------
     target_clavien |      Coef.   Std. Err.      z    P>|z|     [95% Conf. Interval]
--------------------+----------------------------------------------------------------
         preop_diag |   .2958104   .2107815     1.40   0.160    -.1173137    .7089345
          preop_age |   .0240988    .013692     1.76   0.078     -.002737    .0509345
          preop_sex |   .3190734   .3189166     1.00   0.317    -.3059916    .9441384
        preop_smoke |   .0721571    .117763     0.61   0.540    -.1586542    .3029683
      preop_alcohol |  -.1200487   .0839121    -1.43   0.153    -.2845134    .0444159
          preop_khk |   .4072216   .3313026     1.23   0.219    -.2421195    1.056563
          preop_aht |  -.1012486   .1674146    -0.60   0.545    -.4293753     .226878
          preop_vhf |    .278302    .303634     0.92   0.359    -.3168097    .8734137
           preop_mi |   .0430775   .3786773     0.11   0.909    -.6991165    .7852714
       preop_revasc |  -.5662181   .4275882    -1.32   0.185    -1.404276    .2718395
         preop_pavk |   .0043689   .3989792     0.01   0.991    -.7776159    .7863537
         preop_copd |   .0532825   .2323899     0.23   0.819    -.4021934    .5087584
        preop_liver |  -.2208616   .3403901    -0.65   0.516    -.8880139    .4462907
     preop_crea_tot |    .908322   .7982525     1.14   0.255    -.6562242    2.472868
      preop_gfr_tot |   .0034878   .0101603     0.34   0.731     -.016426    .0234016
    preop_quick_tot |   .0022171   .0054449     0.41   0.684    -.0084547     .012889
  preop_albumin_tot |   .0465906   .0196391     2.37   0.018     .0080988    .0850824
preop_biliribin_tot |  -.0080342   .3634425    -0.02   0.982    -.7203684       .7043
 preop_platelet_bin |  -.1303041   .3266456    -0.40   0.690    -.7705177    .5099096
 preop_platelet_tot |  -.0016849   .0010025    -1.68   0.093    -.0036498    .0002799
    preop_leuko_bin |  -.2722625   .2575382    -1.06   0.290     -.777028    .2325031
    preop_leuko_tot |   .0660022   .0351129     1.88   0.060    -.0028178    .1348223
     preop_diabetes |   .2074778   .2453426     0.85   0.398    -.2733849    .6883404
       preop_weight |   .0148662   .0489453     0.30   0.761    -.0810648    .1107972
       preop_height |  -.9418378   4.894166    -0.19   0.847    -10.53423    8.650551
          preop_bmi |  -.0253877   .1525284    -0.17   0.868    -.3243379    .2735626
         preop_ecog |  -.0472536    .117322    -0.40   0.687    -.2772006    .1826933
       preop_tstage |   .1196907   .1527467     0.78   0.433    -.1796873    .4190686
       preop_neoadj |  -.0519718   .1213627    -0.43   0.668    -.2898383    .1858947
          preop_asa |  -.0173371   .1176142    -0.15   0.883    -.2478568    .2131825
preop_weightloss_bin |  -.0441396   .1882343    -0.23   0.815    -.4130722    .3247929
     preop_fev1_bin |   .4148451   .2705546     1.53   0.125     -.115432    .9451223
     preop_fev1_tot |   .0826848   .2108457     0.39   0.695    -.3305651    .4959347
       preop_vc_bin |  -.2448852   .2612089    -0.94   0.348    -.7568454    .2670749
       preop_vc_tot |  -.1000473   .1767692    -0.57   0.571    -.4465084    .2464139
          psm_robot |  -.4026424   .2619737    -1.54   0.124    -.9161014    .1108167
--------------------+----------------------------------------------------------------
              /cut1 |   3.110269   8.995473                     -14.52053    20.74107
              /cut2 |   3.339449   8.995768                     -14.29193    20.97083
              /cut3 |   3.713643   8.996308                      -13.9188    21.34608
              /cut4 |   5.491483    8.99736                     -12.14302    23.12598
              /cut5 |   6.012208   8.996894                     -11.62138     23.6458
              /cut6 |   6.606966   8.997565                     -11.02794    24.24187
              /cut7 |   7.633713   9.000814                     -10.00756    25.27498
-------------------------------------------------------------------------------------

.
```
