# Supplementary material for: Robot-assisted minimally invasive esophagectomy (RAMIE) vs. hybrid minimally invasive esophagectomy: propensity score matched short-term outcome analysis of a European high-volume center
Source: Surg Endosc. 2022 May 3;36(10):7747–55. doi: 10.1007/s00464-022-09254-2 (PMC9485091; doi:10.1007/s00464-022-09254-2)
Supplement: Supplementary file 3 — Supplementary file3 (PDF 29 kb) [file 464_2022_9254_MOESM3_ESM.pdf]

```
  ___  ____  ____  ____  ____ (R)
 /__    /   ____/   /   ____/
___/   /   /___/   /   /___/   15.0   Copyright 1985-2017 StataCorp LLC
  Statistics/Data Analysis            StataCorp
                                      4905 Lakeway Drive
     Special Edition                  College Station, Texas 77845 USA
                                      800-STATA-PC        http://www.stata.com
                                      979-696-4600        stata@stata.com
                                      979-696-4601 (fax)

. tabulate psm_robot target_ai , chi2

           |       target_ai
 psm_robot |         0          1 |     Total
-----------+----------------------+----------
         0 |        56         15 |        71 
         1 |        65          6 |        71 
-----------+----------------------+----------
     Total |       121         21 |       142 

          Pearson chi2(1) =   4.5266   Pr = 0.033

. tabulate psm_robot target_conversion , chi2

           |   target_conversion
 psm_robot |         0          1 |     Total
-----------+----------------------+----------
         0 |        71          0 |        71 
         1 |        70          1 |        71 
-----------+----------------------+----------
     Total |       141          1 |       142 

          Pearson chi2(1) =   1.0071   Pr = 0.316

. tabulate psm_robot target_taa , chi2

           |      target_taa
 psm_robot |         0          1 |     Total
-----------+----------------------+----------
         0 |        60         11 |        71 
         1 |        64          7 |        71 
-----------+----------------------+----------
     Total |       124         18 |       142 

          Pearson chi2(1) =   1.0179   Pr = 0.313

. tabulate psm_robot target_pneumonia , chi2

           |   target_pneumonia
 psm_robot |         0          1 |     Total
-----------+----------------------+----------
         0 |        65          6 |        71 
         1 |        65          6 |        71 
-----------+----------------------+----------
     Total |       130         12 |       142 

          Pearson chi2(1) =   0.0000   Pr = 1.000

. tabulate psm_robot target_r_status , chi2

           |    target_r_status
 psm_robot |         0          1 |     Total
-----------+----------------------+----------
         0 |        70          1 |        71 
         1 |        67          4 |        71 
-----------+----------------------+----------
     Total |       137          5 |       142 

          Pearson chi2(1) =   1.8657   Pr = 0.172

. 
. ttest target_icu_days , by(psm_robot)

Two-sample t test with equal variances
------------------------------------------------------------------------------
   Group |     Obs        Mean    Std. Err.   Std. Dev.   [95% Conf. Interval]
---------+--------------------------------------------------------------------
       0 |      71    5.605634    .5742562    4.838769    4.460316    6.750952
       1 |      71    3.197183    .3413901    2.876604    2.516302    3.878064
---------+--------------------------------------------------------------------
combined |     142    4.401408    .3479552    4.146364    3.713525    5.089292
---------+--------------------------------------------------------------------
    diff |            2.408451    .6680699                1.087641    3.729261
------------------------------------------------------------------------------
    diff = mean(0) - mean(1)                                      t =   3.6051
Ho: diff = 0                                     degrees of freedom =      140

    Ha: diff < 0                 Ha: diff != 0                 Ha: diff > 0
 Pr(T < t) = 0.9998         Pr(|T| > |t|) = 0.0004          Pr(T > t) = 0.0002

. ttest op_duration , by(psm_robot)

Two-sample t test with equal variances
------------------------------------------------------------------------------
   Group |     Obs        Mean    Std. Err.   Std. Dev.   [95% Conf. Interval]
---------+--------------------------------------------------------------------
       0 |      71           0           0           0           0           0
       1 |      71    380.6338    7.647023    64.43496    365.3823    395.8853
---------+--------------------------------------------------------------------
combined |     142    190.3169    16.47418    196.3125    157.7486    222.8852
```

```
---------+--------------------------------------------------------------------
    diff |           -380.6338    7.647023               -395.7524   -365.5152
------------------------------------------------------------------------------
    diff = mean(0) - mean(1)                                      t = -49.7754
Ho: diff = 0                                     degrees of freedom =      140

    Ha: diff < 0                 Ha: diff != 0                 Ha: diff > 0
 Pr(T < t) = 0.0000         Pr(|T| > |t|) = 0.0000          Pr(T > t) = 1.0000

. ttest op_elv , by(psm_robot)

Two-sample t test with equal variances
------------------------------------------------------------------------------
   Group |     Obs        Mean    Std. Err.   Std. Dev.   [95% Conf. Interval]
---------+--------------------------------------------------------------------
       0 |      71    135.4676    2.031476    17.11752    131.4159    139.5192
       1 |      71    190.1181    4.596151    38.72785    180.9514    199.2848
---------+--------------------------------------------------------------------
combined |     142    162.7928    3.400535    40.52205    156.0702    169.5155
---------+--------------------------------------------------------------------
    diff |           -54.65054    5.025087                -64.5854   -44.71567
------------------------------------------------------------------------------
    diff = mean(0) - mean(1)                                      t = -10.8755
Ho: diff = 0                                     degrees of freedom =      140

    Ha: diff < 0                 Ha: diff != 0                 Ha: diff > 0
 Pr(T < t) = 0.0000         Pr(|T| > |t|) = 0.0000          Pr(T > t) = 1.0000

. ttest target_los , by(psm_robot)

Two-sample t test with equal variances
------------------------------------------------------------------------------
   Group |     Obs        Mean    Std. Err.   Std. Dev.   [95% Conf. Interval]
---------+--------------------------------------------------------------------
       0 |      71    19.64124    1.121252    9.447833    17.40497    21.87751
       1 |      71    16.15493    .9898169    8.340346     14.1808    18.12906
---------+--------------------------------------------------------------------
combined |     142    17.89808    .7594865    9.050326    16.39663    19.39954
---------+--------------------------------------------------------------------
    diff |             3.48631    1.495641                .5293468    6.443273
------------------------------------------------------------------------------
    diff = mean(0) - mean(1)                                      t =   2.3310
Ho: diff = 0                                     degrees of freedom =      140

    Ha: diff < 0                 Ha: diff != 0                 Ha: diff > 0
 Pr(T < t) = 0.9894         Pr(|T| > |t|) = 0.0212          Pr(T > t) = 0.0106

. ttest target_ebl , by(psm_robot)

Two-sample t test with equal variances
------------------------------------------------------------------------------
   Group |     Obs        Mean    Std. Err.   Std. Dev.   [95% Conf. Interval]
---------+--------------------------------------------------------------------
       0 |      71           0           0           0           0           0
       1 |      71    229.6335    11.80055    99.43318    206.0981     253.169
---------+--------------------------------------------------------------------
combined |     142    114.8168    11.31645     134.851    92.44492    137.1886
---------+--------------------------------------------------------------------
    diff |           -229.6335    11.80055               -252.9639   -206.3032
------------------------------------------------------------------------------
    diff = mean(0) - mean(1)                                      t = -19.4596
Ho: diff = 0                                     degrees of freedom =      140

    Ha: diff < 0                 Ha: diff != 0                 Ha: diff > 0
 Pr(T < t) = 0.0000         Pr(|T| > |t|) = 0.0000          Pr(T > t) = 1.0000

. ttest target_lymphnode_positive , by(psm_robot)

Two-sample t test with equal variances
------------------------------------------------------------------------------
   Group |     Obs        Mean    Std. Err.   Std. Dev.   [95% Conf. Interval]
---------+--------------------------------------------------------------------
       0 |      71    1.535211    .3863001    3.255022      .76476    2.305663
       1 |      71    2.309859    .7440148     6.26918    .8259685     3.79375
---------+--------------------------------------------------------------------
combined |     142    1.922535    .4189443    4.992298    1.094311    2.750759
---------+--------------------------------------------------------------------
    diff |           -.7746479    .8383232               -2.432058     .882762
------------------------------------------------------------------------------
    diff = mean(0) - mean(1)                                      t =  -0.9240
Ho: diff = 0                                     degrees of freedom =      140

    Ha: diff < 0                 Ha: diff != 0                 Ha: diff > 0
 Pr(T < t) = 0.1785         Pr(|T| > |t|) = 0.3571          Pr(T > t) = 0.8215

. ttest target_lymphnode_yield , by(psm_robot)

Two-sample t test with equal variances
------------------------------------------------------------------------------
   Group |     Obs        Mean    Std. Err.   Std. Dev.   [95% Conf. Interval]
---------+--------------------------------------------------------------------
       0 |      71    32.19718    1.030251    8.681046    30.14241    34.25195
       1 |      71     36.8169     1.70268    14.34704    33.42101    40.21279
---------+--------------------------------------------------------------------
combined |     142    34.50704    1.010421    12.04056    32.50951    36.50458
---------+--------------------------------------------------------------------
    diff |           -4.619718    1.990109               -8.554271   -.6851653
------------------------------------------------------------------------------
    diff = mean(0) - mean(1)                                      t =  -2.3213
Ho: diff = 0                                     degrees of freedom =      140
```

```
    Ha: diff < 0                 Ha: diff != 0                 Ha: diff > 0
 Pr(T < t) = 0.0109         Pr(|T| > |t|) = 0.0217          Pr(T > t) = 0.9891

.
. ranksum target_clavien , by(psm_robot)

Two-sample Wilcoxon rank-sum (Mann-Whitney) test

   psm_robot |      obs    rank sum    expected
-------------+---------------------------------
           0 |       71      5641.5      5076.5
           1 |       71      4511.5      5076.5
-------------+---------------------------------
    combined |      142       10153       10153

unadjusted variance    60071.92
adjustment for ties    -4927.95
                     ----------
adjusted variance      55143.96

Ho: targe~en(psm_ro~t==0) = targe~en(psm_ro~t==1)
             z =   2.406
    Prob > |z| =   0.0161

.
```
